# Reductive opening of a cyclopropane ring in the Ni(II) coordination environment: a route to functionalized dehydroalanine and cysteine derivatives

**DOI:** 10.3762/bjoc.18.121

**Published:** 2022-09-08

**Authors:** Oleg A Levitskiy, Olga I Aglamazova, Yuri K Grishin, Tatiana V Magdesieva

**Affiliations:** 1 Lomonosov Moscow State University, Dept. of Chemistry, Leninskie Gory 1/3, Moscow 119991, Russian Federation

**Keywords:** amino acids, cathodic cyclopropane opening, cysteine derivatives, Ni–Schiff base complexes, stereoselective electrosynthesis, voltammetric testing

## Abstract

The involvement of an α,α-cyclopropanated amino acid in the chiral Ni(II) coordination environment in the form of a Schiff base is considered as a route to electrochemical broadening of the donor–acceptor cyclopropane concept in combination with chirality induction in the targeted products. A tendency to the reductive ring-opening and the follow-up reaction paths of thus formed radical anions influenced by substituents in the cyclopropane ring are discussed. Optimization of the reaction conditions opens a route to the non-proteinogenic amino acid derivatives containing an α–β or β–γ double C=C bond in the side chain; the regioselectivity can be tuned by the addition of Lewis acids. One-pot combination of the reductive ring opening and subsequent addition of thiols allows obtaining the cysteine derivatives in practical yields and with high stereoselectivity at the removed β-stereocenter.

## Introduction

Electrochemistry provides a direct access to highly reactive species by means of harnessing electrons or electron holes as reagents [1–2]. This capacity can be efficiently exploited in organic synthesis for rational construction of complex multifunctional molecules [3–8].

Recently, we elaborated a versatile electrochemical approach for the stereoselective functionalization of a side chain of amino acids involved in the Ni(II) chiral coordination environment [9–15]. A combination of redox-activity and chirality provided by the Ni–Schiff base template, supported with the protection from redox-destruction of the amino acid skeleton, makes the suggested approach a convenient route to various types of non-proteinogenic amino acids [9–10,12–13]. Recently, several practical approaches to α,α-cyclopropanated amino acids in the form of Ni(II)–Schiff base complexes were suggested [16–19], including electrochemical ones [15]. Cyclopropane-containing amino acids are important components of various pharmaceuticals [20–21] and bio-additives [22]. Meanwhile, the cyclopropane fragment not only provides targeted pharmacophoric properties of the bio-active molecule [23] but also opens a route to its further functionalization, being a building block with wide variety of reactivity. A donor–acceptor cyclopropane concept suggested in the 1980s [24] became extremely popular in the recent decade [25–26]. Donor–acceptor cyclopropanes constitute an easily available equivalent of all-carbon 1,3-zwitter-ions used in targeted synthesis of various alicyclic as well as carbo- or heterocyclic compounds [27–30]. The reactive synergy of the three-membered ring and the C–C bond polarization due to donor–acceptor substituents contribute to the rich chemistry of these compounds. However, strict requirements for the nature of substituents somewhat narrow the applicability of the method.

The electrochemical one-electron opening of a cyclopropane ring results in the formation of an ion-radical species instead of zwitter-ions, thus creating preconditions for a different type of reactivity. Such processes are much less investigated.

The very first example of anodic cyclopropane ring opening was reported by Shono [31]. This publication sparked interest in this topic; a number of publications appeared but the reaction scope was mainly limited to rather simple compounds containing methyl and phenyl substituents [32–37].

When discussing examples of reductive cyclopropane ring opening, one should refer to early publications concerning the carbonyl- and nitro-substituted compounds [38–42]. The principal possibility of the process was demonstrated but the synthetic potential of the method was not sufficiently implemented.

Great success of the donor–acceptor cyclopropane concept in organic synthesis stimulated a renaissance of interest to electrochemical ring opening. Quite recently, two publications from the Werz group appeared concerning anodic activation of donor–acceptor cyclopropanes followed by their functionalization with arenes [43] or yielding oxy-ketones or 1,2-dioxanes [44]; the latter process was inspired by a previous report of Buriez [45]. The anodic fluorination of arylcyclopropane derivatives was reported recently [46–47], difluorinated or oxyfluorinated products were obtained [47]. Notably, anodic fluorination of cyclopropane derivatives bearing arylthio groups gives rise to a variety of possible reaction paths yielding monofluorinated sulfoxides as well as ring-opened fluorinated products [46].

Thus, recent publications on the topic concern only anodic opening of a cyclopropane ring followed by further functionalization of the carbon skeleton, demonstrating great synthetic potential of the process.

Electrochemical cyclopropane opening followed by stereoselective functionalization has not been probed as yet. Herein, reductive three-membered ring opening in the chiral α,α-cyclopropanated amino acids involved in the Ni(II)–Schiff base coordination environment is reported. Follow-up transformations of thus formed radical anions will be discussed, including reactions with electrophiles, intramolecular cyclization and disproportionation process. The synthetic viability of the approach will be considered. A one-pot multistep synthetic protocol is suggested, based on addition of thiols to the mixture of isomeric alkenes formed in an electroreductive opening of a cyclopropane ring in α,α-cyclopropanated amino acids yielding the cysteine derivatives in practical yields and with high stereoselectivity at the removed β-stereocenter. Thus, the present paper is a further development of the extended research on electrochemically induced stereoselective transformations in the Ni(II) coordination environment yielding structurally and functionally novel types of tailor-made amino acids.

## Results and Discussion

### Voltammetry study

**Figure 1:**
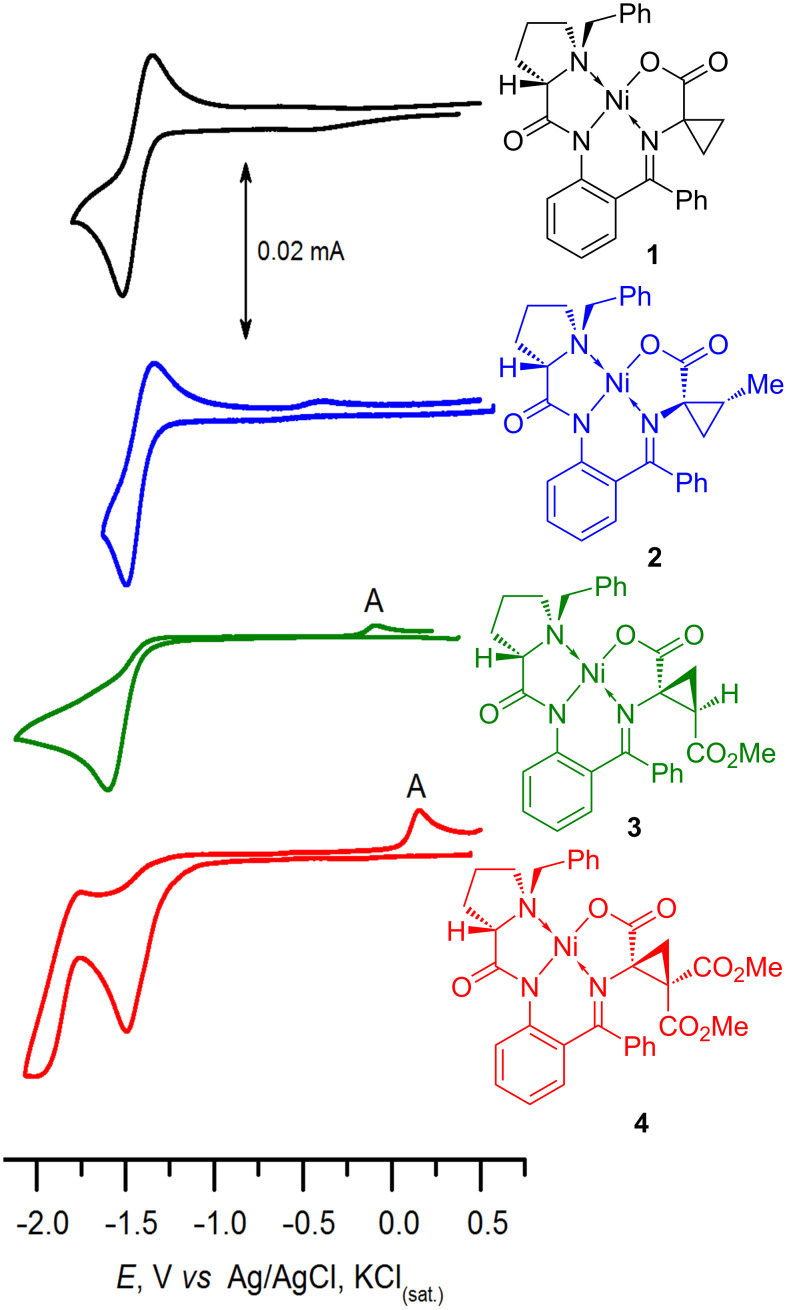
Cyclic voltammograms obtained for complexes **1** (black), **2** (blue), **3** (green), **4** (red) (MeCN, 0.05 M Bu_4_NBF_4_, 100 mV/s, Pt).

As models, a series of Ni(II)–Schiff base complexes containing α,α-cyclopropanated amino acids was synthesized (see Figure 1). Complexes **1**–**3** containing an unsubstituted cyclopropane ring (**1**) or bearing Me (**2**) and COOMe (**3**) groups were obtained using previously reported protocols [15,19]. Complex **4** is new.

**Scheme 1:**
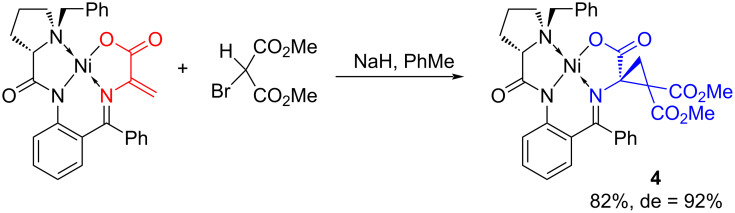
Synthesis of complex **4**.

Compound **4** was obtained by the reaction of an electrophilic dehydroalanine complex with a bromomalonate anion (Scheme 1). The reaction proceeds smoothly at room temperature giving rise to cyclopropane **4** in excellent diastereoselectivity (de = 92%) and high yield.

**Figure 2:**
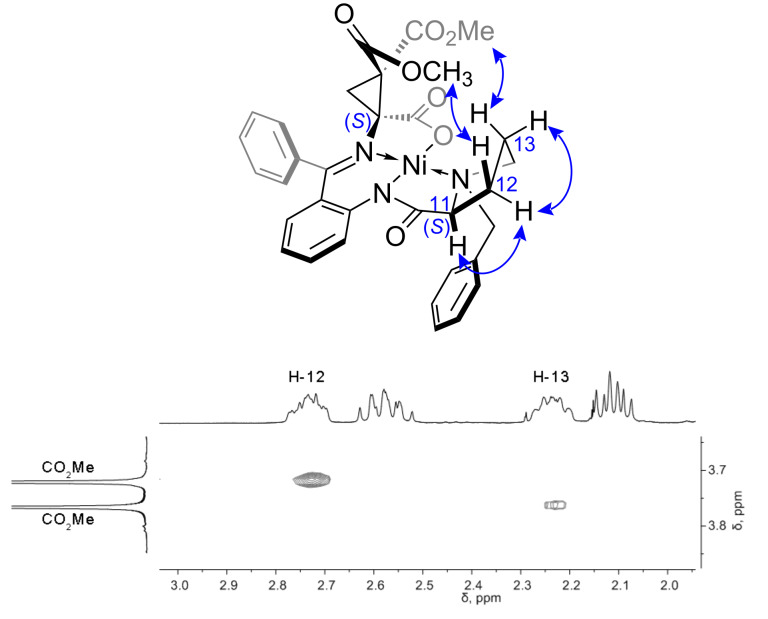
Key correlations in the NOESY spectrum of complex (*S*)-**4** and the corresponding characteristic fragment of the spectrum (full spectrum is given in Supporting Information File 1).

The optical purity of the starting compounds was confirmed by comparison of the optical rotation data with previously published values (see Supporting Information File 1). Complex **4** was easily separated from the minor diastereomer by column chromatography. The structure and purity of (*S*)-**4** was confirmed by NMR data, including HMBC, HSQC and COSY 2D techniques. The assignment of the α-stereocenter as (*S*) was based on the NOESY data (see Supporting Information File 1 and Figure 2). The protons of the ester group linked to the cyclopropane moiety exhibit correlation with the methylene protons of proline in the NOESY spectrum. Thus, the ester groups are at the same side of the Ni coordination plane as the proline methylene groups indicating the (*S*) configuration of the α-stereocenter. The large positive value of the specific rotation ([α]_D_ +1770) additionally supports α-(*S*) configuration since positive [α]_D_ values are characteristic for the Ni–Schiff base complexes of (*S*)-*N*-(*N*-benzylprolyl)aminobenzophenone and ʟ-amino acids [48].

To choose the most promising candidates for electrochemical three-membered ring opening, a voltammetric study was performed.

As it has been shown in our previous publications [49–51], the electrochemical behavior and the orbitals location sites are dependent on the type of the amino acid involved in the Schiff base complex. The LUMOs of the glycine, alanine, serine and cysteine derivatives are formed as an antibonding combination of the Ni *d_x_*_²-_*_y_*_²_ orbital with the group orbitals of the ligands; the π* orbital of the imine and the π orbital of the phenylene fragments are also partially involved. Reduction occurs at similar potential values and is almost independent on the substituent at the α-carbon atom in the amino acid fragment. Electrochemical reduction of complexes containing an unsubstituted cyclopropane ring (**1**) or bearing Me (**2**) substituents is similar to that for α-alkyl derivatives: they exhibit reversible one-electron reduction (the radical anions formed are relatively stable, at least in the CV time scale) at close potential values (*E*_1/2_ = −1.42 V (**1**), −1.42 V (**2**) ; for comparison: *E*_1/2_ for AlaNi = −1.53 V [52] vs Ag/AgCl, KCl_(sat.)_).

The complexes containing one (**3**) or two (**4**) electron-deficient COOMe groups in the cyclopropane moiety may be expected to be more prone to the ring opening, and the irreversibility of the cathodic redox process observed in the voltammograms (*E*_pc_ = −1.60 V vs Ag/AgCl, KCl_(sat.)_ for **3** and *E*_pc_ = −1.50 V for **4**; Figure 1) supported the suggestion. In the reverse anodic scan, a new oxidation peak (**A** in Figure 1; *E*_p_^A^ = −0.06 V (**3**), 0.16 V (**4**) vs Ag/AgCl, KCl_(sat.)_) can be observed for both complexes **3** and **4** (though in the latter case it is more intensive); more likely, the peak corresponds to reoxidation of the anionic species formed after the ring opening. The reduction patterns for compounds **3** and **4** differ significantly. In the voltammogram corresponding to complex **4** containing two COOMe groups, two consecutive reduction peaks can be observed. The more cathodic peak is reversible (*E*_1/2_ = −1.87 V vs Ag/AgCl, KCl_(sat.)_). Analysis of semi-differential curves (see Supporting Information File 1) indicates that the consumption of electrons at the first reduction peak is independent on the potential scan rate. In contrast, the more cathodic peak in semi-differential voltammogram gradually decreases with the scan rate increase. Thus, the second peak should be assigned to reduction of the product of the chemical step following the first reduction process.

### Preparative electrolysis

**Scheme 2:**
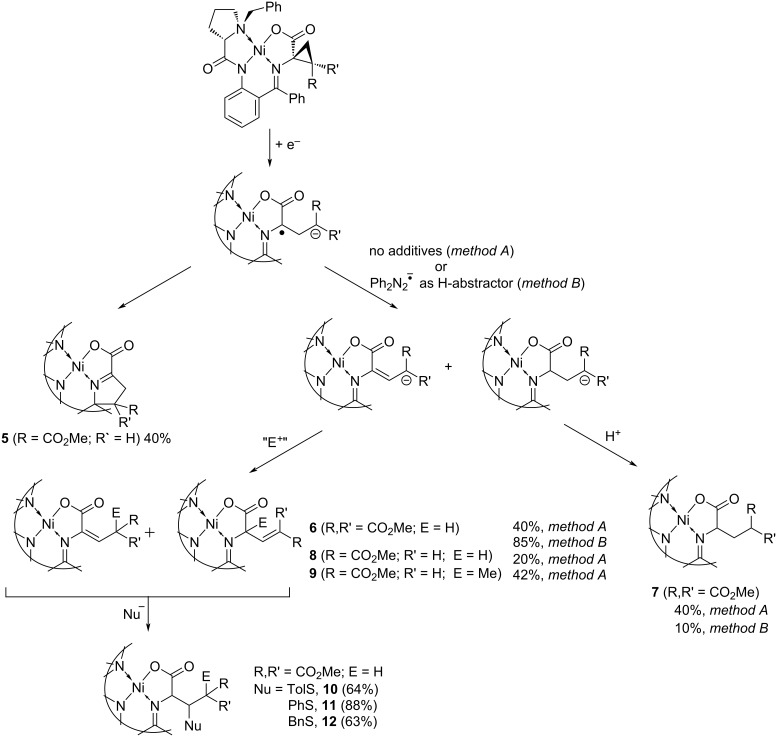
Reductive three-membered ring-opening and follow-up chemical steps.

Based on the voltammetry results, complexes **3** and **4** were chosen as the models for the preparative study. The electrolysis was performed in a two-compartment electrochemical cell in DMF using a glassy carbon plate as a working electrode and an iron rod as an auxiliary electrode. The process was carried out in the potentiostatic mode at a potential of 100 mV more cathodic than the peak potential value observed in the voltammogram; a charge corresponding to 1 mol equivalent of the starting complex was passed through the solution. The color of the solution was gradually changed from deep red to dark violet, typically for the anionic complexes. The solutions obtained were ESR-silent, indicating formation of the closed-shell species. The UV–vis study showed an intensive absorption at 546 nm for **3** and at 519 nm for **4**. The significant bathochromic shift as compared to the deprotonated glycine complex (λ_max_ = 458 nm [9]) indicates an elongation of the conjugation chain and formation of the anionic complex which can be considered as a vinylog of the parent glycine derivative (Scheme 2)

The anionic species formed in the electrolysis of complex **3** can be protonated using acetic acid (p*K*_a_ in DMSO = 12.3 [53]), in contrast to their counterparts formed from complex **4**. In the latter case, a stronger protonating agent is required, e.g., PhNEt_2_·HCl (p*K*_a_ in DMSO = 2.45 for PhN^+^HMe_2_ [53]). The p*K*_a_ value of **6** determined in DMSO solution using the UV–vis method (see Supporting Information File 1 for the details) is 5.1, indicating high stability of the anion.

### Synthesis of dehydroalanine derivatives **6**

The reaction products were isolated by column chromatography and analyzed using spectral methods. Both alkene and hydrogenated complexes are formed in the equimolar ratio, indicating disproportionation of the radical anions formed. The hydrogenated complexes **7** are of less synthetic value since they are more easily available than the corresponding substituted dehydroalanine derivatives. Additionally, the latter are of interest due to bioactivity [54–55]. To increase the yield of the alkene complexes, the addition of an external “H-abstractor” may be helpful, to suppress disproportionation. A possible candidate may be a reduced radical form of azobenzene. Indeed, the preparative electrolysis performed in the presence of the equimolar Ph_2_N_2_ additive changed the relative ratio of alkene to hydrogenated derivatives in favor of the former one (see Table 1).

**Table 1:** Yields of the alkene complexes **6** and the hydrogenated derivative are dependent on the electrolysis conditions used for the ring opening in complex **4** (8 mM) (GC, DMF, 0.09 M Bu_4_NBF_4_ or 0.8 M LiCl).

conditions					alkene complexes **6**		hydrogenated derivative **7** yield, %
		
supporting electrolyte, additives	WE potential	charge / 1 mol of **4**	cell type		yield, %	α-β/β-γ isomers ratio	

Bu_4_NBF_4_	−1.7 V	1 F	divided		40	1.5:1	40
Bu_4_NBF_4_, Ph_2_N_2_ (1 equiv)	−1.5 V	2 F	divided		85	1.5:1	10
Bu_4_NBF_4_, Ph_2_N_2_ (1.5 equiv)	−1.5 V	2.5 F	divided		85	1.5:1	10
LiCl	−1.4 V	1 F	divided		40	5:1	40
Bu_4_NBF_4_ electrogenerated Zn^2+^ or Mg^2+^	(galvanostatic regime)	8 F	undivided		50	54:1	12

**Figure 3:**
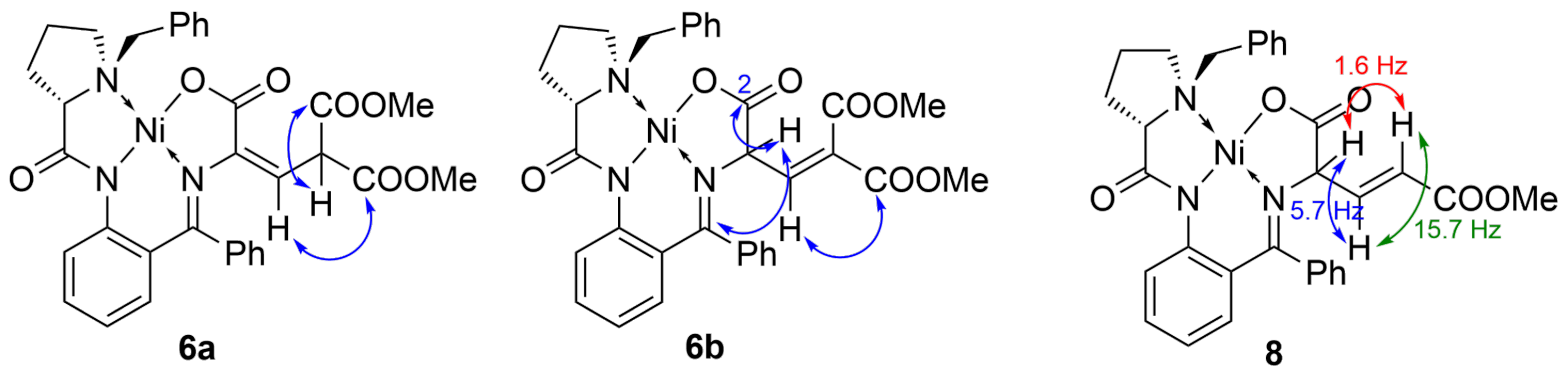
Correlations in the HMBC spectra of **6a** and **6b** and spin coupling constants in the ^1^H NMR spectrum of **8**.

As follows from Table 1, the azobenzene additive allows increasing the yield of the alkene complexes up to 85% suppressing formation of the hydrogenated complexes. Spectral NMR analysis of the reaction mixture showed that two isomeric alkenes (containing the α-β or β-γ double bond) are formed. In the isomers, two protons of the amino acid side chain create an AB system; in the α-β isomer, both protons show correlations in the HMBC spectrum with the C atoms of the COOMe groups, whereas in the β-γ isomer, only one H correlates with the COOMe and the other H atom correlates with the Schiff and carboxylic carbons (see Figure 3 and Supporting Information File 1).

The experimental ratio of the isomeric alkenes (1.5:1) is close to the calculated value predicted from their relative thermodynamic stability (1.3:1, see Supporting Information File 1). Notably, the coordination to the Lewis acid increases the regioselectivity of protonation in the allylic anions formed in the electrolysis. Thus, using LiCl as a supporting electrolyte increases the ratio to 5:1. An even more pronounced effect can be achieved if the electrolysis is performed in the undivided cell equipped with a Zn or Mg anode. In this case, the anodically generated Zn^2+^ or Mg^2+^ worked as Lewis acid and the isomeric α-β and β-γ alkene complexes are formed in 54:1 ratio (though the total yield is decreased to 50%).

In case of complex **3**, the isolated yield of the alkene complex was low (20%); the Ph_2_N_2_ additive gives only insignificant increase (27%). Analysis of the values of spin coupling constants (see Supporting Information File 1 for the details) testifies in favor of the β-γ isomer selective formation. The dominant reaction product formed in the reductive opening of the cyclopropane ring in complex **3** was new derivative **5** containing a five-membered ring (see below and Scheme 2).

### Intramolecular cyclization

**Scheme 3:**
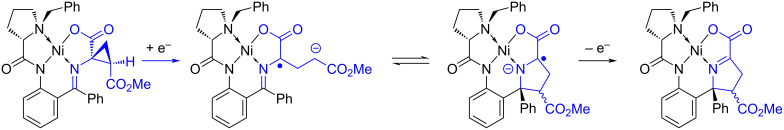
Electrochemically induced ring-opening followed by intramolecular cyclization.

The anionic species formed in the ring opening in complex **3** undergo fast intramolecular cyclization via the nucleophilic attack at the imine fragment yielding new complex **5** (Scheme 3), which was isolated in the form of two diastereomers in a total yield of 37%, indicating that this reaction route dominates. The compounds were characterized with NMR (2D technique, see Supporting Information File 1). Notably, insertion of the second COOR group in the starting cyclopropane complex **4** completely suppresses this reaction channel: intramolecular cyclization of the anions formed in the reductive cleavage of the three-membered ring was not observed. This may be attributed to the decreased nucleophilicity as well as to steric reasons.

### Reaction with electrophiles

Anions formed in the reductive ring opening in complex **4** are stable enough: they survive even in the presence of acetic acid and do not react with electrophiles (CH_3_I, benzaldehyde). In contrast, addition of external electrophiles in the reaction mixture obtained in the electrolysis of **3** launches an additional reaction path, along with intramolecular cyclization described above. Thus, addition of CH_3_I results in the formation of γ-methylated alkene complex **9** in the form of two diastereomers (5:1) in 42% yield.

The results obtained clearly indicate that follow-up functionalization of the anions formed after the ring opening with electrophiles (except H^+^) has low synthetic value due to multiple competing reaction channels observed in case of **3** and decreased nucleophilicity of **4**. Consequently, it seems reasonable to focus on the one-pot nucleophilic functionalization of the double bond of the dehydroalanine derivatives formed after the reductive ring opening and subsequent protonation. Such an approach opens a route to double functionalization of the amino acids side chain. Insertion of the sulfur-containing fragments is of special interest due to the bioactivity of such compounds [56–57]; thus, elaboration of new synthetic protocols to these multifunctional molecules is a topical problem.

**Scheme 4:**
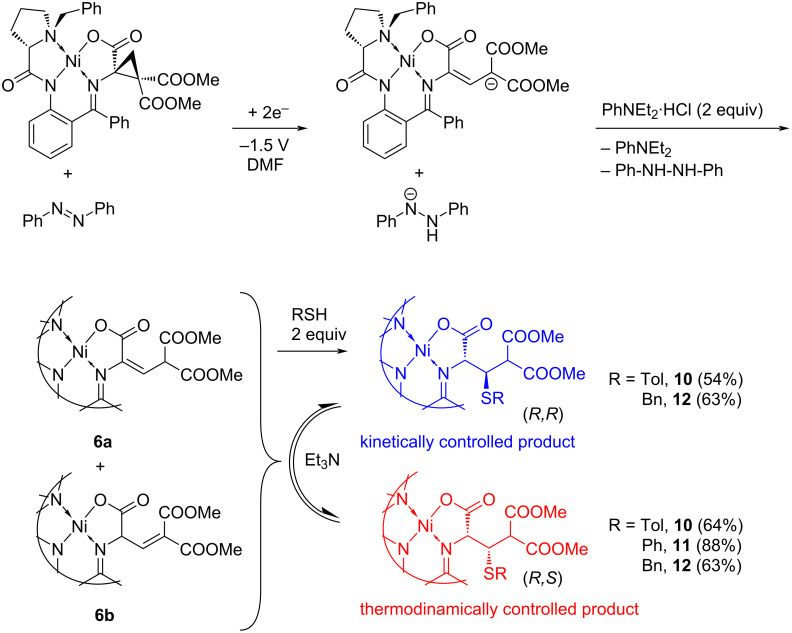
One-pot multistep approach to the cysteine derivatives.

Complex **4** was subjected to reductive ring opening at a potential of −1.5 V (Ag/AgCl, KCl_(sat.)_) in the presence of an equimolar amount of azobenzene as described above (two-compartment cell, DMF, Bu_4_NBF_4_, a glassy carbon WE, an iron wire as a CE). After 2 F/mol amount of electricity passed and subsequent protonation with PhNEt_2_·HCl, aryl- or benzylthiol was added. The reaction mixture was kept overnight and then the products were isolated using column chromatography and analyzed using spectral methods. The results obtained are given in Table 2 and Scheme 4.

**Table 2:** Yield and diastereomeric ratio of the cysteine derivatives obtained in a one-pot electrochemical reduction (method B: −1.5 V (Ag/AgCl, KCl_(sat.)_), **4** (8 mM), Ph_2_N_2_ (8 mM), 2F/mol, DMF) of complex **4** with subsequent thiol addition (PhNEt_2_·HCl (2 equiv); RSH (1 or 2 equiv), 24 h, rt).

	RSH	RSH (equiv)	(*R*,*S*):(*R*,*R*) dr	yield, %	additive	complex

1	TolSH	1	10:1	64	–	**10**
2	TolSH	2	1:5	54	–	**10**
3	TolSH	2	10:1	64	+ 1 equiv Et_3_N	**10**
4	PhSH	2	12:1	88	+ 1 equiv Et_3_N	**11**
5	BnSH	2	1:2.6	64	–	**12**
6	BnSH	2	pure (*R*,*S*)-isomer	42^a^	+ 1 equiv Et_3_N	**12**

^a^72 h, 40 °C.

The synthetic procedure was tested on three thiols, of both aromatic and aliphatic types. The first experiment with *p*-tolylthiol gave the targeted cysteine derivative in practical 64% isolated yield, along with some amount of the alkene complex (see Scheme 4). In an attempt to increase the yield of the cysteine derivatives, the amount of the thiol was doubled. Unexpectedly, this resulted in the inversion of the diastereomeric ratio (entry 2 in Table 2). To find a reason, the experiment was repeated in the presence of an additional base (1 mol equivalent of Et_3_N); the result was identical to that previously obtained for the equimolar **4**:*p*-tolylthiol mixture (compare entries 1 and 3 in Table 2). Thus, it seems reasonable to suggest that a base may induce epimerization of the product yielding the most thermodynamically stable cysteine derivative [58]. The suggestion was proven by the control experiment. An equimolar mixture of the diastereomeric cysteine complexes **10** were dissolved in DMF containing Et_3_N and TolSH (1:1) and left overnight at room temperature under argon. As a result, the (*R*,*S*):(*R*,*R*) diastereomeric ratio was changed from 1:1 to 13:1 in favor of the thermodynamically more stable (*R*,*S*) diastereomer.

The experiment with thiophenol performed under the same reaction conditions (a two-fold excess of thiol and the Et_3_N additive) gave the cysteine derivatives in 88% yield and with 12:1 diastereoselectivity; again, the (*R*,*S*) diastereomer was the dominant (entry 4, Table 2), in line with the previous results with tolylthiol.

In case of an aliphatic thiol (benzylthiol), the results were qualitatively similar. The diastereomeric ratio is inverted in favor of the kinetically controlled product when no Et_3_N is added into the solution (entry 5, Table 2). In contrast, pure (*R*,*S*) diastereomer was obtained when the solution containing 1 mol equiv of Et_3_N was kept for 72 h under slight heating (40 °C, entry 6); though in the expense of the yields decrease (a significant amount of the alkene (48% instead of 20% detected in entry 5) was also isolated from the reaction mixture).

Thus, the experiments indicated that the one-pot multistep experimentally simple procedure allows achieving high stereoselectivity at the removed β-stereocenter, what is not an easy task. In all cases, the targeted cysteine derivatives were isolated in practical yields. It should be noted that there is no need to separate the isomeric alkene complexes formed after the cyclopropane ring opening, they can be involved in the follow-up reaction with nucleophiles in the form of a mixture. This simplifies the synthetic procedure; the multistep process can be performed in an electrochemical cell, with the potential switching off prior to the addition of nucleophiles (thiols in our case).

**Figure 4:**
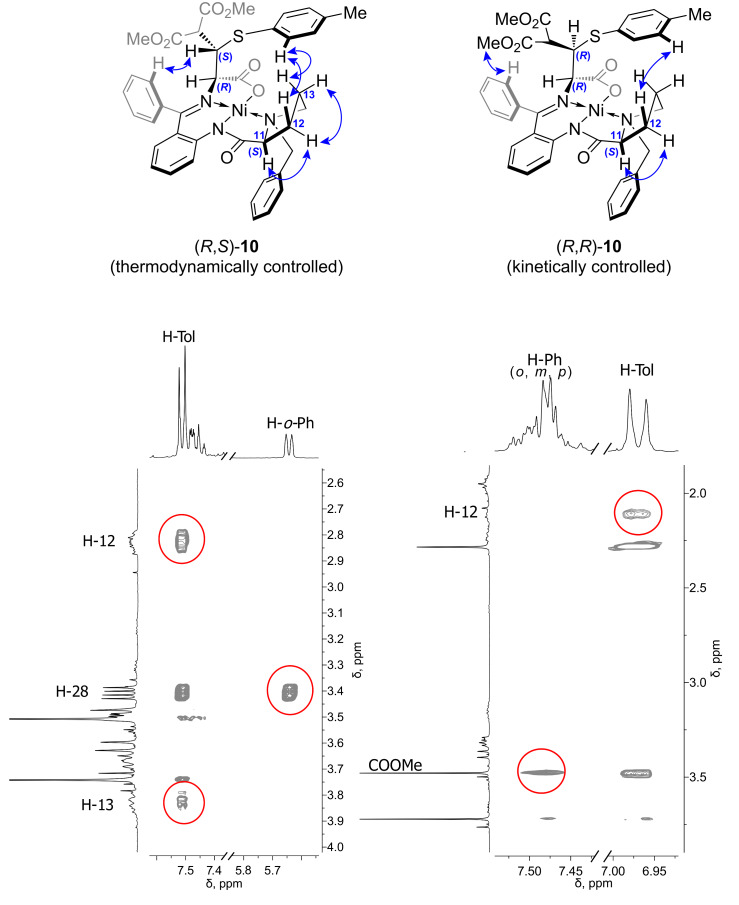
Characteristic correlations in the NOESY spectra of diastereomeric complexes **10** and the corresponding characteristic fragments of the spectra (full spectra are given in Supporting Information File 1); (*R*,*S*)-isomer (left) and (*R*,*R*)-isomer (right).

Complexes **10**–**12** were obtained as pure diastereomers (a set of signals corresponding to the individual compound is present in the ^1^H NMR spectrum in each case). Assigning of the relative configurations of newly formed α- and β-stereocenters in cysteine derivatives discussed above was performed using the NOESY spectra. It is illustrated in Figure 4 taking complex **10** as an example. A correlation between protons of the tolyl substituent in the side chain of the amino acid moiety and H-12 and H-13 protons of the proline methylene group is observed in the NOESY spectrum (Figure 4). Hence, the amino acid side chain and the proline methylenes are at the same side of the Ni coordination plane allowing to assign the configuration to the α-stereocenter as (*R*) in both thermodynamically and kinetically controlled isomers of **10**. The configurations of the β-stereocenter in the obtained diastereomers of **10** are different, as follows from the different correlations of the *ortho*-phenyl protons of the benzophenone moiety observed in the NOESY spectra of the diastereomers. The *ortho*-phenyl proton exhibits correlation with the β-H in case of the thermodynamically controlled isomer, whereas correlation with the ester methyl group is observed in the spectrum of the kinetically controlled isomer. This allows to unambiguously assign the relative configurations for these compounds (Figure 4).

Large positive values of specific rotation for both diastereomers of complexes **10**–**12** (see Supporting Information File 1) additionally support the α-(*R*) configuration. It was shown that positive values of [α]_D_ are inherent to the Ni–Schiff base complexes of (*S*)-*N*-(*N*-benzylprolyl)aminobenzophenone and ʟ-amino acids (i.e., (*R*)-cysteine derivatives) [48].

## Conclusion

Electroreductive opening of a cyclopropane ring in α,α-cyclopropanated amino acids in the form of Ni(II)–Schiff base complexes was studied. Preliminary voltammetry testing allowed to choose the most promising candidates for the preparative synthesis. The bulk electrolysis showed that substituents in the cyclopropane ring not only affect its tendency to the ring-opening, but also determine the follow-up reaction paths of thus formed radical anions. Possible reaction paths include disproportionation reaction yielding a mixture of alkenes and the corresponding hydrogenated derivatives, intramolecular cyclization and reaction with external electrophiles. Optimization of the reaction conditions opens a route to amino acid derivatives containing the α-β or β-γ double C=C bond in the side chain; the regioselectivity can be tuned by the addition of Lewis acids. This type of non-proteinogenic amino acid derivatives is not easily available but strongly required due to their bioactivity.

One-pot nucleophilic in situ functionalization of the double bond of dehydroalanine derivatives formed after the reductive ring opening and subsequent protonation opens a route to double functionalization of the amino acids side chain. Thus, addition of thiols to the mixture of alkenes formed in reductive opening of a cyclopropane ring in α,α-cyclopropanated amino acids allows obtaining the cysteine derivatives in practical yields and with high stereoselectivity at the removed β-stereocenter. The developed one-pot multistep procedure highlights new perspectives provided by combination of electrochemically broaden DA-cyclopropane concept and chirality induction within a metal coordination sphere.

Notably, the Ni template is an important component of the reaction. It is responsible for chirality induction and facilitates the cyclopropane ring opening, significantly decreasing the reduction potential value. It stabilizes the anion formed and serves as a directing group. Thus, the Ni–Schiff base platform creates an optimal balance between the covalent binding with the substrate (which does not “kill” its reactivity but precludes its redox destruction) with non-covalent interactions in the metal chiral coordination environment governing the reaction’s stereocontrol.

## Supporting Information

File 1 Additional experimental details, characterization data as well as NMR and MS spectra of synthesized compounds.
