# Supplementary material for: Reductive opening of a cyclopropane ring in the Ni(II) coordination environment: a route to functionalized dehydroalanine and cysteine derivatives
Source: Beilstein J Org Chem. 2022 Sep 8;18:1166–76. doi: 10.3762/bjoc.18.121 (PMC9475196; doi:10.3762/bjoc.18.121)
Supplement: File 1 — Additional experimental details, characterization data as well as NMR and MS spectra of synthesized compounds. [file Beilstein_J_Org_Chem-18-1166-s001.pdf]

BEILSTEIN JOURNAL OF ORGANIC CHEMISTRY

Supporting Information

for

Reductive opening of a cyclopropane ring in the Ni(II) coordination environment: a route to functionalized dehydroalanine and cysteine derivatives

Oleg A. Levitskiy, Olga I. Aglamazova, Yuri K. Grishin and Tatiana V. Magdesieva

Beilstein J. Org. Chem. **2022,** *18,* 1166–1176. doi:10.3762/bjoc.18.121

Additional experimental details, characterization data as well as NMR and MS spectra of synthesized compounds

License and Terms: This is a supporting information file under the terms of the Creative Commons Attribution License (https://creativecommons.org/licenses/by/4.0). Please note that the reuse, redistribution and reproduction in particular requires that the author(s) and source are credited and that individual graphics may be subject to special legal provisions.
The license is subject to the *Beilstein Journal of Organic Chemistry* terms and conditions: (https://www.beilstein-journals.org/bjoc/terms)

Contents

1. *Synthesis of complex **4*** S4
2. *Reductive ring opening of complex **4*** S4
3. *Reductive ring opening followed by the reaction with electrophiles (AcOH or MeI)* S5
4. *One-pot electrosynthesis of cysteine derivatives from complex **4*** S7
5. *Semi-integral voltammogram for complex **2** (100 mV/s)* S10
6. *Semi-differential voltammograms for complex **4** at various scan rates* S10
7. *Determination of the pK_a of complex **6*** S11
8. *Atom numeration and signal assignment in the NMR spectra of complex (S)-**4*** S13
9. *^{1}H NMR spectrum of complex (S)-**4*** S14
10. *^{13}C NMR spectrum of complex (S)-**4*** S15
11. *COSY spectrum of complex (S)-**4*** S16
12. *HSQC spectrum of complex (S)-**4*** S17
13. *HMBC spectrum of complex (S)-**4*** S18
14. *NOESY spectrum of complex (S)-**4*** S19
15. *Atom numeration and signal assignment in the NMR spectra of complex **5*** S20
16. *^{1}H NMR spectrum of complex **5**, diastereomer 1* S21
17. *^{13}C NMR spectrum of complex **5**, diastereomer 1* S22
18. *COSY spectrum of complex **5**, diastereomer 1* S23
19. *HSQC spectrum of complex **5**, diastereomer 1* S24
20. *HMBC spectrum of complex **5**, diastereomer 1* S25
21. *^{1}H NMR spectrum of complex **5**, diastereomer 2* S26
22. *^{13}C NMR spectrum of complex **5**, diastereomer 2* S27
23. *COSY spectrum of complex **5**, diastereomer 2* S28
24. *HSQC spectrum of complex **5**, diastereomer 2* S29
25. *HMBC spectrum of complex **5**, diastereomer 2* S30
26. *^{1}H NMR spectrum of complexes **6*** S31
27. *^{13}C NMR spectrum of complexes **6*** S32
28. *COSY spectrum of complexes **6*** S33
29. *HSQC spectrum of complexes **6*** S34
30. *HMBC spectrum of complexes **6*** S35
31. *^{1}H NMR spectrum of complex **8*** S36
32. *Atom numeration and signal assignment in the NMR spectra of complex **9*** S37
33. *^{1}H NMR spectrum of complex **9*** S38
34. *^{13}C NMR spectrum of complex **9*** S39
35. *COSY spectrum of complex **9*** S40

36. *HSQC spectrum of complex* ***9*** S41
37. *HMBC spectrum of complex* ***9*** S42
38. *Atom numeration and signal assignment in the NMR spectra of complex (R,S)-****10*** S43
39. *^{1}H NMR spectrum of complex (R,S)-****10*** S44
40. *^{13}C NMR spectrum of complex (R,S)-****10*** S45
41. *COSY spectrum of complex (R,S)-****10*** S46
42. *HSQC spectrum of complex (R,S)-****10*** S47
43. *HMBC spectrum of complex (R,S)-****10*** S48
44. *NOESY spectrum of complex (R,S)-****10*** S49
45. *^{1}H NMR spectrum of complex (R,R)-****10*** S50
46. *^{13}C NMR spectrum of complex (R,R)-****10*** S51
47. *COSY spectrum of complex (R,R)-****10*** S52
48. *HSQC spectrum of complex (R,R)-****10*** S53
49. *HMBC spectrum of complex (R,R)-****10*** S54
50. *NOESY spectrum of complex (R,R)-****10*** S55
51. *^{1}H NMR spectrum of complex (R,S)-****11*** S56
52. *^{13}C NMR spectrum of complex (R,S)-****11*** S57
53. *^{1}H NMR spectrum of complex (R,S)-****12*** S58
54. *^{13}C NMR spectrum of complex (R,S)-****12*** S59
55. *^{1}H NMR spectrum of complex (R,R)-****12*** S60
56. *^{13}C NMR spectrum of complex (R,R)-****12*** S61
57. *ESI-HRMS data for complex (S)-****4*** S62
58. *ESI-HRMS data for complex* **5***, diastereomer 1* S63
59. *ESI-HRMS data for complex* ***8*** S64
60. *ESI-HRMS data for complex* ***9*** S65
61. *ESI-HRMS data for complex (R,S)-****10*** S66
62. *Results of the quantum chemical calculations* S67

Experimental details

^{1}H (400.0 MHz) and ^{13}C (100.6 MHz) NMR spectra (including COSY, HMBC, HSQC) were recorded using an Aglient 400-MR spectrometer in $CDCl_3$. Chemical shifts were referenced to the nondeuterated aliquot of the solvent.

Mass spectra. CH_3CN (LC–MS grade) for ESI–MS experiments was ordered from Merck and used as received. Sodium formate (for HPLC) for calibration was ordered from Sigma-Aldrich. The samples for ESI–MS experiments were prepared in 1.8 mL glass vials/screw top caps with PTFE septa for HPLC experiments (Agilent Technologies).

Preparative electrolysis was performed with AutoLab PGSTAT100N potentiostat in a two-compartment cell of 10 mL volume. The WE was glassy carbon plate (300 mm^2); the CE was a stainless steel wire. The solution was stirred with an argon flow.

AvaSpec Avantes spectrometer was employed for *UV-vis* measurements.

Voltammetric experiments were performed with Biologic BP-300 potentiostat, in a ALS Co. three-electrode cell of 2 mL with a platinum wire counter electrode (CE) and anhydrous Ag/0.01 M $AgNO_3$ (MeCN) reference electrode (RE). Ferrocene was used as internal standard in each experiment and all measured potentials were converted to the Ag/AgCl,$KCl_{(sat.)}$ reference electrode (in the latter scale, the potential for the $Fc^{0/+}$ redox couple is equal to 0.475 V in acetonitrile). A Pt disk electrode with active surface area of 0.077 cm^2 was used as the working electrode (WE). The Pt electrode was polished with Al_2O_3 suspension SP-A 0.3 mm on a polishing pad (Metrohm, Germany), washed with sulfuric acid and rinsed with water and acetone. Hardware ohmic drop compensation was employed. All solutions were thoroughly deaerated by passing an argon flow through the solution prior to the CV experiments and above the solution during the measurements, the supporting electrolyte in all experiments was 0.1 M n-Bu_4NBF_4 (Aldrich, purity > 99%), which has been recrystallized from water and dried by gentle heating under reduced pressure (0.05 Torr) prior to use. Acetonitrile (AN, Aldrich spectroscopic quality, <0.02% water content) was distilled over P_2O_5 and stored under argon.

All reactants and solvents were commercially available and purified prior to experiments. Starting glycine and dehydroalanine complexes were prepared according to literature protocols[1]. Optical purity of the starting glycine complex was confirmed by comparison of the specific rotation value with previously published data[1] ($[\alpha]_D^{25}$ +2050, lit. +2006 in methanol). Complexes **1–3** were synthesized according to Ref[2]. To remove electroactive traces of chloroform, complexes were re-evaporated from toluene before electrolysis. Silicagel 60M 0.04–0.063 mm was used for column chromatography.

[1] Belokon Y.N., Tararov V.I., Maleev V.I., Savel'eva T.F., Ryzhov M.G. Tetrahedron: Asymmetry, **1998**, 9, 4249–4252.
Belokon Y.N., Sagyan A.S., Djamgaryan S.M., Bakhmutov V.I., Belikov V.M. Tetrahedron, **1988**, 44, 17, 5507-5514.

[2] Levitskiy, O. A.; Aglamazova, O. I.; Grishin, Y. K.; Nefedov, S. E.; Magdesieva, T. V. *Electrochim. Acta* **2022**, *409*, 139980. doi:10.1016/j.electacta.2022.139980.
Levitskiy, O. A.; Aglamazova, O. I.; Grishin, Y. K.; Paseshnichenko, K. A.; Soloshonok, V. A.; Moriwaki, H.; Magdesieva, T. V. *Dalt. Trans.* **2020**, *49* (25), 8636–8644. doi:10.1039/d0dt01578d.

Computational details

Stationary-point structures were optimized using the ORCA quantum chemistry package. A composite PBEh-3c method[3] accounting for basis set superposition error and dispersion effects was applied. A threshold of $1 \cdot 10^{-8}$ Hartree was used for SCF convergence; thresholds of $1 \cdot 10^{-6}$ Hartree and $3 \cdot 10^{-5}$ Hartree Bohr^{-1} on energy and RMS gradient, respectively, were employed in optimization procedures. SMD continuous solvation model[4] with dimethylformamide as solvent was applied.

Optical rotations were measured on a Krüss P8000 polarimeter.

1. *Synthesis of complex **4***

Synthesis was performed in an argon atmosphere using the standard Schlenk technique. The solution of Δ-AlaNi (1.142 g, 2.24 mmol) in toluene (10 mL) was degassed, then $BrCH(COOMe)_2$ (709 mg, 3.36 mmol, 1.5 equiv) was added. After 5 minutes NaH (136 mg, 3.4 mmol, 60% suspension in a mineral oil) was added. The reaction mixture was stirred at room temperature for 30 min. Afterwards, the reaction mixture was poured onto water; organic compounds were extracted with ethyl acetate. The combined organic fractions were dried over anhydrous Na_2SO_4; the solvent was evaporated under reduced pressure. The residue was separated using column chromatography. The first fraction was eluted with a $CHCl_3$/AcMe = 10:1 (the minor isomer); the second (major) fraction corresponding to the (*S*)-isomer was eluted with a $CHCl_3$/AcMe = 1:1 mixture). After removal of the solvent, (*R*)-**4** (49 mg, 3.5%) and (*S*)-**4** (1.17 g, 82%) were obtained.

(*S*)-**4**

HRMS (ESI): m/z 640.1582 (M+H$^+$, 640.1588 calculated for $C_{33}H_{32}N_3NiO_7$).

$[\alpha]_D^{25}$ +1770 (c = 0.037 g/100mL in MeOH)

^{1}H NMR ($CDCl_3$ δ, ppm): 8.21 (dd, ^3J = 8.7 Hz, ^4J = 0.9 Hz, 1H (H-8)), 8.16-8.11 (m, 2H (H-17,21)), 7.54-7.48 (m, 1H (H-25)), 7.47-7.40 (m, 2H (H-24,26)), 7.26-7.13 (m, 4H (H-18,20,23,27)), 7.10-7.00 (m, 2H (H-7,19)), 6.83 (dd, ^3J = 8.3 Hz, ^4J = 1.6 Hz, 1H (H-5)), 6.69-6.64 (m, 1H (H-6)), 4.33-4.17 (m, 2H (H-13,15)), 3.77 (s, 3H (H-31)), 3.72 (s, 3H (H-33)), 3.60-3.53 (m, 1H (H-14)), 3.44 (dd, ^3J = 11.0 Hz, ^3J = 5.8 Hz, 1H (H-11)), 3.31 (d, ^2J = 12.5 Hz, 1H (H-15)), 2.79-2.68 (m, 1H (H-12)), 2.64-2.51 (m, 1H (H-12)), 2.30-2.19 (m, 1H (H-13)), 2.16-2.06 (m, 1H (H-14)), 1.90 (d, ^2J = 7.6 Hz, 1H (H-29)), 0.93 (d, ^2J = 7.6 Hz, 1H (H-29)).

^{13}C NMR ($CDCl_3$ δ, ppm): 180.31 (C-10), 172.49 (C-3), 171.54 (C-1), 165.90 (C-30), 165.54 (C-32), 143.73 (C-9), 134.84 (C-22), 134.36 (C-5), 133.97 (C-16), 133.25 (C-7), 131.21 (C-17,21), 130.55 (C-23), 130.44 (C-25), 129.30 (C-27), 128.95 (C-18,20), 128.68 (C-19), 128.45 (C-24), 128.28 (C-26), 126.48 (C-4), 122.46 (C-8), 120.51 (C-6), 71.50 (C-11), 63.33 (C-15), 63.30 (C-28), 57.85 (C-14), 53.56 (C-33), 53.26 (C-31), 45.68 (C-2), 31.21 (C-12), 25.89 (C-29), 23.33 (C-13).

2. *Reductive ring opening of complex **4***

Solution of Bu_4NBF_4 (10 mL 0.09 M) in DMF was placed into the two-compartment electrochemical cell equipped with the magnetic stirrer. In the working electrode compartment,

[3] S. Grimme, J. G. Brandenburg, C. Bannwarth, A. Hansen, *J. Chem. Phys.*, **2015**, 143, 054107
[4] A. V. Marenich, C. J. Cramer, D. G. Truhlar, *J. Phys. Chem. B*, **2009**, 113, 6378

complex **4** (50 mg, 0.078 mmol) and azobenzene (method A: no azobenzene; method B: 15 mg (0.082 mmol)) were added. Potentiostatic electrolysis ($E = -1.70$ V (method A), $E = -1.50$ V (method B) vs Ag/AgCl, $KCl_{(sat.)}$) of the solution deaerated with an argon flow was performed using a carbon felt as a working electrode and a Fe-rod as a counter electrode. The color of the solution was changed from red to dark purple during the electrolysis. After a charge of 7.5 C (1 F/mol of complex **4**, method A) or 18 C (2.4 F/mol of complex **4**, method B) was passed through the solution, $PhNEt_2{\cdot}HCl$ (31 mg, 0.167 mmol) was added. After 5 minutes the solution from the working electrode compartment was poured onto water (15 mL) and extracted with ethyl acetate (3 × 15 mL). Organic fractions were washed with brine, dried over Na_2SO_4 and the solvent was evaporated under reduced pressure. The residue was purified using column chromatography (hexane/acetone = 1:1). After evaporation of the solvent and drying in vacuum the following complexes were isolated:

Method A: complexes **6** (20 mg, 40%), complex **7** (20 mg, 40%).

Method B: complexes **6** (42.5 mg, 85%), complex **7** (5 mg, 10%).

Complexes **6** were characterized as a mixture of α-β alkene (**6a**) and β-γ alkene (**6b**).

Characteristic signals of the α-β alkene (**6a**):

^{1}H NMR ($CDCl_3$ δ, ppm): 5.46 (d, J = 10.0 Hz, 1H), 5.22 (d, J = 10.0 Hz, 1H), 4.31 (d, J = 12.6 Hz, 1H), 3.71 (s, 3H), 3.53 (s, 3H).

^{13}C NMR ($CDCl_3$ δ, ppm): 168.65, 168.53, 167.64, 167.18, 122.81, 48.78,

Characteristic signals of the β-γ alkene (**6b**):

^{1}H NMR ($CDCl_3$ δ, ppm): 5.06 (d, J = 7.9 Hz, 1H), 4.42 (d, J = 12.6 Hz, 1H), 3.79 (s, 3H), 3.50 (s, 3H).

^{13}C NMR ($CDCl_3$ δ, ppm): 174.24, 173.70, 164.00, 163.97.

3. *Reductive ring opening followed by the reaction with electrophiles (AcOH or MeI)*

Solution of Bu_4NBF_4 (10 mL 0.09 M) in DMF was placed into the two-compartment electrochemical cell equipped with the magnetic stirrer. In the working electrode compartment, complex **3** (60 mg, 0.1 mmol) was added. Potentiostatic electrolysis ($E = -1.70$ V vs Ag/AgCl, $KCl_{(sat.)}$) of the solution deaerated with an argon flow was performed using a carbon felt as a working electrode and a Fe-rod as a counter electrode. The color of the solution was changed from red to dark purple during the electrolysis. After a charge of 10 C (1 F/mol of complex **3**) was passed through the solution, 1 mL of DMF containing acetic acid (13 μl, 0.2 mmol) or MeI (64 μl, 1 mmol) was added to the reaction mixture. Then the reaction mixture was poured onto water (15 mL) and extracted with ethyl acetate (3 x 15 mL). Organic fractions were washed with brine, dried over Na_2SO_4 and the solvent was evaporated under reduced pressure. The residue was separated with column chromatography, using the following eluents: $CHCl_3$/AcMe = 5:1 (in the experiment with AcOH as an electrophilic additive), CCl_4/iPrOH = 10:1 (in the experiment with MeI addition). After evaporation of the solvent and drying in vacuum the following complexes were isolated:

After protonation: complex **8** (10 mg, 20%), complex **5** as a single diastereomer 1 (10 mg, 20%).

After methylation: complex **9** (25 mg, 42%), complex **5** as a single diastereomer 1 (15 mg, 25%,).

Electrolysis in the presence of AcCl allows obtaining complex **5** in the form of two diastereomers (dr = 1:1) with a total yield of 40%

Complex **5**, diastereomer 1:

HRMS (ESI): m/z 582.1534 (M+H$^+$, 582.1533 calculated for $C_{31}H_{30}N_3NiO_5$).

$[\alpha]_D^{25}$ −1670 (c = 0.035 g/100mL in MeOH)

^{1}H NMR (CDCl$_3$ δ, ppm): 8.00-7.95 (m, 2H (H-17,21)), 7.91-7.86 (m, 2H (H-8,24)), 7.51-7.47 (m, 3H (H-18,19,20)), 7.29-7.21 (m, 4H (H-5,7,25,26)), 7.14 (dd, ^{3}J = 8.2 Hz, ^{3}J = 6.9 Hz 1H (H-6)), 7.01-6.95 (m, 2H (H-23,27)), 4.26-4.15 (m, 2H (H-14,28)), 3.95 (d, ^{2}J = 12.7 Hz, 1H (H-15)), 3.70-3.53 (m, 2H (H-11,29)), 3.28 (s, 3H (H-31)), 3.07 (dd, ^{3}J = 20.3 Hz, ^{3}J = 9.7 Hz, 1H (H-29)), 3.03-2.97 (m, 1H (H-14)), 2.95 (d, ^{2}J = 12.7 Hz, 1H (H-15)), 2.27-2.18 (m, 1H (H-13)), 2.04-1.86 (m, 3H (H-12,12,13)).

^{13}C NMR (CDCl$_3$ δ, ppm): 179.04 (C-10), 177.03 (C-2), 169.31 (C-30), 166.75 (C-1), 142.12 (C-22), 136.97 (C-9), 133.58 (C-16), 131.43 (C-17,21), 131.26 (C-4), 129.34, 129.31 (C-18,19,20), 128.77, 128.72, 128.66 (C-5,7,25,26), 127.48 (C-24), 126.54 (C-8), 126.22 (C-23,27), 122.76 (C-6), 79.36 (C-3), 68.32 (C-11), 59.60 (C-15), 57.94 (C-14), 53.72 (C-28), 52.53 (C-31), 36.22 (C-29), 27.43 (C-13), 22.20 (C-12).

Complex **5**, diastereomer 2:

^{1}H NMR (CDCl$_3$ δ, ppm): 8.25-8.21 (m, 2H (H-17,21)), 7.75-7.70 (m, 2H (H-23,27)), 7.46-7.26 (m, 7H (H-8,18,19,20,24,25,26)), 7.15 (dd, ^{3}J = 7.7 Hz, ^{4}J = 1.6 Hz, 1H (H-5)), 6.98-6.93 (m, 1H (H-7)), 6.88 (td, J = 7.5 Hz, J = 1.4 Hz, 1H (H-6)), 4.30 (d, ^{2}J = 12.5 Hz, 1H (H-15)), 3.88 (dd, ^{3}J = 5.9 Hz, ^{3}J = 1.5 Hz, 1H (H-28)), 3.80-3.66 (m, 1H (H-13)), 3.58 (s, 3H (H-31)), 3.57-3.46 (m, 2H (H-14,15)), 3.33 (dd, ^{3}J = 10.4 Hz, ^{3}J = 6.2 Hz, 1H (H-11)), 2.88 (dd, ^{2}J = 19.1 Hz, ^{3}J = 1.5 Hz, 1H (H-29)), 2.82 (dd, ^{2}J = 19.1 Hz, ^{3}J = 5.9 Hz, 1H (H-29)), 2.50-2.34 (m, 2H (H-12)), 2.33-2.23 (m, 1H (H-13)), 2.23-2.13 (m, 1H (H-14)).

^{13}C NMR (CDCl$_3$ δ, ppm): 179.34 (C-10), 178.59 (C-2), 171.82 (C-30), 166.69 (C-1), 139.46 (C-22), 139.37 (C-9), 133.66 (C-16), 131.54 (C-17,21), 130.42 (C-4), 129.38 (C-19), 129.33 (C-18,20), 129.19 (C-24,26), 128.46 (C-25), 127.37 (C-7), 126.74 (C-8), 125.44 (C-5,23,27), 122.29 (C-6), 77.80 (C-3), 71.75 (C-11), 63.36 (C-15), 57.84 (C-14), 56.52 (C-28), 52.56 (C-31), 36.96 (C-29), 30.55 (C-12), 24.66 (C-13).

Complex **8**:

HRMS (ESI): m/z 582.1541 (M+H$^+$, 582.1533 calculated for $C_{31}H_{30}N_3NiO_5$).

The compound was obtained as an inseparable mixture of stereo- and regioisomeric alkenes with β-γ trans-isomer as a main component. The characteristic signals of the latter are listed below: ^{1}H NMR (CDCl$_3$ δ, ppm) (characteristic signals): 7.02 (dd, J = 15.7, 5.7 Hz, 1H), 6.25 (dd, J = 15.7, 1.6 Hz, 1H), 4.62 (dd, J = 5.1, 1.6 Hz, 1H), 3.77 (s, 3H).

Methylated complex **9**:

HRMS (ESI): m/z 596.1690 (M+H$^+$, 596.1690 calculated for $C_{32}H_{32}N_3NiO_5$).

^{1}H NMR (CDCl$_3$ δ, ppm): 8.12-8.07 (m, 3H (H-8,17,21)), 7.47-7.39 (m, 3H (H-23,24,26)), 7.34-7.29 (m, 2H (H-18,20)), 7.18-7.10 (m, 4H (H-7,19,25,27)), 6.89 (dd, ^{3}J = 8.3 Hz, ^{4}J = 1.6 Hz, 1H (H-5)), 6.70 (ddd, ^{3}J = 8.3 Hz, ^{3}J = 7.0 Hz, ^{4}J = 1.2 Hz, 1H (H-6)), 5.02 (d, ^{3}J = 10.3 Hz, 1H (H-28)), 4.36-4.27 (m, 2H (H-15,29)), 3.90-3.77 (m, 1H (H-13)), 3.64 (s, 3H (H-31)), 3.48-3.43

(m, 1H (H-11)), 3.40 (d, ^{3}J = 12.6 Hz, 1H (H-15)), 2.78-2.69 (m, 1H (H-12)), 2.63-2.45 (m, 2H (H-12,14)), 2.25-2.16 (m, 1H (H-13)), 2.11-2.03 (m, 1H (H-14)), 0.74 (d, ^{3}J = 7.1 Hz, 3H (H-32)).

^{13}C NMR ($CDCl_3$ δ, ppm): 180.25 (C-10), 174.26 (C-30), 168.97 (C-1), 167.71 (C-3), 143.46 (C-9), 140.74 (C-2), 135.08 (C-22), 134.09 (C-5), 133.65 (C-16), 132.95 (C-7), 131.49 (C-17,21), 131.35 (C-28), 129.85 (C-24,26), 129.15 (C-25), 129.11 (C-27), 129.07 (C-18,20), 128.97 (C-19), 128.87 (C-23), 127.02 (C-4), 123.67 (C-8), 120.83 (C-6), 70.75 (C-11), 63.04 (C-15), 57.53 (C-14), 52.14 (C-31), 36.16 (C-29), 30.82 (C-12), 24.14 (C-13), 17.28 (C-32).

4. *One-pot electrosynthesis of cysteine derivatives from complex 4*

Solution of Bu_4NBF_4 (10 mL 0.09 M) in DMF was placed into the two-compartment electrochemical cell equipped with the magnetic stirrer. In the working electrode compartment, complex **4** (50 mg, 0.078 mmol) and azobenzene (15 mg, 0.082 mmol) were added. Potentiostatic electrolysis (E = −1.50 V vs Ag/AgCl, $KCl_{(sat.)}$) of solution deaerated with an argon flow was performed using carbon felt as a working electrode and Fe-rod as a counter electrode. The color of the solution during the electrolysis changed from red to dark purple. After a charge of 18 C (2.4 F/mol of complex **4**) was passed through the solution, $PhNEt_2$·HCl (31 mg, 0.167 mmol) was added. After 5 min of intensive stirring, RSH (0.16 mmol) and Et_3N (method B) were added (method A: no Et_3N; method B: 7.26 mg, 0.07 mmol of Et_3N). The solution from the working electrode compartment was transferred to the Schlenk tube preliminary charged with argon and kept at room temperature for 24 h. Then the reaction mixture was poured onto water (15 mL) and extracted with ethyl acetate (3 × 15 mL). Organic fractions were washed with brine, dried over Na_2SO_4 and the solvent was evaporated under reduced pressure. The residue was separated using column chromatography (Silicagel, $CHCl_3$/AcMe = 15:1 mixture was used as an eluent; for further purification of each diastereomer hexane/AcOEt = 1:5 mixture was used). After evaporation of the solvent and drying in vacuum the following complexes were obtained:

Method A:

TolSH: complex **10** (32 mg, 54%, (*R*,*S*)/(*R*,*R*)=1:5), complexes **6** (20 mg (40%)).

BnSH: complex **12** (38 mg, 64%, (*R*,*S*)/(*R*,*R*)=1:2.6), complexes **6** (10 mg (20%)).

Method B:

TolSH: complex **10** (38 mg, 64%, (*R*,*S*)/(*R*,*R*)=10:1), complexes **6** (8 mg (16%)).

PhSH: complex **11** (47 mg, 88%, (*R*,*S*)/(*R*,*R*)=12:1).

BnSH: complex **12** (25 mg, 42%, pure (*R*,*S*)-diastereomer), complexes **6** (24 mg (48%)) (40°C, 72 hours)

(*R*,*S*)-**10**

$[\alpha]_D^{25}$ +1410 (c = 0.03 g/100mL in MeOH)

HRMS (ESI): m/z 764.1954 (M+H$^+$, 764.1935 calculated for $C_{40}H_{40}N_3NiO_7S$), 786.1774 (M+Na$^+$, 786.1754 calculated for $C_{40}H_{39}N_3NaNiO_7S$).

^{1}H NMR ($CDCl_3$ δ, ppm): 8.37 (dd, ^{3}J = 8.8 Hz, ^{4}J = 1.1 Hz, 1H (H-8)), 8.10-8.05 (m, 2H (H-17,21)), 7.53-7.43 (m, 4H (H-25,26,35,39)), 7.36-7.30 (m, 2H (H-18,20)), 7.25-7.11 (m, 4H (H-7,19,24,27), 6.84-6.80 (m, 2H (H-36,38)), 6.57 (ddd, ^{3}J = 8.3 Hz, ^{3}J = 7.0 Hz, ^{4}J = 1.1 Hz, 1H (H-6)), 6.41 (dd, ^{3}J = 8.3 Hz, ^{4}J = 1.6 Hz, 1H (H-5)), 5.66-5.62 (m, 1H (H-23)), 4.61 (d, ^{3}J = 5.6 Hz, 1H (H-2)), 4.48 (d, ^{2}J = 12.6 Hz, 1H (H-15)), 4.06 (d, ^{3}J = 11.4 Hz, 1H (H-29)), 3.89-

3.78 (m, 1H (H-13)), 3.74 (s, 3H (H-31)), 3.71-3.64 (m, 1H (H-14)), 3.61 (d, ^{2}J = 12.6 Hz, 1H (H-15)), 3.52-3.48 (m, 4H (H-11,33)), 3.41 (dd, ^{3}J = 11.4 Hz, ^{3}J = 5.6 Hz, 1H (H-28)), 2.88-2.78 (m, 1H (H-12)), 2.54-2.41 (m, 1H (H-12)), 2.12 (s, 3H (H-40)), 2.15-2.01 (m, 2H (H-13,14)).

^{13}C NMR ($CDCl_3$ δ, ppm): 180.58 (C-10), 176.57 (C-1), 172.40 (C-3), 168.56 (C-30), 166.63 (C-32), 143.01 (C-9), 138.83 (C-37), 134.28 (C-35,39), 133.71 (C-22), 133.63 (C-5), 133.53 (C-16), 132.44 (C-7), 131.76 (C-17,21), 131.20 (C-34), 129.91 (C-36,38), 129.81 (C-25), 129.07 (C-26), 128.90 (C-19), 128.84 (C-18,20), 128.66 (C-24), 127.37 (C-23), 127.06 (C-27), 125.88 (C-4), 123.58 (C-8), 120.43 (C-6), 70.67 (C-11), 70.06 (C-2), 63.55 (C-15), 57.40 (C-14), 55.26 (C-29), 52.83 (C-31), 52.71 (C-33), 52.24 (C-28), 30.75 (C-12), 23.44 (C-13), 21.03 (C-40).

(*R*,*R*)-**10**

$[\alpha]_D^{25}$ +1165 (c = 0.031 g/100mL in MeOH)

^{1}H NMR ($CDCl_3$ δ, ppm): 8.38 (dd, ^{3}J = 8.8 Hz, ^{4}J = 1.1 Hz, 1H (H-8)), 8.01-7.96 (m, 2H (H-17,21)), 7.54-7.43 (m, 4H (H-24,25,26,27)), 7.28-7.17 (m, 5H (H-18,20,23,35,39)), 7.14 (ddd, ^{3}J = 8.8 Hz, ^{3}J = 6.9 Hz, ^{4}J = 1.7 Hz, 1H (H-7)), 7.10-7.05 (m, 1H (H-19)), 6.99-6.95 (m, 2H (H-36,38)), 6.77 (dd, ^{3}J = 8.3 Hz, ^{4}J = 1.7 Hz, 1H (H-5)), 6.69 (ddd, ^{3}J = 8.3 Hz, ^{3}J = 6.9 Hz, ^{4}J = 1.1 Hz, 1H (H-6)), 4.78 (dd, ^{3}J = 9.7 Hz, ^{3}J = 4.4 Hz, 1H (H-28)), 4.47 (d, ^{3}J = 4.4 Hz, 1H (H-29)), 4.28 (d, ^{2}J = 12.6 Hz, 1H (H-15)), 4.13 (d, ^{3}J = 9.7 Hz, 1H (H-2)), 3.72 (s, 3H (H-31)), 3.78 (s, 3H (H-33)), 3.38 (d, ^{2}J = 12.6 Hz, 1H (H-15)), 3.35-3.28 (m, 2H (H-11,14)), 3.23-3.07 (m, 1H (H-13)), 2.28 (s, 3H (H-40)), 2.26-2.16 (m, 1H (H-12)), 2.15-2.06 (m, 1H (H-12)), 2.01-1.90 (m, 2H (H-13,14)).

^{13}C NMR ($CDCl_3$ δ, ppm): 179.65 (C-10), 176.80 (C-1), 173.75 (C-3), 168.48 (C-30), 167.29 (C-32), 143.28 (C-9), 138.58 (C-37), 134.61 (C-5), 134.41 (C-22), 133.63 (C-35,39), 133.55 (C-16), 133.07 (C-7), 132.43 (C-34), 131.41 (C-17,21), 130.17 (C-36,38), 128.91 (C-18,20), 128.78 (C-19), 131.78, 129.67,128.69, 128.56, 127.87 (C-23,24,25,26,27), 126.06 (C-4), 122.95 (C-8), 120.57 (C-6), 73.12 (C-2), 70.43 (C-11), 63.19 (C-15), 57.92 (C-28), 57.33 (C-14), 54.26 (C-29), 53.07 (C-31), 52.46 (C-33), 29.94 (C-12), 23.77 (C-13), 21.24 (C-40).

(*R*,*S*)-**11**

$[\alpha]_D^{25}$ +1193 (c = 0.03 g/100mL in MeOH)

^{1}H NMR ($CDCl_3$ δ, ppm): 8.37 (dd, ^{3}J = 8.7 Hz, ^{4}J = 1.1 Hz, 1H), 8.10-8.05 (m, 2H), 7.68-7.63 (m, 2H), 7.53-7.43 (m, 2H), 7.35-7.30 (m, 2H), 7.24-7.05 (m, 5H), 7.04-6.99 (m, 2H), 6.56 (ddd, ^{3}J = 8.2 Hz, ^{3}J = 7.0 Hz, ^{4}J = 1.1 Hz, 1H), 6.38 (dd, ^{3}J = 8.3 Hz, ^{4}J = 1.6 Hz, 1H), 5.52-5.47 (m, 1H), 4.63 (d, ^{3}J = 5.7 Hz, 1H), 4.48 (d, ^{2}J = 12.6 Hz, 1H), 4.08 (d, ^{3}J = 11.4 Hz, 1H), 3.94-3.80 (m, 1H), 3.75 (s, 3H), 3.71-3.64 (m, 1H), 3.62 (d, ^{2}J = 12.6 Hz, 1H), 3.54-3.48 (m, 4H), dd (dd, ^{3}J = 11.4 Hz, ^{3}J = 5.7 Hz, 1H), 2.91-2.81 (m, 1H), 2.57-2.44 (m, 1H), 2.17-2.03 (m, 2H).

^{13}C NMR ($CDCl_3$ δ, ppm): 180.55, 176.58, 172.45, 168.66, 166.64, 143.03, 134.76, 134.29, 133.65, 133.53, 132.48, 131.76, 129.88, 129.22, 129.08, 128.92, 128.87, 128.63, 128.60, 127.37, 127.07, 125.82, 123.55, 120.46, 70.65, 70.01, 63.58, 57.38, 55.23, 52.90, 52.78, 52.08, 30.74, 23.48.

(*R*,*S*)-**12**

$[\alpha]_D^{25}$ +1159 (c = 0.033 g/100mL in MeOH)

^{1}H NMR ($CDCl_3$ δ, ppm): 8.46 (dd, ^{3}J = 8.7 Hz, ^{4}J = 1.0 Hz, 1H), 8.10-8.03 (m, 2H), 7.57-7.48 (m, 2H), 7.44-7.37 (m, 1H), 7.34-7.10 (m, 7H), 7.06-6.97 (m, 3H), 6.65 (ddd, ^{3}J = 8.3 Hz,

^3J = 7.0 Hz, ^4J = 1.0 Hz, 1H), 6.52 (dd, ^3J = 8.3 Hz, ^4J = 1.5 Hz, 1H), 6.30-6.24 (m, 1H), 4.46-4.37 (m, 2H), 4.15 (d, ^3J = 11.4 Hz), 4.00 (d, J = 11.7 Hz), 3.80 (s, 3H), 3.60-3.51 (m, 2H), 3.44 (s, 3H), 3.41-3.29 (m, 1H), 3.18 (dd, ^3J = 11.4, ^3J = 4.9 Hz, 1H), 2.68-2.56 (m, 1H), 2.37-2.24 (m, 1H), 2.10-1.99 (m, 1H), 1.88-1.77 (m, 1H), 1.71-1.58 (m, 1H), 1.29-1.22 (m, 1H).

^{13}C NMR ($CDCl_3$ δ, ppm): 180.43, 176.15, 171.95, 168.41, 166.57, 143.50, 136.39, 133.93, 133.67, 132.69, 131.73, 129.89, 129.64, 129.26, 129.10, 128.83, 128.81, 127.87, 127.60, 127.22, 125.98, 123.35, 120.48, 70.85, 69.89, 63.82, 57.36, 55.00, 52.95, 52.73, 46.81, 40.79, 30.43, 23.19.

(*R*,*R*)-**12**

$[\alpha]_D^{25}$ +1279 (c = 0.027 g/100mL in MeOH)

^1H NMR ($CDCl_3$ δ, ppm): 8.43 (d, ^3J = 8.8 Hz, 1H), 8.09-8.04 (m, 2H), 7.54-7.44 (m, 2H), 7.43-7.37 (m, 1H), 7.32-7.23 (m, H), 7.19-7.07 (m, 5H), 6.99-6.94 (m, 2H), 6.75-6.66 (m, 2H), 4.52-4.38 (m, 3H), 4.19 (d, J = 7.9 Hz), 4.01 (d, J = 10.6 Hz, 1H), 3.93 (d, J = 10.6 Hz, 1H), 3.70 (s, 3H), 3.58-3.43 (m, 7H), 2.75-2.65 (m, 1H), 2.56-2.44 (m, 1H), 2.17-2.06 (m, 2H).

^{13}C NMR ($CDCl_3$ δ, ppm): 180.30, 176.74, 173.76, 168.52, 167.33, 143.09, 136.44, 134.50, 134.37, 133.59, 133.16, 131.49, 131.27, 129.81, 129.20, 128.99, 128.87, 128.84, 128.68, 128.56, 127.84, 127.37, 125.97, 123.04, 120.76, 72.95, 70.82, 63.62, 57.54, 54.39, 53.27, 52.55, 51.52, 41.31, 30.66, 23.91.

5. *Semi-integral voltammogram for complex **2** (100 mV/s)*

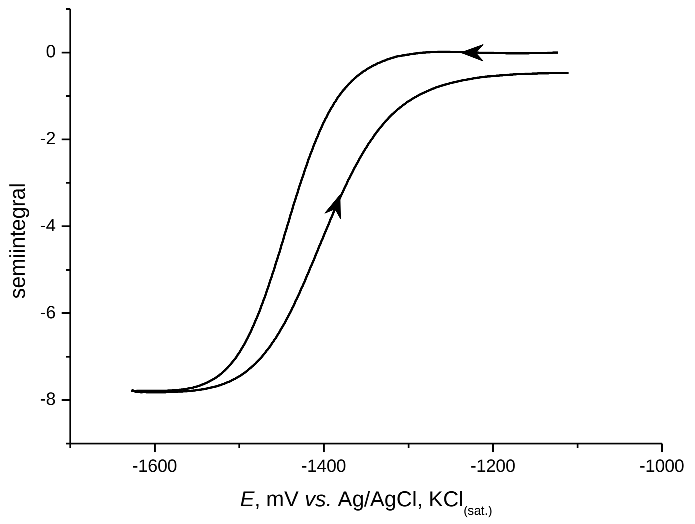

6. *Semi-differential voltammograms for complex **4** at various scan rates*

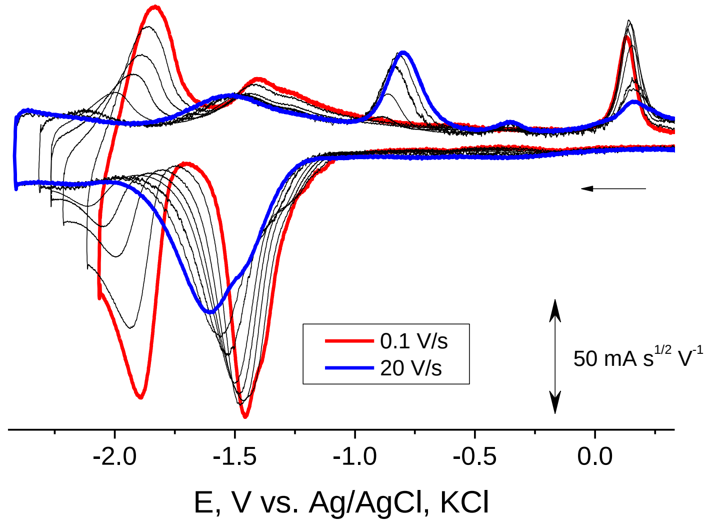

7. *Determination of the pK$_a$ of complex* ***6***

To determine pK_a of complex **6** (designated as *HA* in the following text) an equilibrium between *HA* as an acid and pyridine as a base in DMSO was used.

$$HA + Py \rightleftarrows A^- + PyH^+$$

$$K = \frac{[PyH^+][A^-]}{[HA][Py]} = \frac{K_a(HA)}{K_a(PyH^+)} \qquad (1)$$

Assuming no solvent protonation, $[PyH^+] = [A^-]$. Thus, an expression for acidity constant is:

$$K_a(HA) = K_a(PyH^+)\frac{[A^-]^2}{[HA][Py]} \qquad (2)$$

$pK_a(PyH^+) = 3.4$ [5]

The following procedure was applied to measure equilibrium concentrations in eq. 2. A pyridine solution in DMSO (23.5 mM) was added portionwise to a solution of *HA* (2 mL, $2.35\cdot10^{-4}$ M) in DMSO. UV-vis spectra were recorded for the initial solution and after addition of each pyridine portion. Finally, an excess of sodium methylate was added to ensure full deprotonation of *HA*. New intensive peak (at 519 nm) corresponding to absorption of the anion A^- appeared. Absorption of the solution at this wavelength (*A*) was used for pK$_a$ measurement.

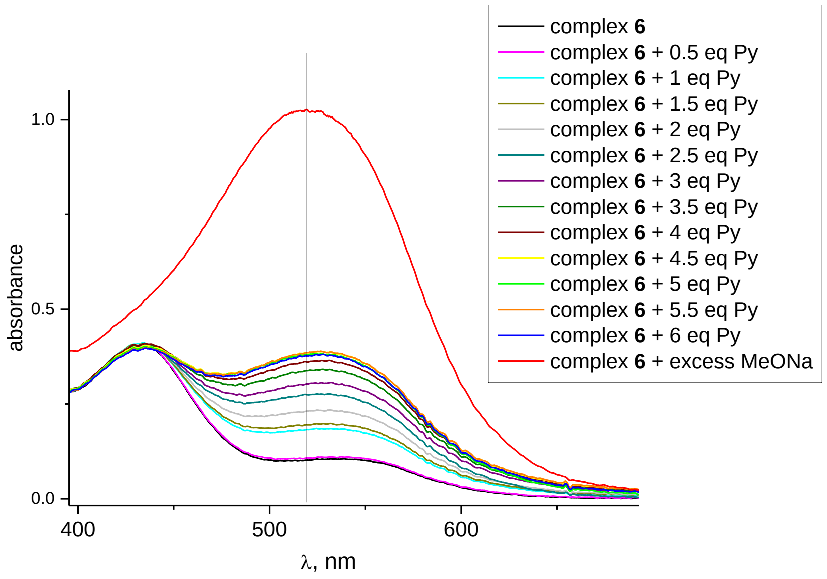

First, extinction coefficients at 519 nm was determined for both *HA* and A^- from the spectra of the initial solution and the solution of completely deprotonated complex. The $\varepsilon_{HA} = 438\ l\ mol^{-1}cm^{-1}$ and $\varepsilon_{A^-} = 4673\ l\ mol^{-1}cm^{-1}$ values were obtained and used to determine the equilibrium concentrations of *HA*, A^- and pyridine in solution.

HA and A^-contribute to the 519 nm absorption band:

[5] Izaak M. Kolthoff, Miran K. Chantooni Jr., Sadhana Bhowmik, J. Am. Chem. Soc. **1968**, 90, 1, 23–28.

$$A = l\left(\varepsilon_{HA}[HA] + \varepsilon_{A^-}[A^-]\right) \qquad (3)$$

$$[A^-] + [HA] = c_0 \frac{V_0}{V_0 + V_{Py}} \qquad (4)$$

Combining (3) and (4) yields an expression for [*HA*]:

$$[HA] = \left(\varepsilon_{A^-} c_0 \frac{V_0}{V_0 + V_{Py}} - \frac{A}{l}\right)\left(\varepsilon_{A^-} - \varepsilon_{HA}\right)^{-1}$$

$$[A^-] = c_0 \frac{V_0}{V_0 + V_{Py}} - [HA]$$

$$[Py] = c_{Py} \frac{V_{Py}}{V_0 + V_{Py}} - [PyH^+] = c_{Py} \frac{V_{Py}}{V_0 + V_{Py}} - [A^-]$$

The data are collected in Table 1.

Volume of the pyridine solution added, V_{Py}, mL	Absorption at 519 nm	[*HA*], M	[A^-], M	[*Py*], M	K_a	pK_a
0	0.103	2.35E-04	0.00E+00	0.00E+00		
0.01	0.107	2.33E-04	1.07E-06	1.16E-04	1.68E-08	7.8
0.02	0.181	2.14E-04	1.87E-05	2.14E-04	3.03E-06	5.5
0.03	0.197	2.09E-04	2.26E-05	3.25E-04	2.99E-06	5.5
0.04	0.232	1.99E-04	3.09E-05	4.30E-04	4.44E-06	5.4
0.05	0.275	1.88E-04	4.12E-05	5.32E-04	6.76E-06	5.2
0.06	0.303	1.80E-04	4.79E-05	6.37E-04	7.97E-06	5.1
0.07	0.337	1.71E-04	5.61E-05	7.39E-04	9.91E-06	5.0
0.08	0.362	1.64E-04	6.21E-05	8.42E-04	1.11E-05	5.0
0.09	0.385	1.57E-04	6.76E-05	9.44E-04	1.23E-05	4.9
0.1	0.38	1.57E-04	6.66E-05	1.05E-03	1.07E-05	5.0
0.11	0.386	1.55E-04	6.81E-05	1.16E-03	1.03E-05	5.0
0.12	0.377	1.56E-04	6.61E-05	1.26E-03	8.84E-06	5.1
Excess MeONa	1.036	0	2.22E-04			

Averaging all the measured values (except 7.8) yields the final pK_a value of complex **6**:

pK_a (complex **6**) = 5.1

*8. Atom numeration and signal assignment in the NMR spectra of complex (S)-**4***

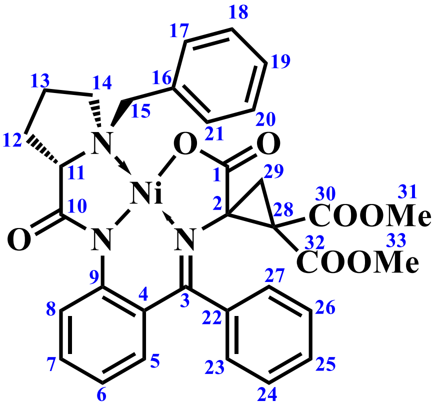

^{1}H NMR ($CDCl_3$ δ, ppm): 8.21 (dd, ^3J = 8.7 Hz, ^4J = 0.9 Hz, 1H (H-8)), 8.16-8.11 (m, 2H (H-17,21)), 7.54-7.48 (m, 1H (H-25)), 7.47-7.40 (m, 2H (H-24,26)), 7.26-7.13 (m, 4H (H-18,20,23,27)), 7.10-7.00 (m, 2H (H-7,19)), 6.83 (dd, ^3J = 8.3 Hz, ^4J = 1.6 Hz, 1H (H-5)), 6.69-6.64 (m, 1H (H-6)), 4.33-4.17 (m, 2H (H-13,15)), 3.77 (s, 3H (H-31)), 3.72 (s, 3H (H-33)), 3.60-3.53 (m, 1H (H-14)), 3.44 (dd, ^3J = 11.0 Hz, ^3J = 5.8 Hz, 1H (H-11)), 3.31 (d, ^2J = 12.5 Hz, 1H (H-15)), 2.79-2.68 (m, 1H (H-12)), 2.64-2.51 (m, 1H (H-12)), 2.30-2.19 (m, 1H (H-13)), 2.16-2.06 (m, 1H (H-14)), 1.90 (d, ^2J = 7.6 Hz, 1H (H-29)), 0.93 (d, ^2J = 7.6 Hz, 1H (H-29)).

^{13}C NMR ($CDCl_3$ δ, ppm): 180.31 (C-10), 172.49 (C-3), 171.54 (C-1), 165.90 (C-30), 165.54 (C-32), 143.73 (C-9), 134.84 (C-22), 134.36 (C-5), 133.97 (C-16), 133.25 (C-7), 131.21 (C-17,21), 130.55 (C-23), 130.44 (C-25), 129.30 (C-27), 128.95 (C-18,20), 128.68 (C-19), 128.45 (C-24), 128.28 (C-26), 126.48 (C-4), 122.46 (C-8), 120.51 (C-6), 71.50 (C-11), 63.33 (C-15), 63.30 (C-28), 57.85 (C-14), 53.56 (C-33), 53.26 (C-31), 45.68 (C-2), 31.21 (C-12), 25.89 (C-29), 23.33 (C-13).

*9. ^{1}H NMR spectrum of complex (S)-**4***

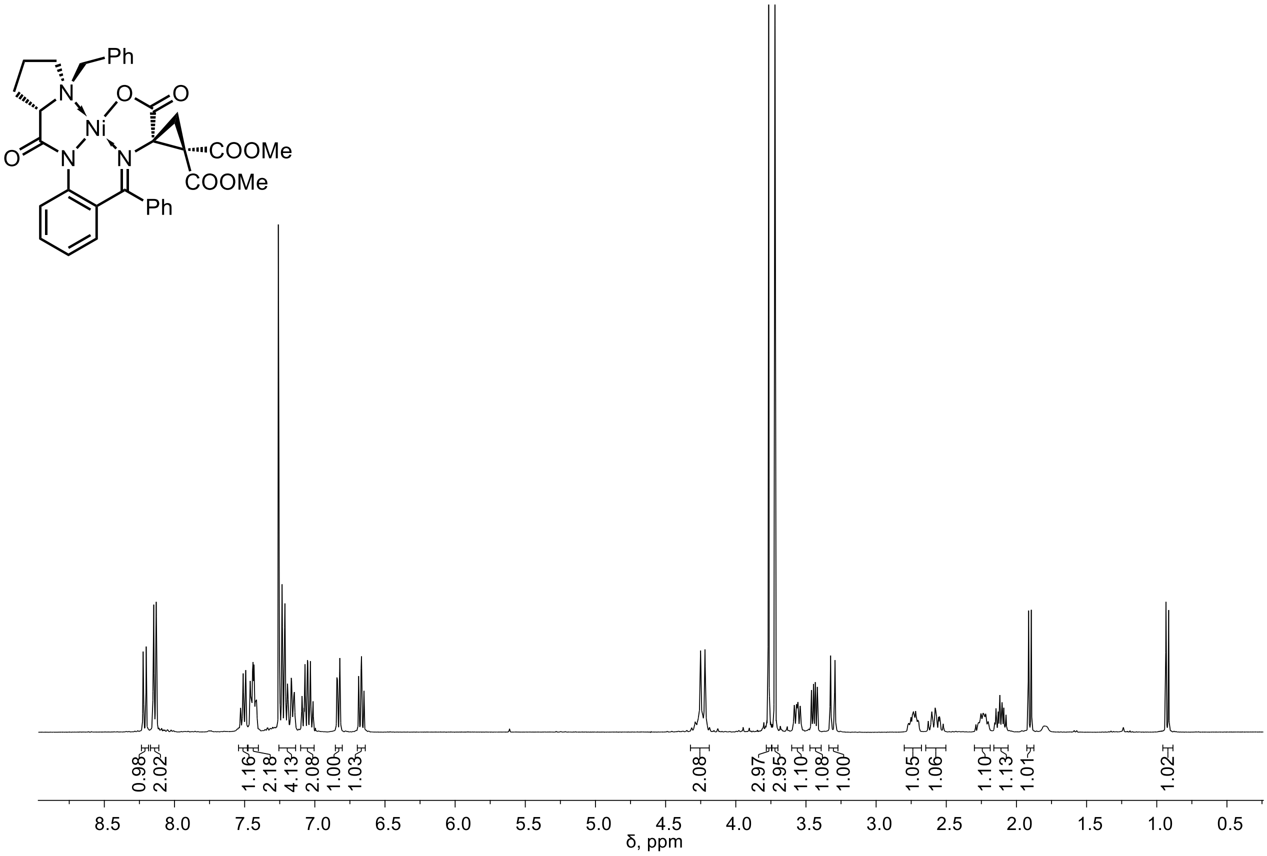

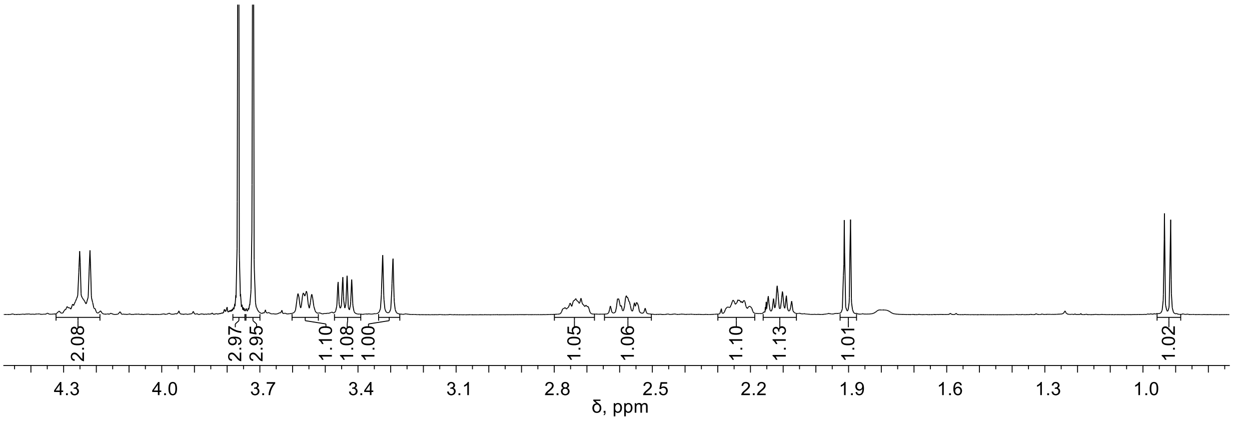

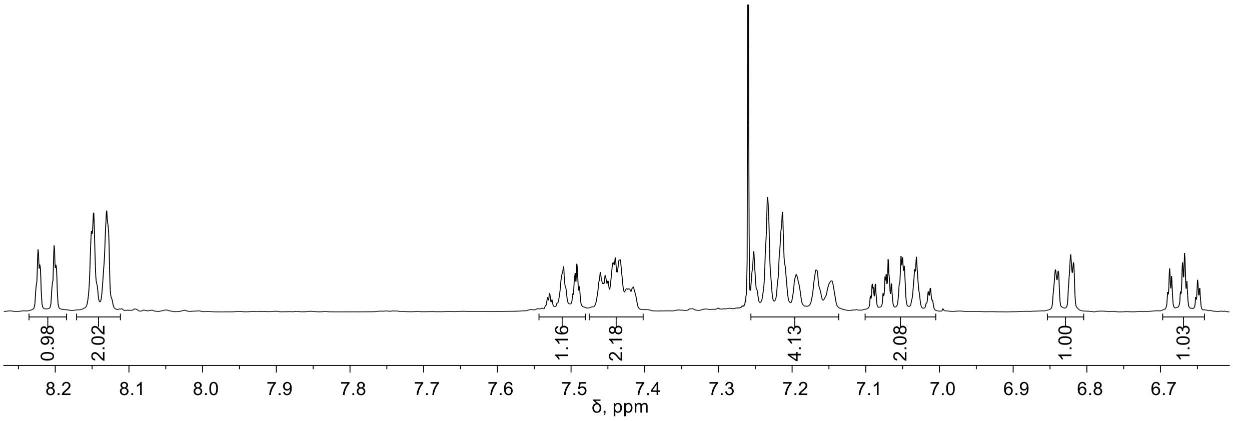

*10. ^{13}C NMR spectrum of complex (S)-**4***

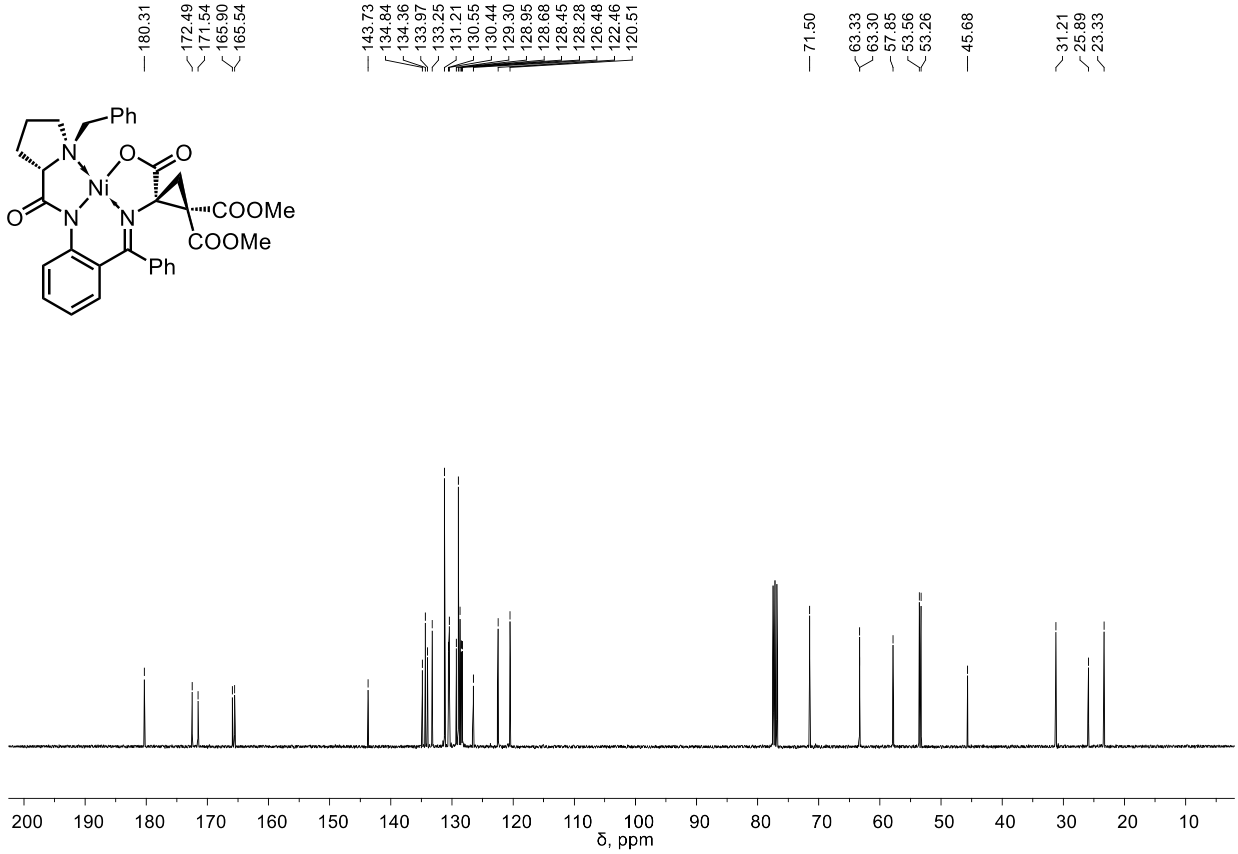

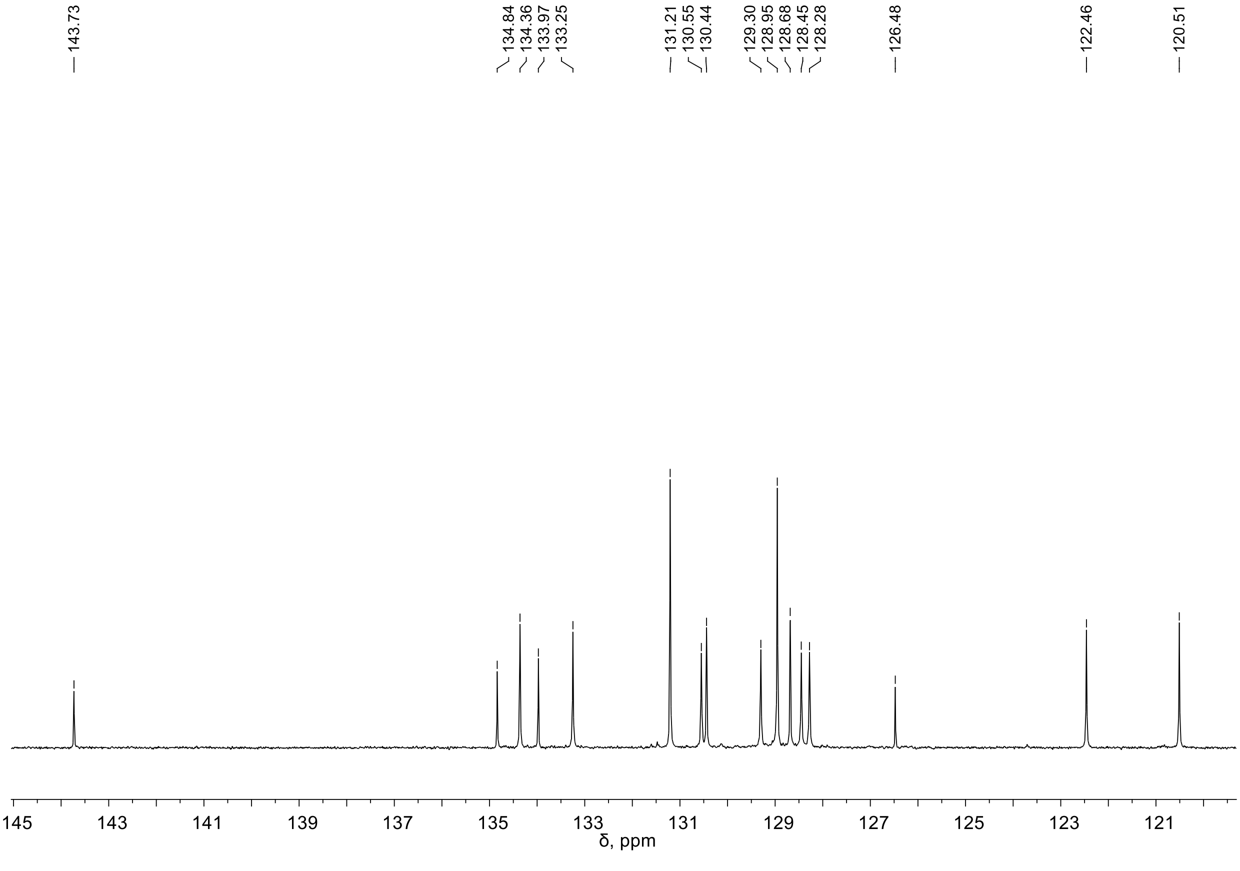

*11. COSY spectrum of complex (S)-**4***

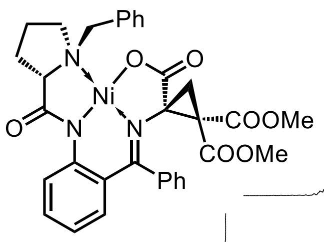

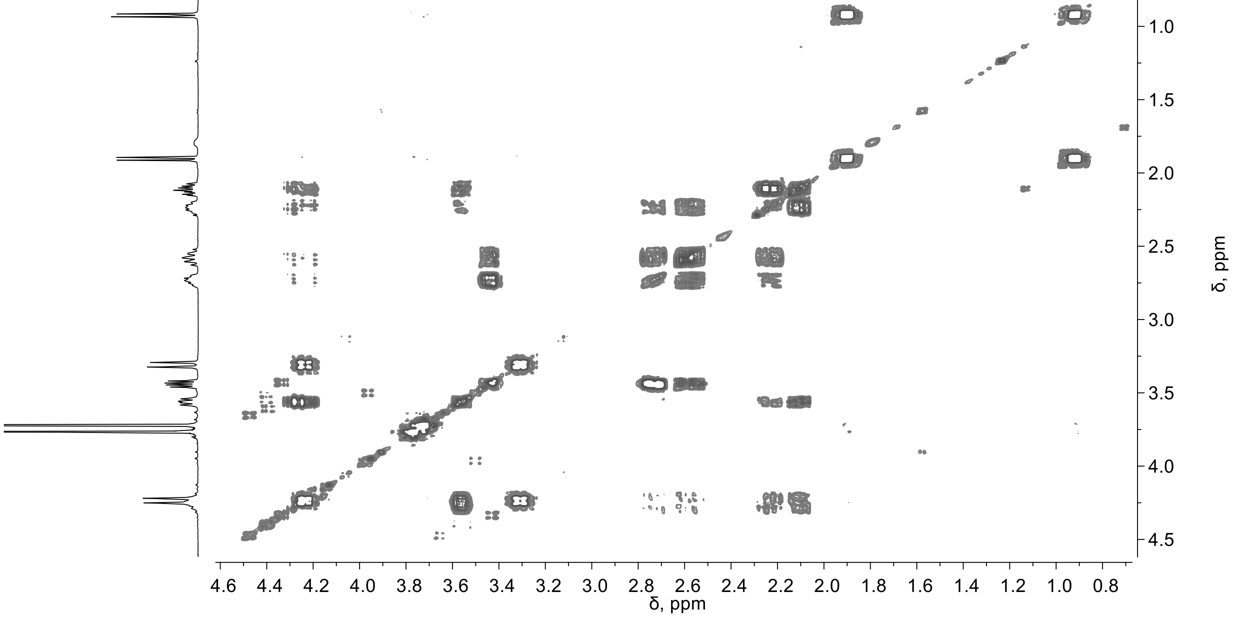

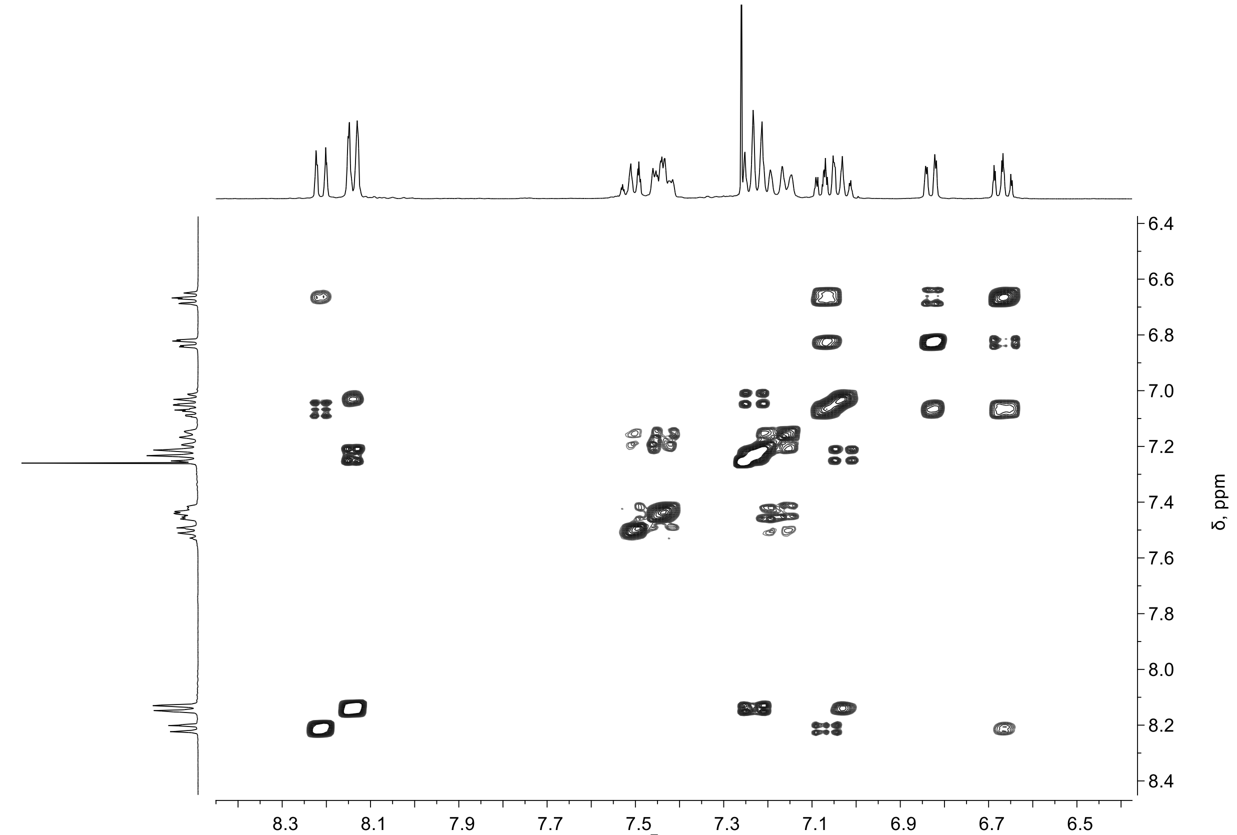

*12. HSQC spectrum of complex (S)-**4***

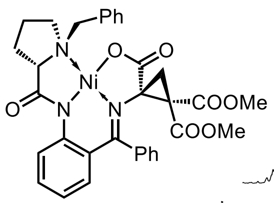

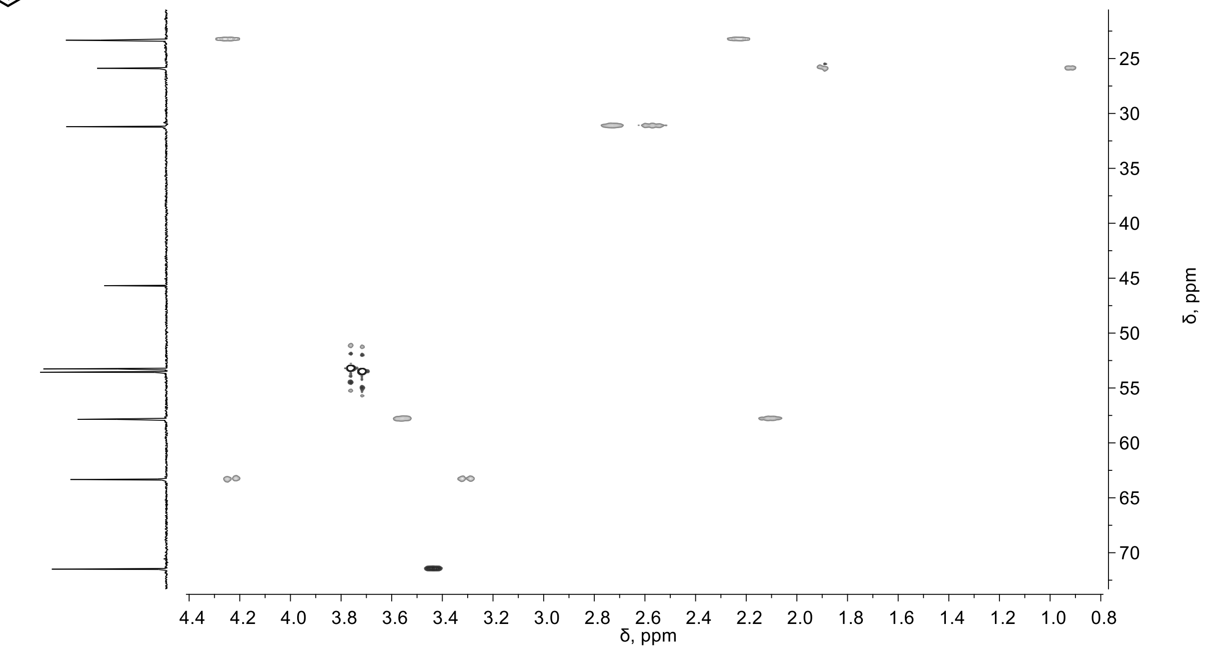

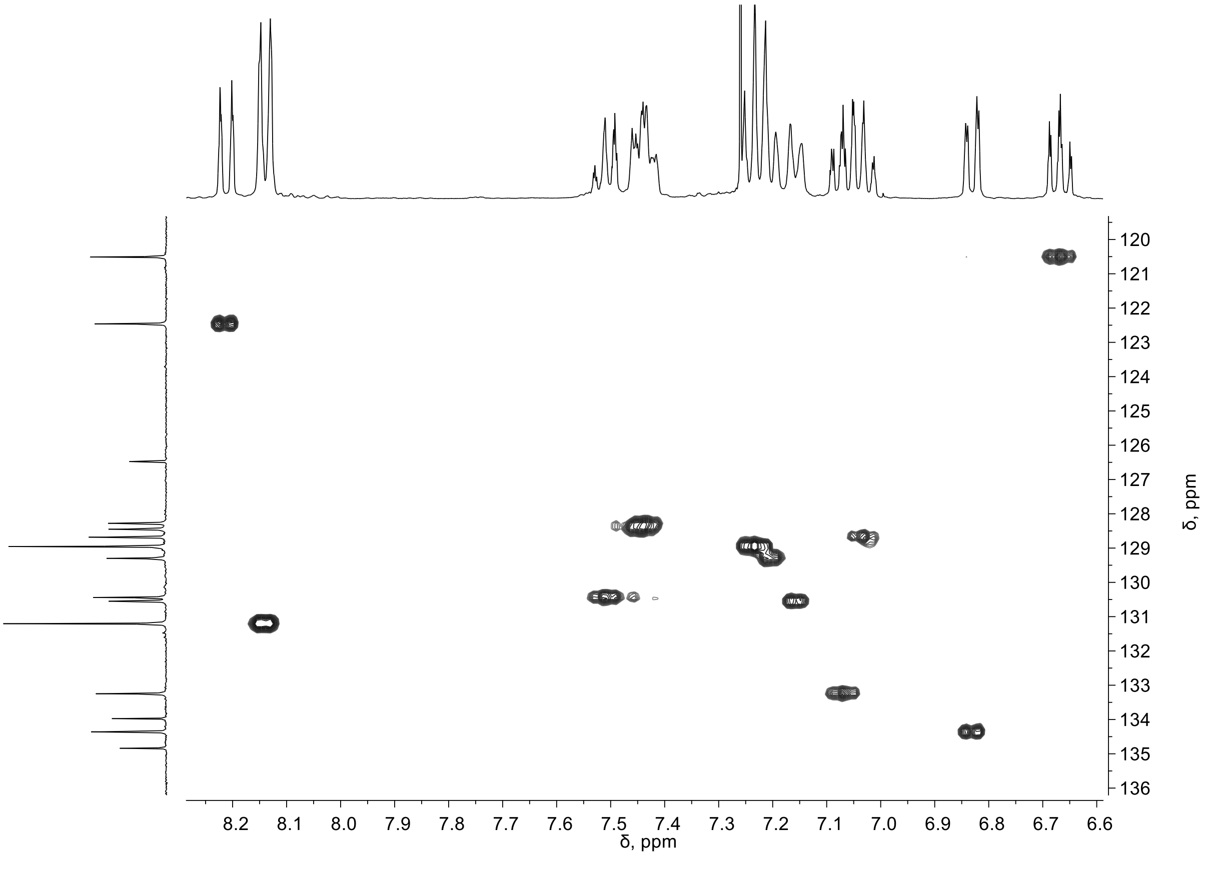

*13. HMBC spectrum of complex (S)-**4***

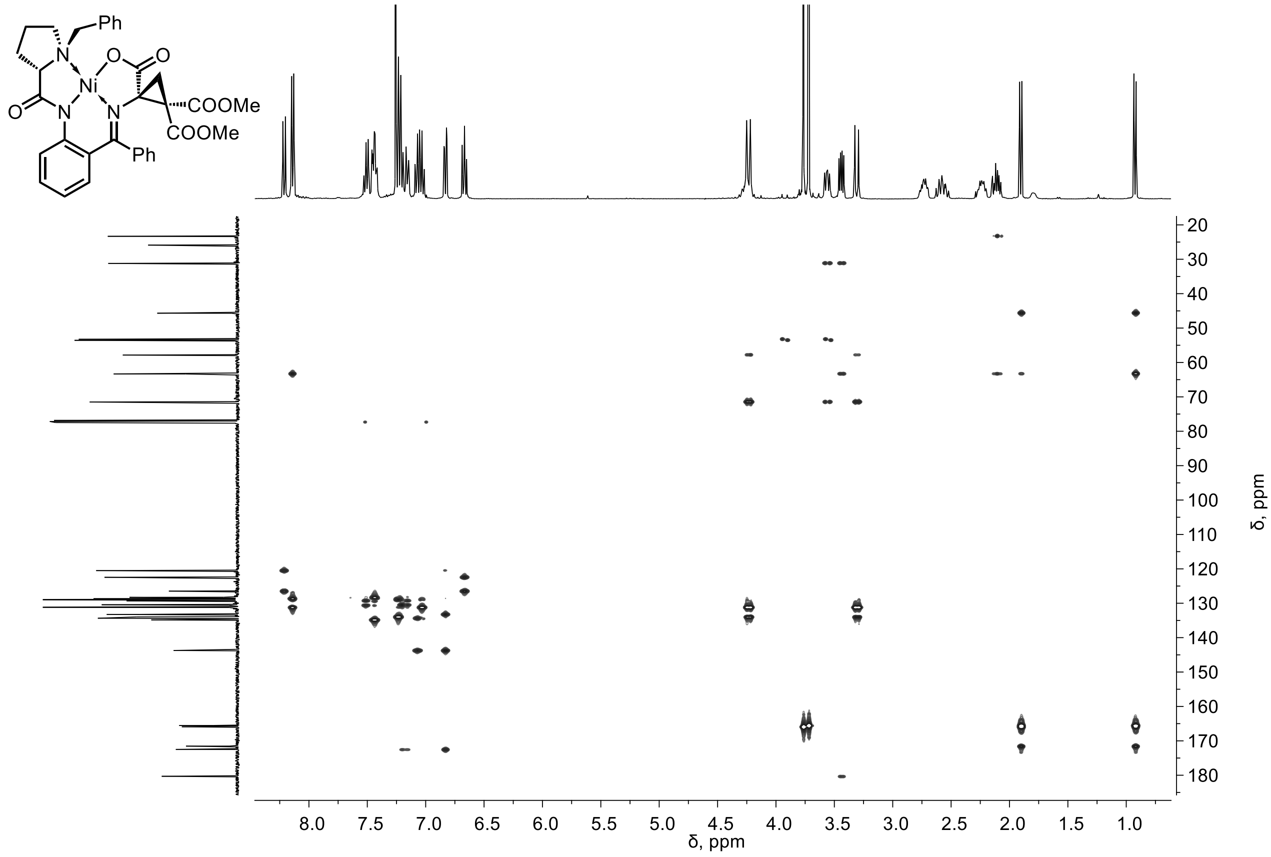

*14. NOESY spectrum of complex (S)-**4***

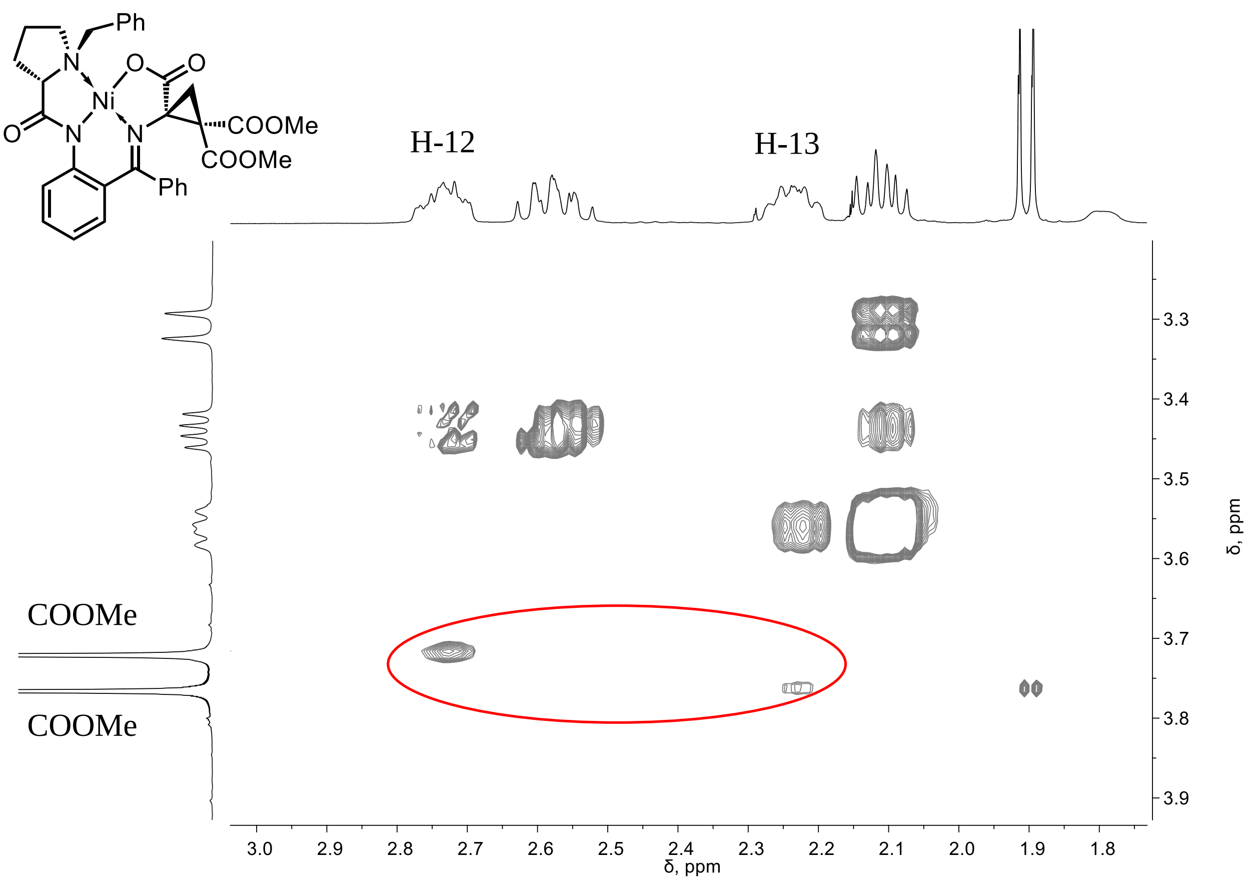

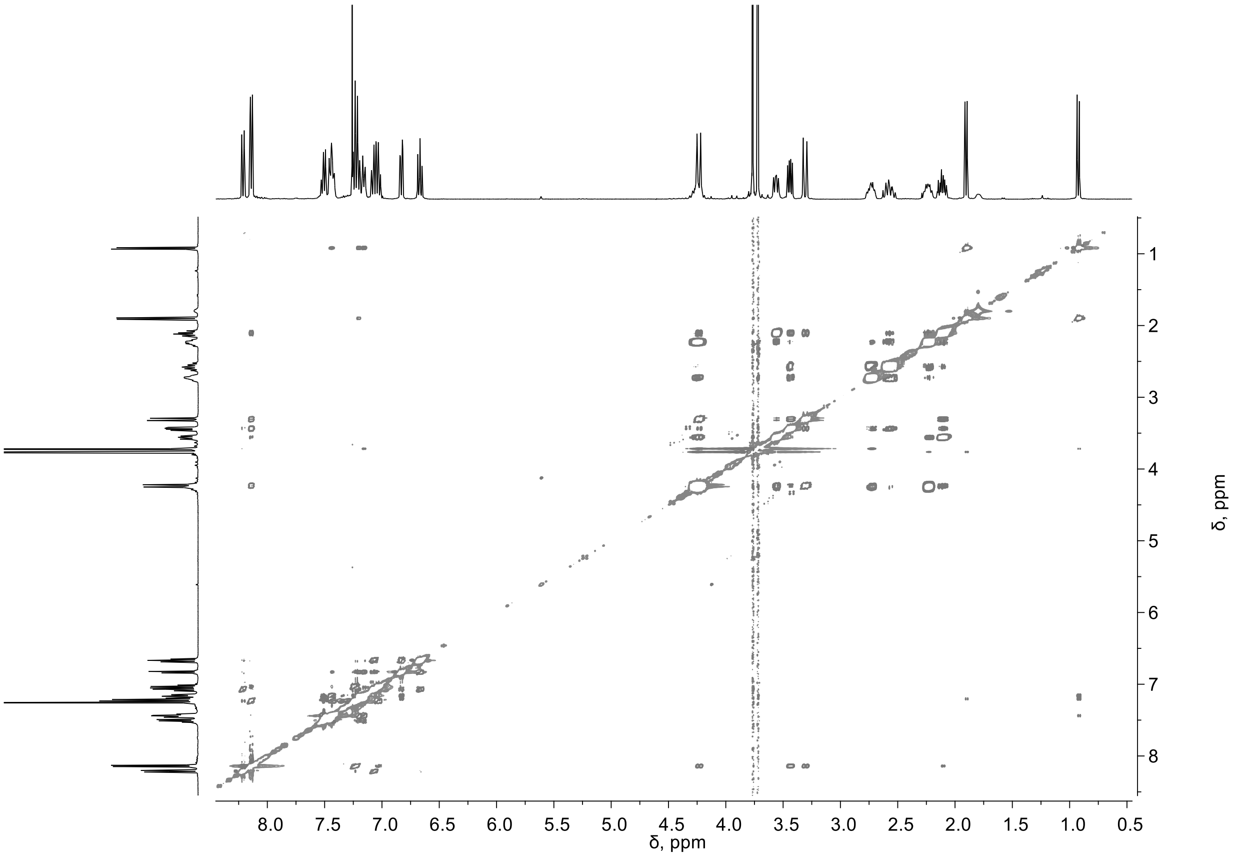

*15. Atom numeration and signal assignment in the NMR spectra of complex **5***

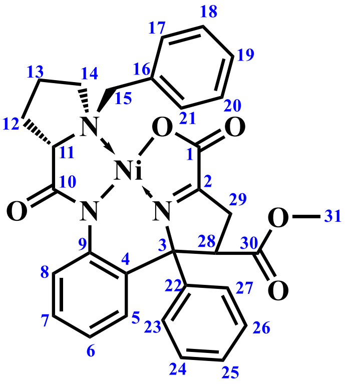

Diastereomer 1:

^{1}H NMR (CDCl$_3$ δ, ppm): 8.00-7.95 (m, 2H (H-17,21)), 7.91-7.86 (m, 2H (H-8,24)), 7.51-7.47 (m, 3H (H-18,19,20)), 7.29-7.21 (m, 4H (H-5,7,25,26)), 7.14 (dd, ^{3}J = 8.2 Hz, ^{3}J = 6.9 Hz 1H (H-6)), 7.01-6.95 (m, 2H (H-23,27)), 4.26-4.15 (m, 2H (H-14,28)), 3.95 (d, ^{2}J = 12.7 Hz, 1H (H-15)), 3.70-3.53 (m, 2H (H-11,29)), 3.28 (s, 3H (H-31)), 3.07 (dd, ^{3}J = 20.3 Hz, ^{3}J = 9.7 Hz, 1H (H-29)), 3.03-2.97 (m, 1H (H-14)), 2.95 (d, ^{2}J = 12.7 Hz, 1H (H-15)), 2.27-2.18 (m, 1H (H-13)), 2.04-1.86 (m, 3H (H-12,12,13)).

^{13}C NMR (CDCl$_3$ δ, ppm): 179.04 (C-10), 177.03 (C-2), 169.31 (C-30), 166.75 (C-1), 142.12 (C-22), 136.97 (C-9), 133.58 (C-16), 131.43 (C-17,21), 131.26 (C-4), 129.34, 129.31 (C-18,19,20), 128.77, 128.72, 128.66 (C-5,7,25,26), 127.48 (C-24), 126.54 (C-8), 126.22 (C-23,27), 122.76 (C-6), 79.36 (C-3), 68.32 (C-11), 59.60 (C-15), 57.94 (C-14), 53.72 (C-28), 52.53 (C-31), 36.22 (C-29), 27.43 (C-13), 22.20 (C-12).

Diastereomer 2:

^{1}H NMR (CDCl$_3$ δ, ppm): 8.25-8.21 (m, 2H (H-17,21)), 7.75-7.70 (m, 2H (H-23,27)), 7.46-7.26 (m, 7H (H-8,18,19,20,24,25,26)), 7.15 (dd, ^{3}J = 7.7 Hz, ^{4}J = 1.6 Hz, 1H (H-5)), 6.98-6.93 (m, 1H (H-7)), 6.88 (td, J = 7.5 Hz, J = 1.4 Hz, 1H (H-6)), 4.30 (d, ^{2}J = 12.5 Hz, 1H (H-15)), 3.88 (dd, ^{3}J = 5.9 Hz, ^{3}J = 1.5 Hz, 1H (H-28)), 3.80-3.66 (m, 1H (H-13)), 3.58 (s, 3H (H-31)), 3.57-3.46 (m, 2H (H-14,15)), 3.33 (dd, ^{3}J = 10.4 Hz, ^{3}J = 6.2 Hz, 1H (H-11)), 2.88 (dd, ^{2}J = 19.1 Hz, ^{3}J = 1.5 Hz, 1H (H-29)), 2.82 (dd, ^{2}J = 19.1 Hz, ^{3}J = 5.9 Hz, 1H (H-29)), 2.50-2.34 (m, 2H (H-12)), 2.33-2.23 (m, 1H (H-13)), 2.23-2.13 (m, 1H (H-14)).

^{13}C NMR (CDCl$_3$ δ, ppm): 179.34 (C-10), 178.59 (C-2), 171.82 (C-30), 166.69 (C-1), 139.46 (C-22), 139.37 (C-9), 133.66 (C-16), 131.54 (C-17,21), 130.42 (C-4), 129.38 (C-19), 129.33 (C-18,20), 129.19 (C-24,26), 128.46 (C-25), 127.37 (C-7), 126.74 (C-8), 125.44 (C-5,23,27), 122.29 (C-6), 77.80 (C-3), 71.75 (C-11), 63.36 (C-15), 57.84 (C-14), 56.52 (C-28), 52.56 (C-31), 36.96 (C-29), 30.55 (C-12), 24.66 (C-13).

16. ^{1}H NMR spectrum of complex ***5****, diastereomer 1*

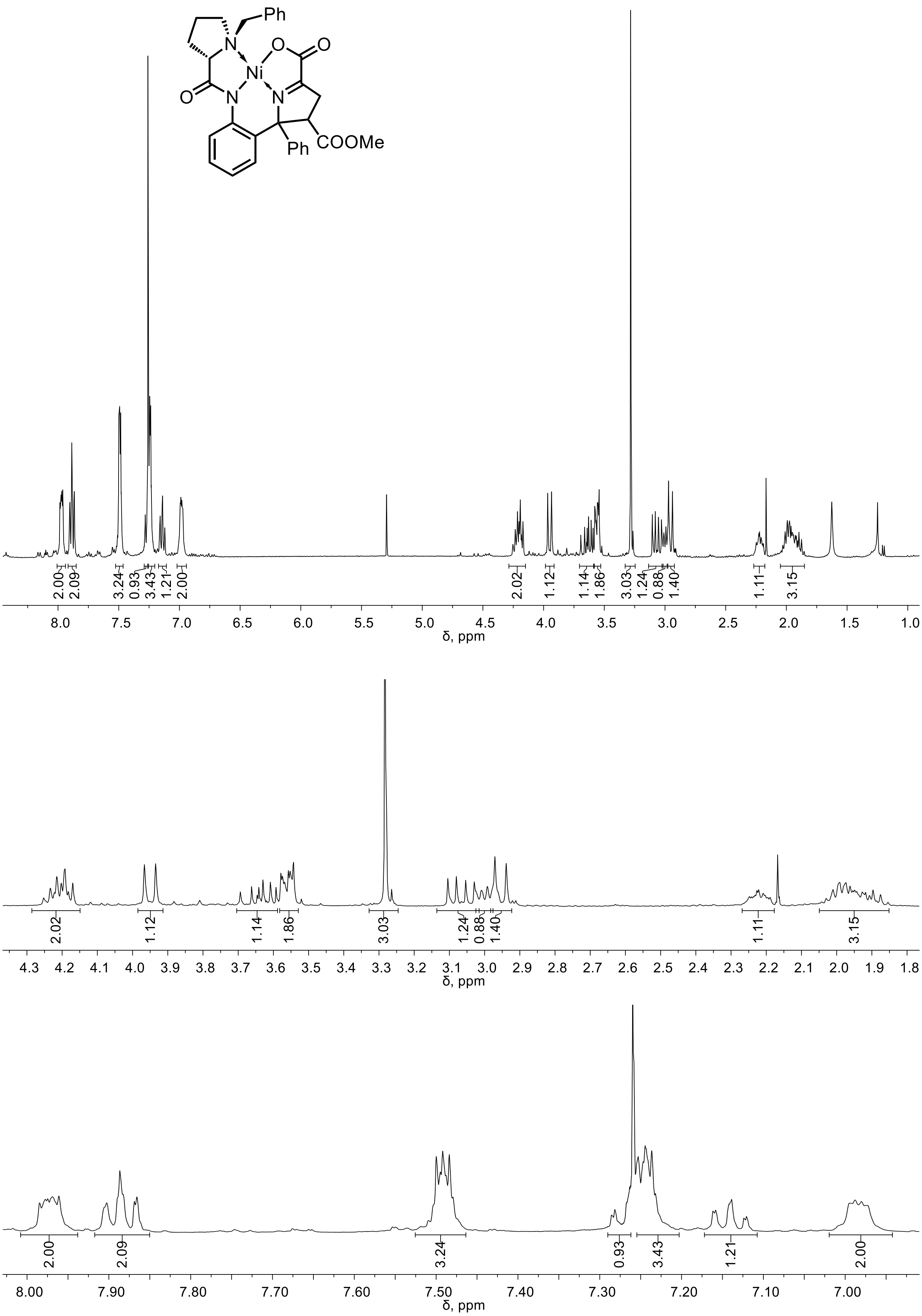

*17. ^{13}C NMR spectrum of complex **5**, diastereomer 1*

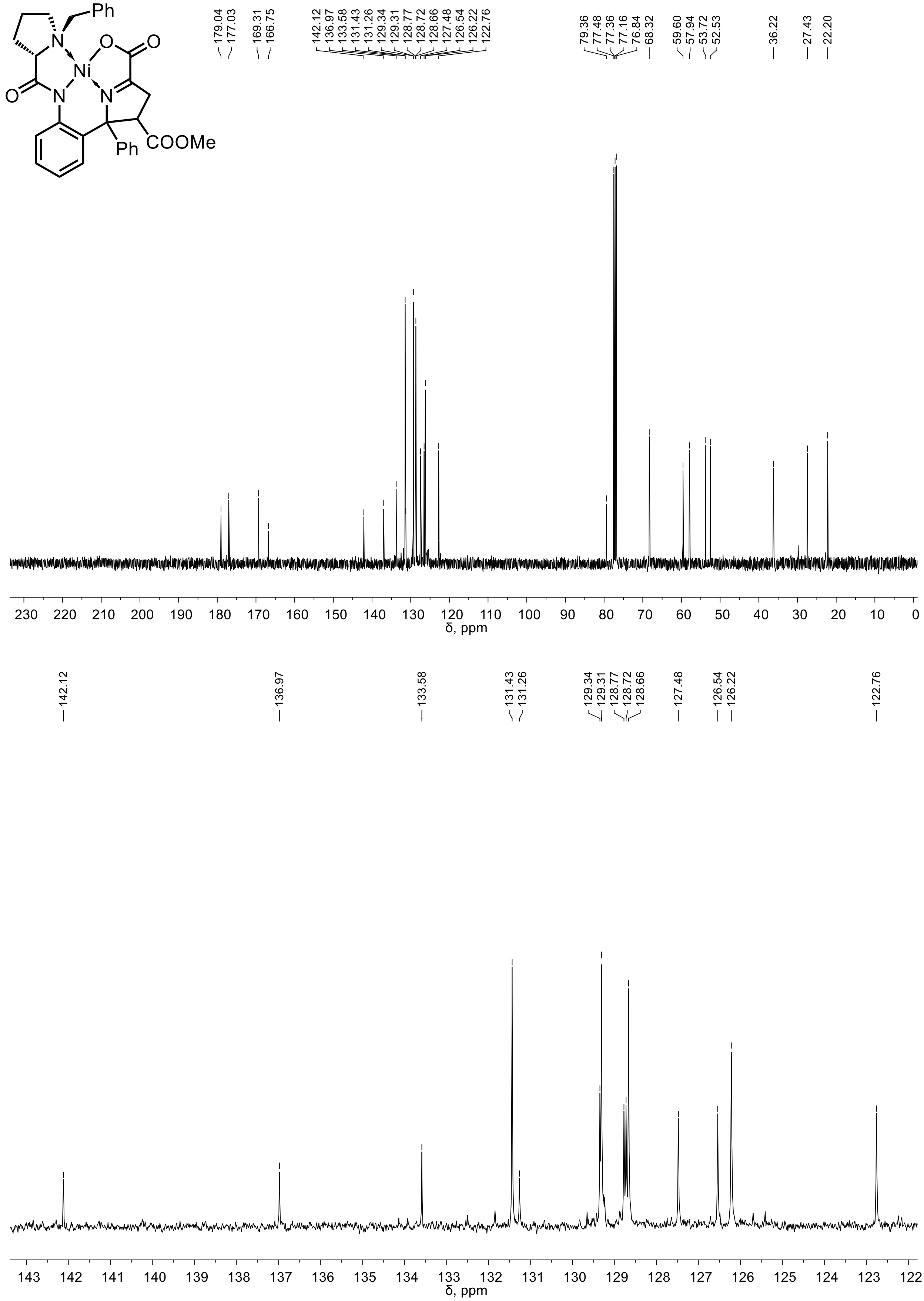

*18. COSY spectrum of complex **5**, diastereomer 1*

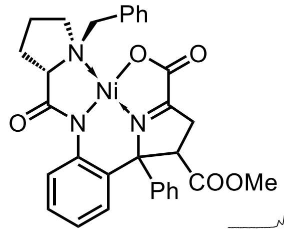

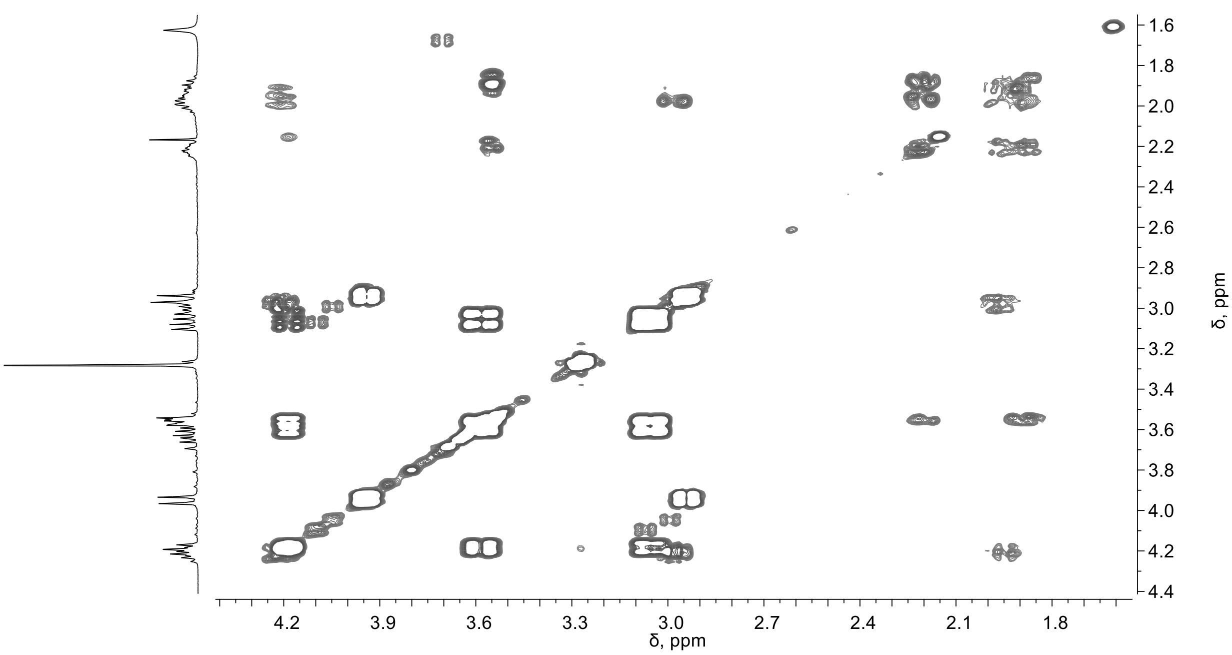

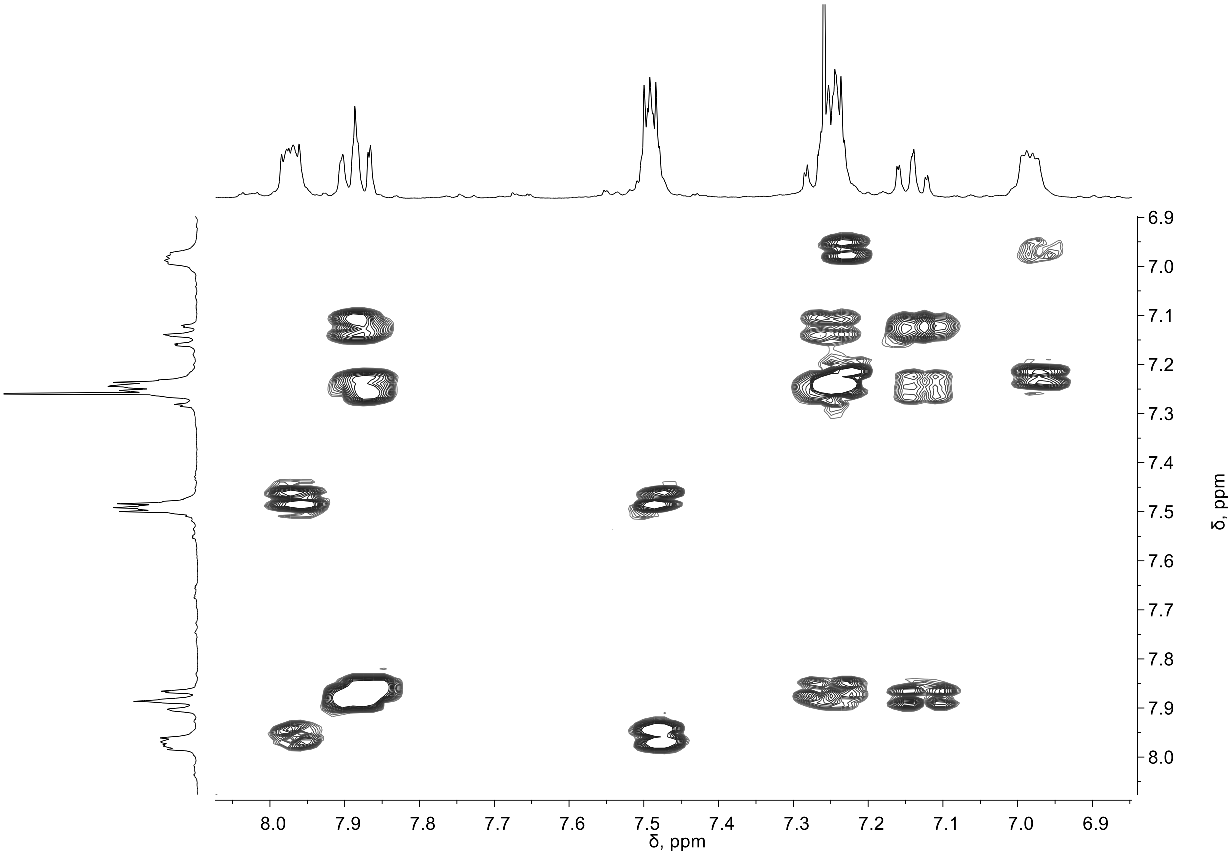

*19. HSQC spectrum of complex **5**, diastereomer 1*

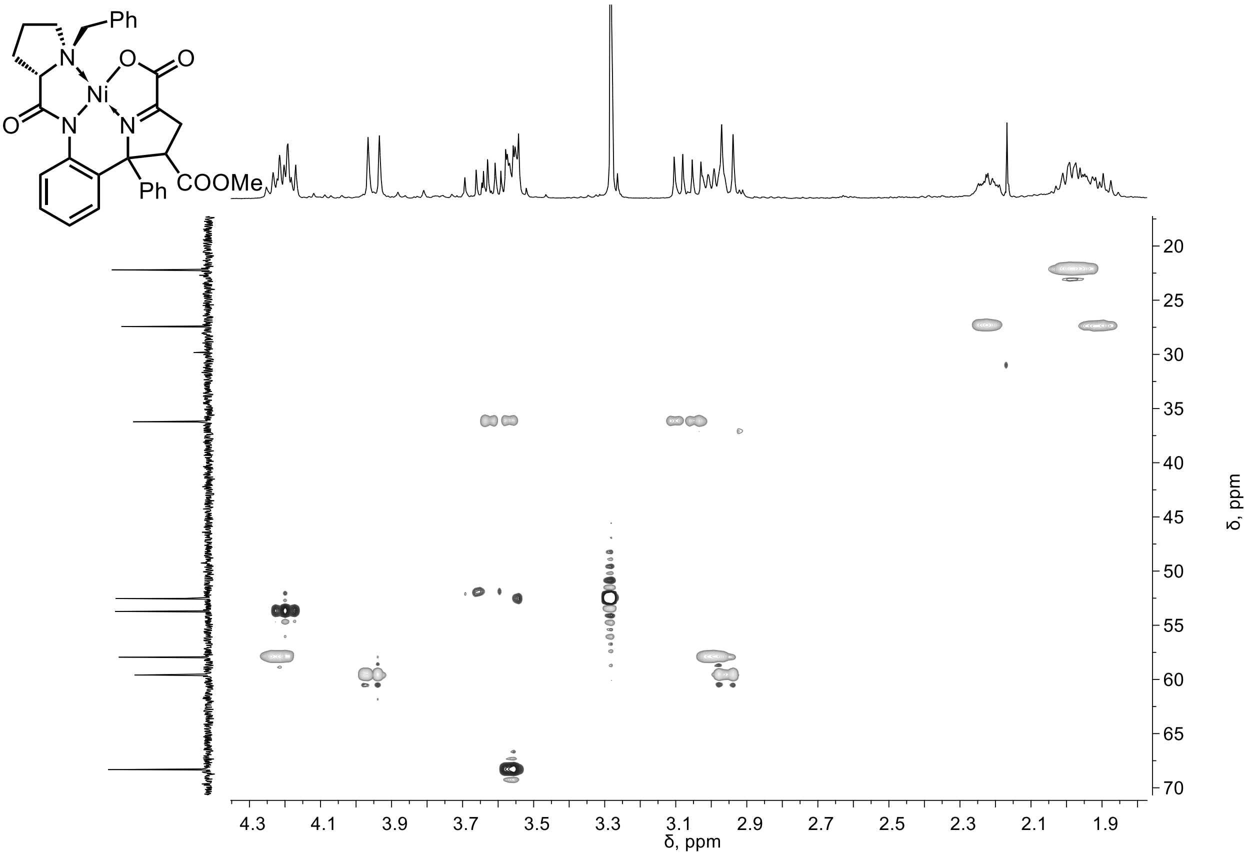

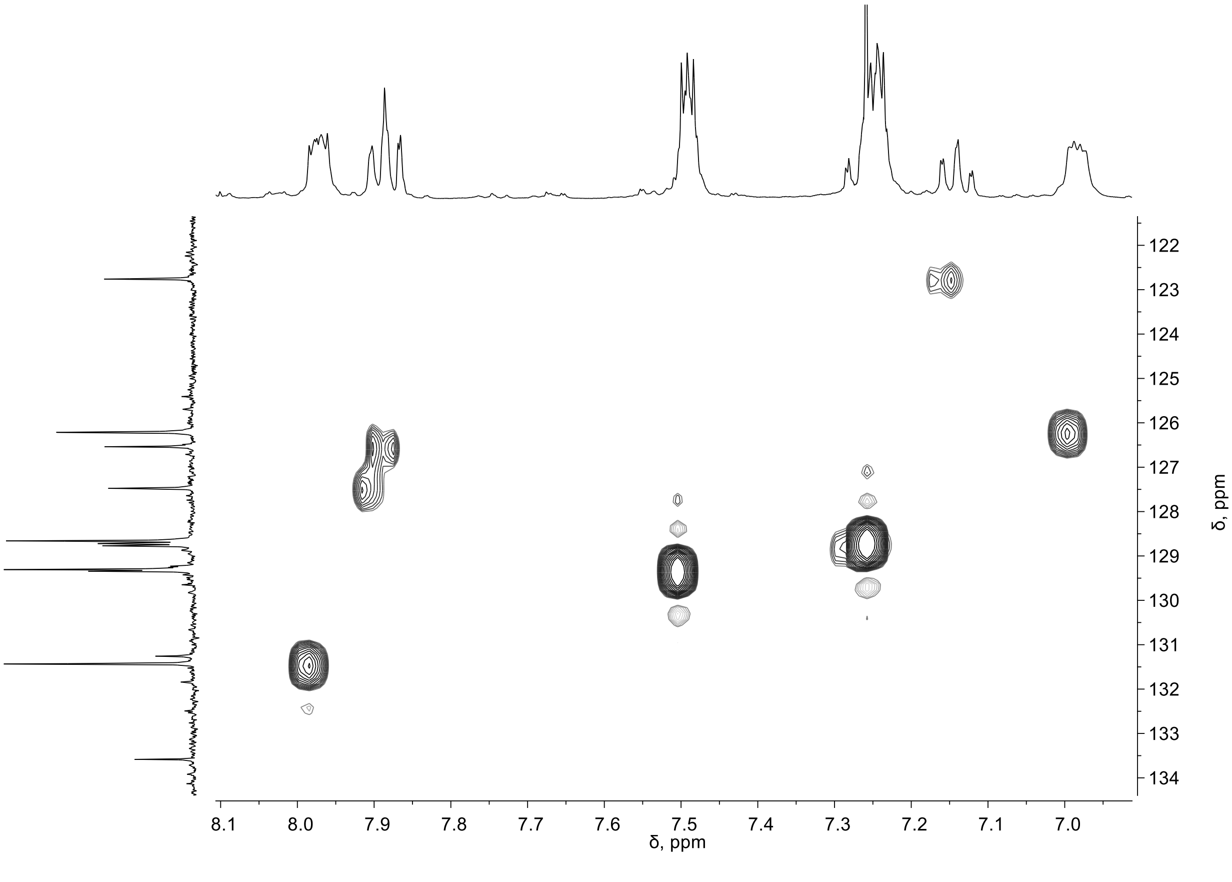

*20. HMBC spectrum of complex **5**, diastereomer 1*

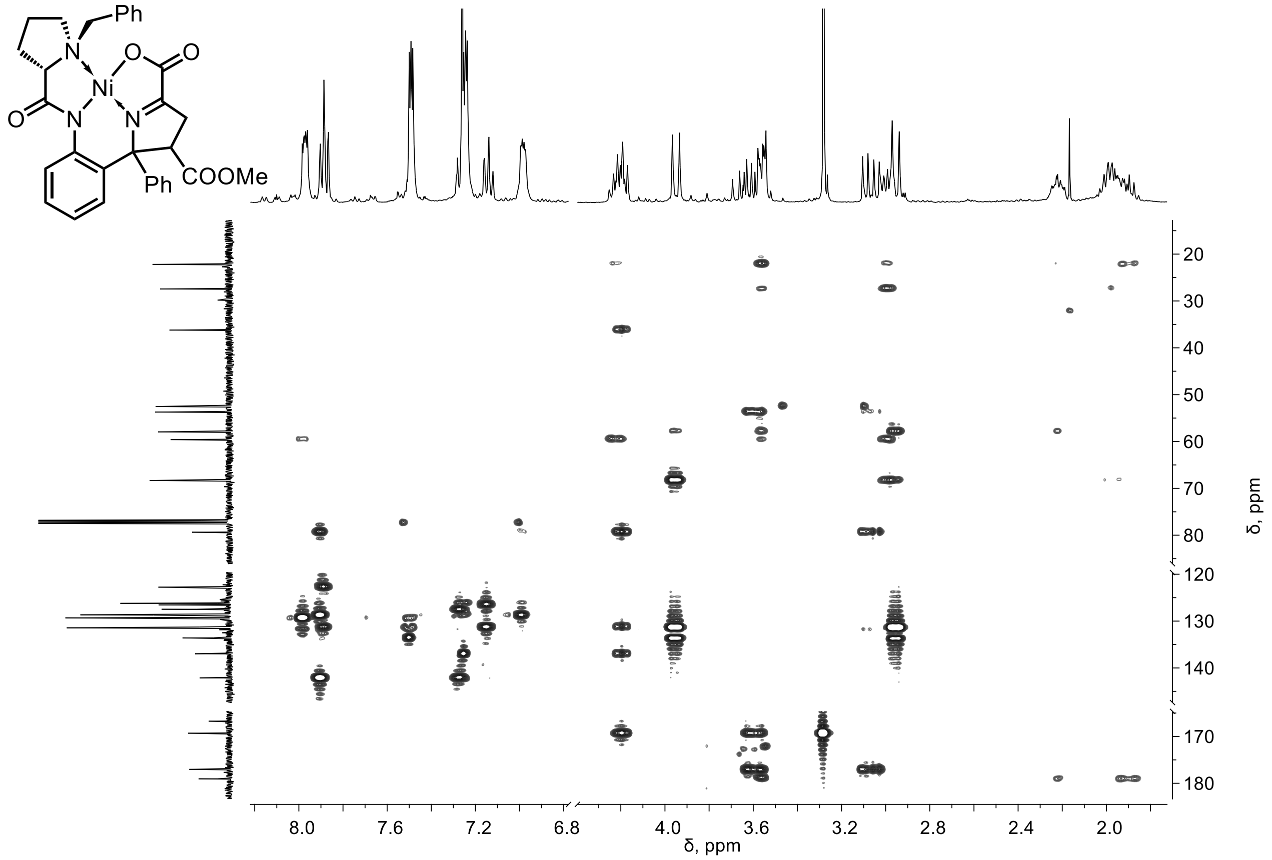

*21. ^{1}H NMR spectrum of complex **5**, diastereomer 2*

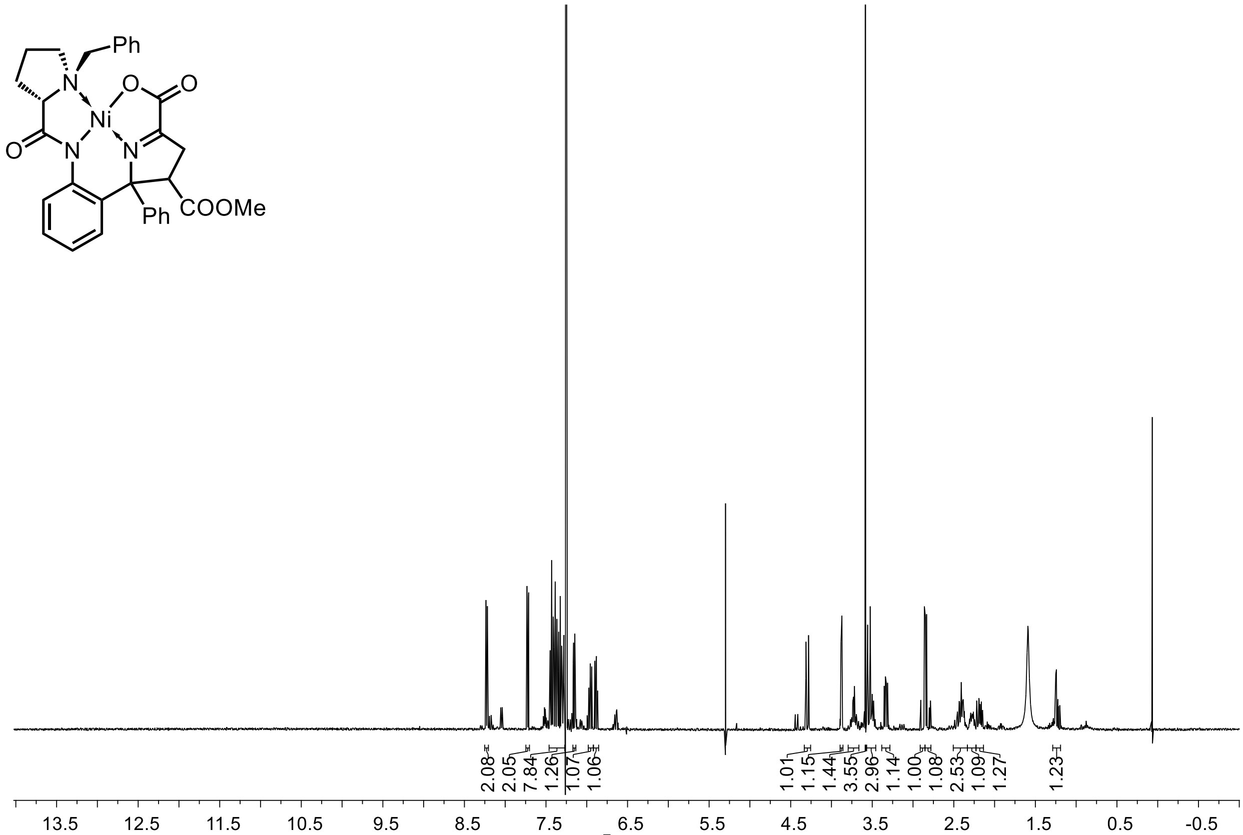

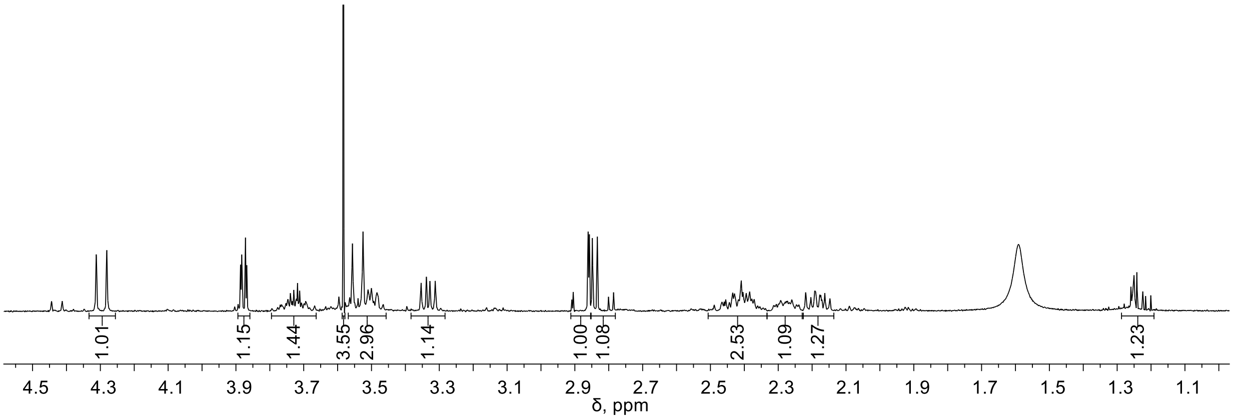

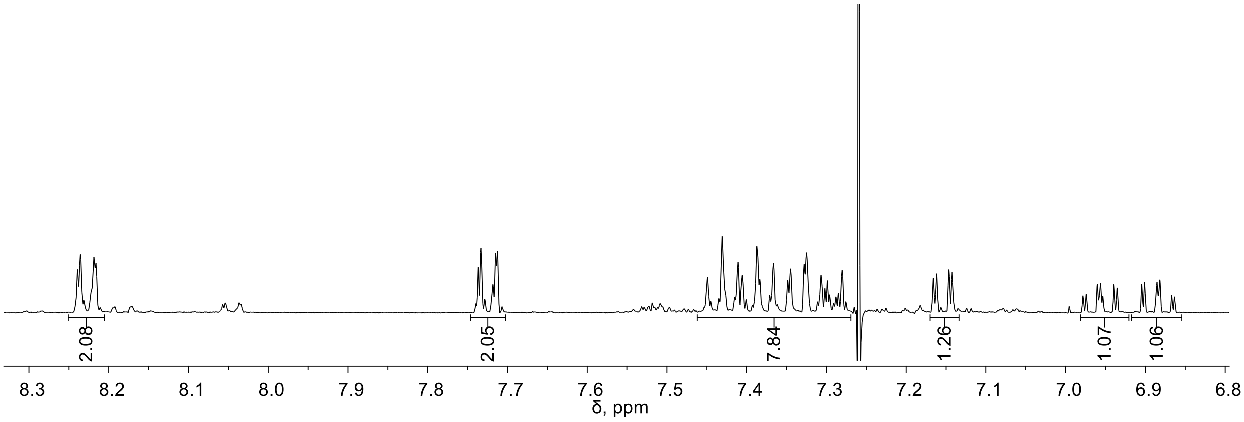

*22. ^{13}C NMR spectrum of complex **5**, diastereomer 2*

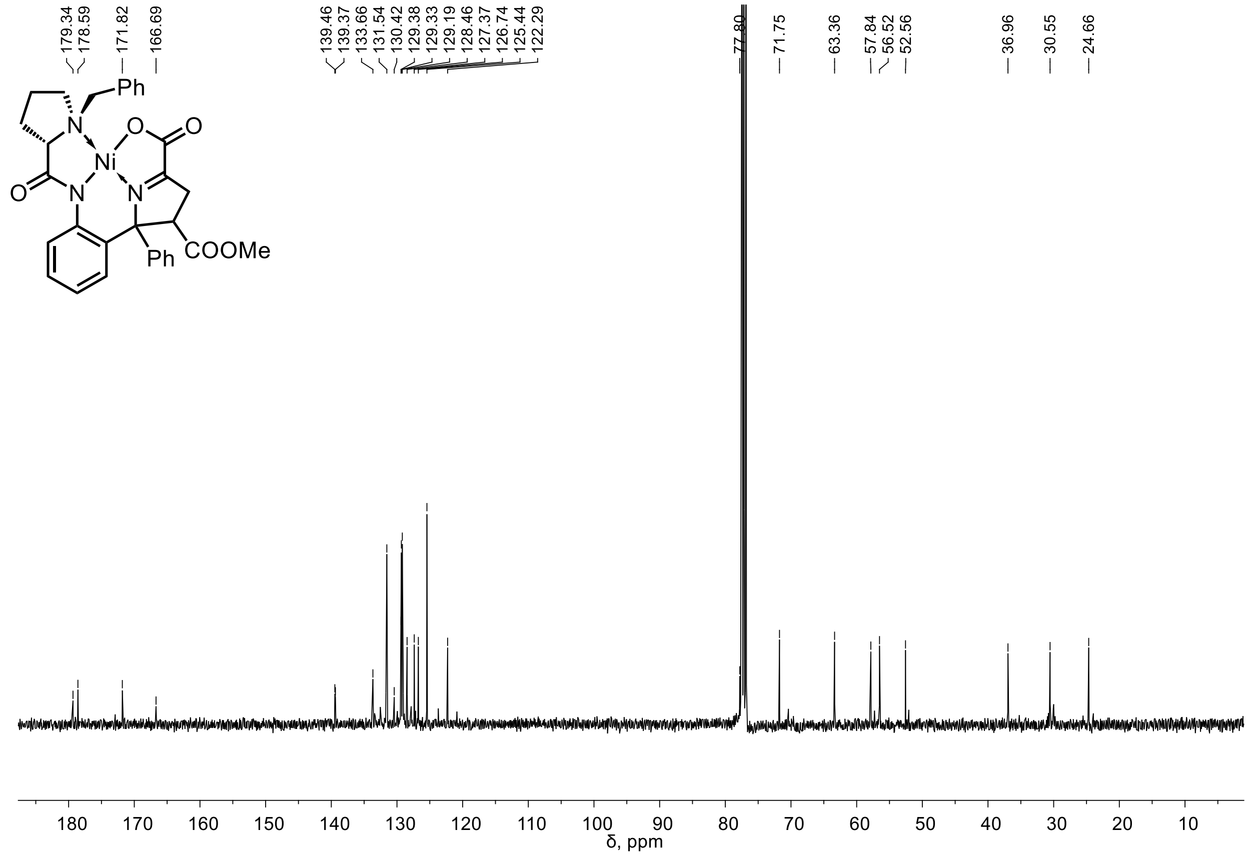

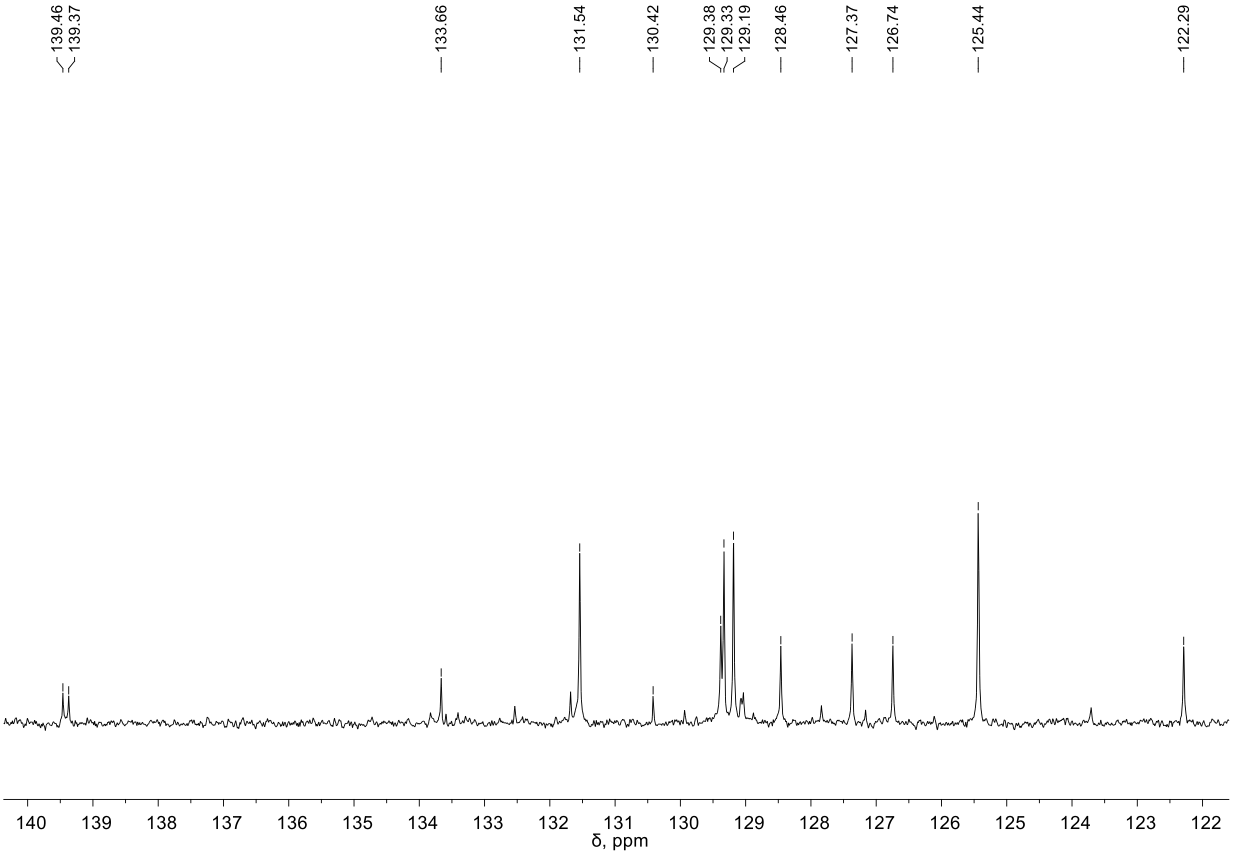

*23. COSY spectrum of complex **5**, diastereomer 2*

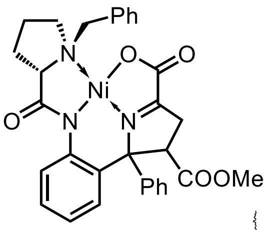

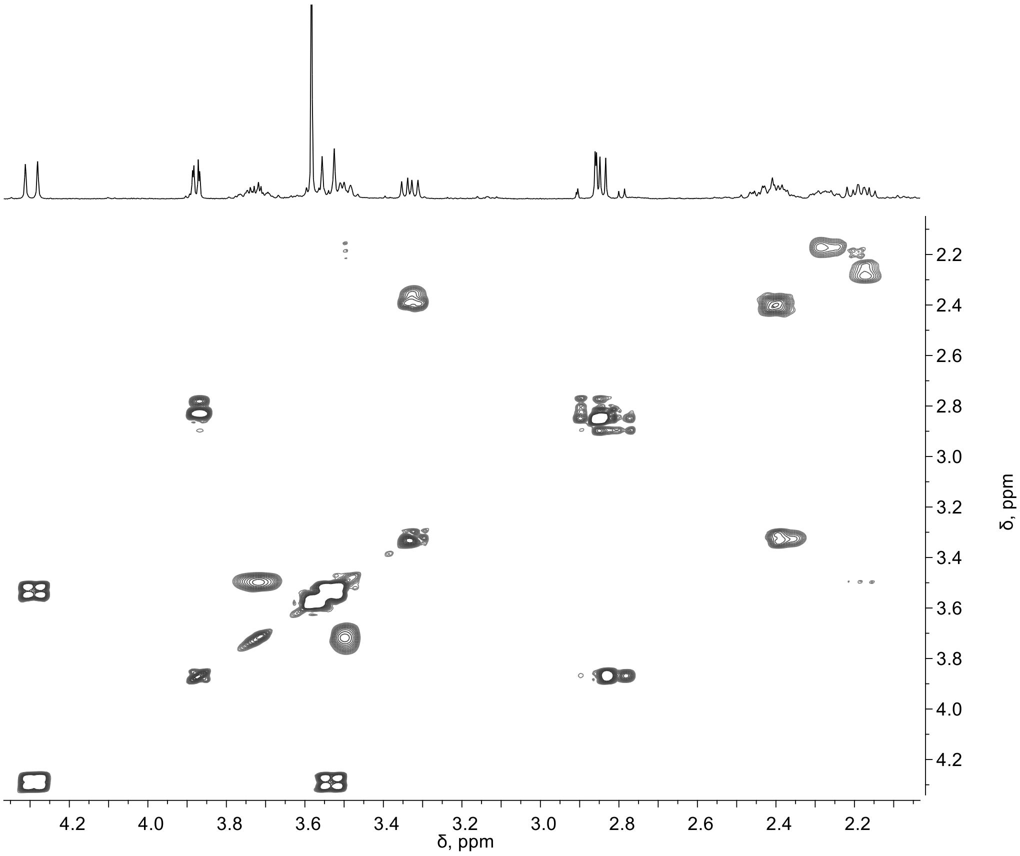

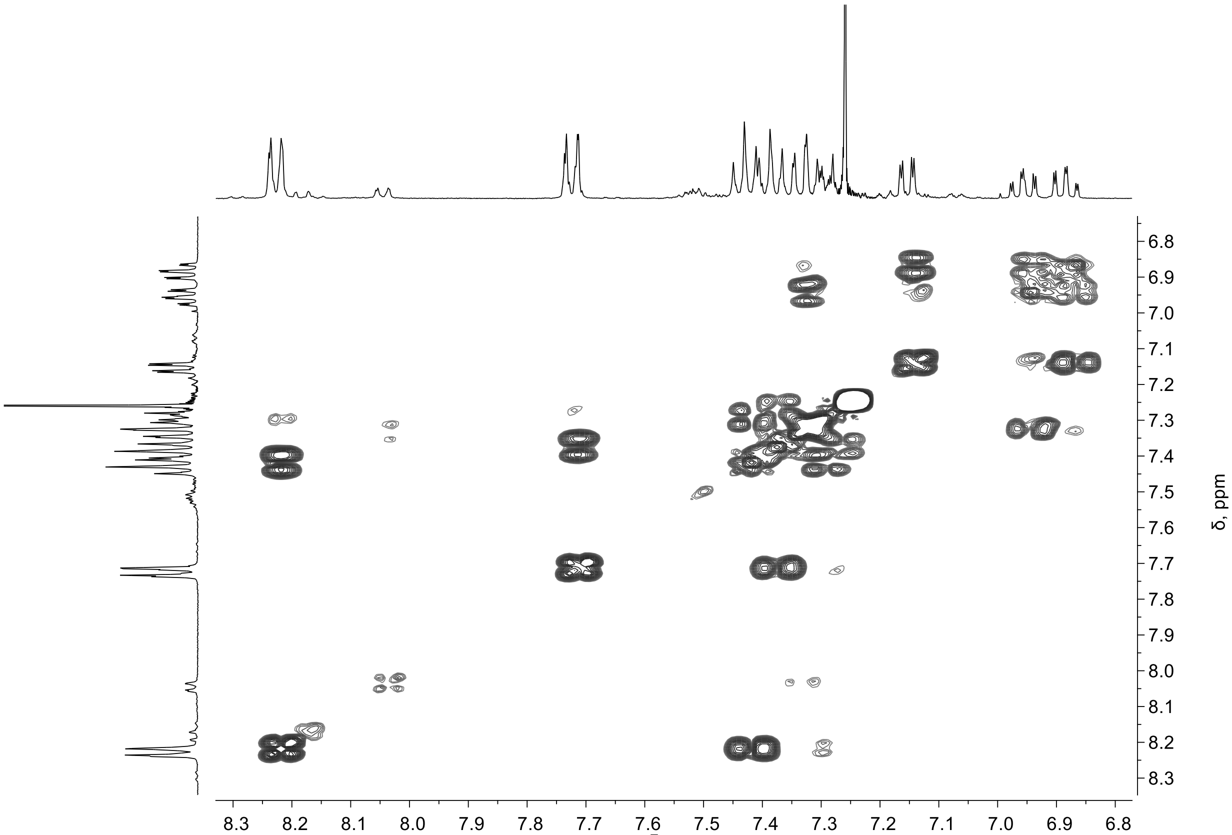

*24. HSQC spectrum of complex **5**, diastereomer 2*

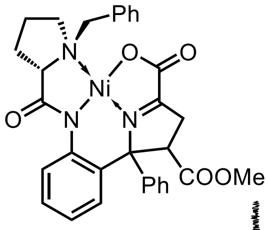

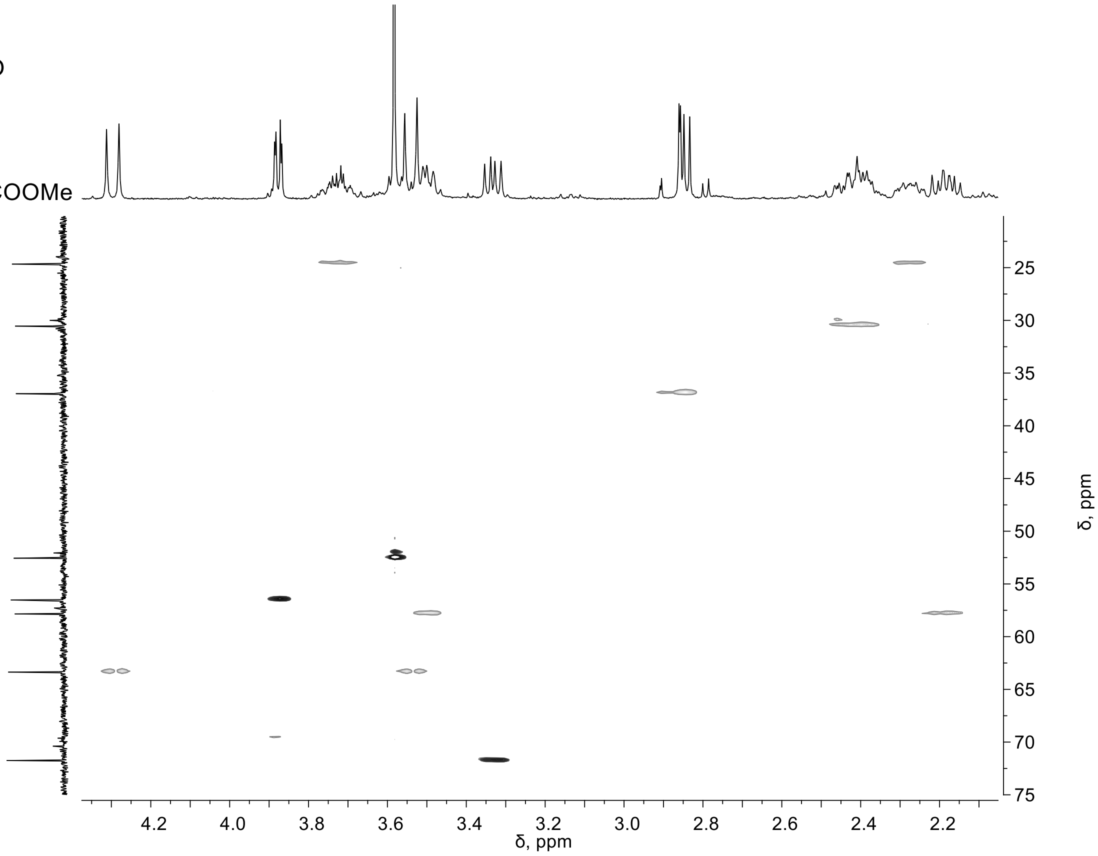

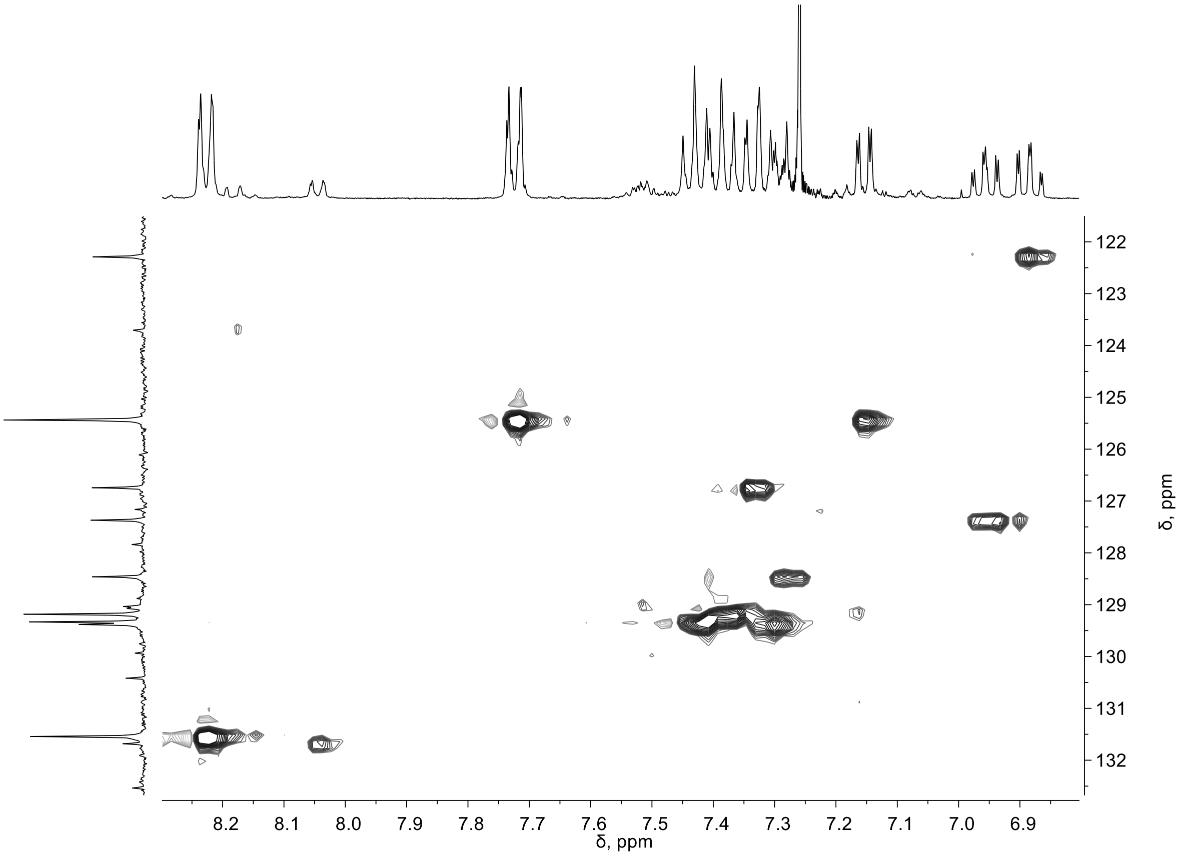

*25. HMBC spectrum of complex **5**, diastereomer 2*

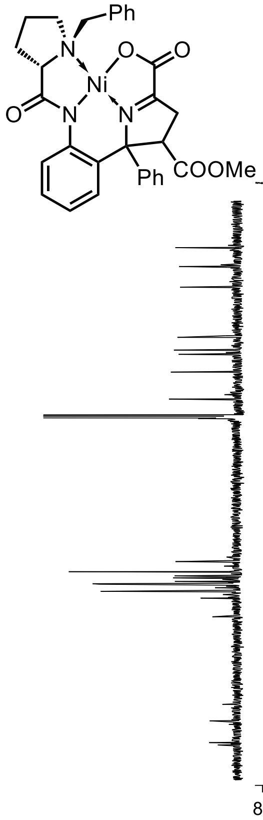

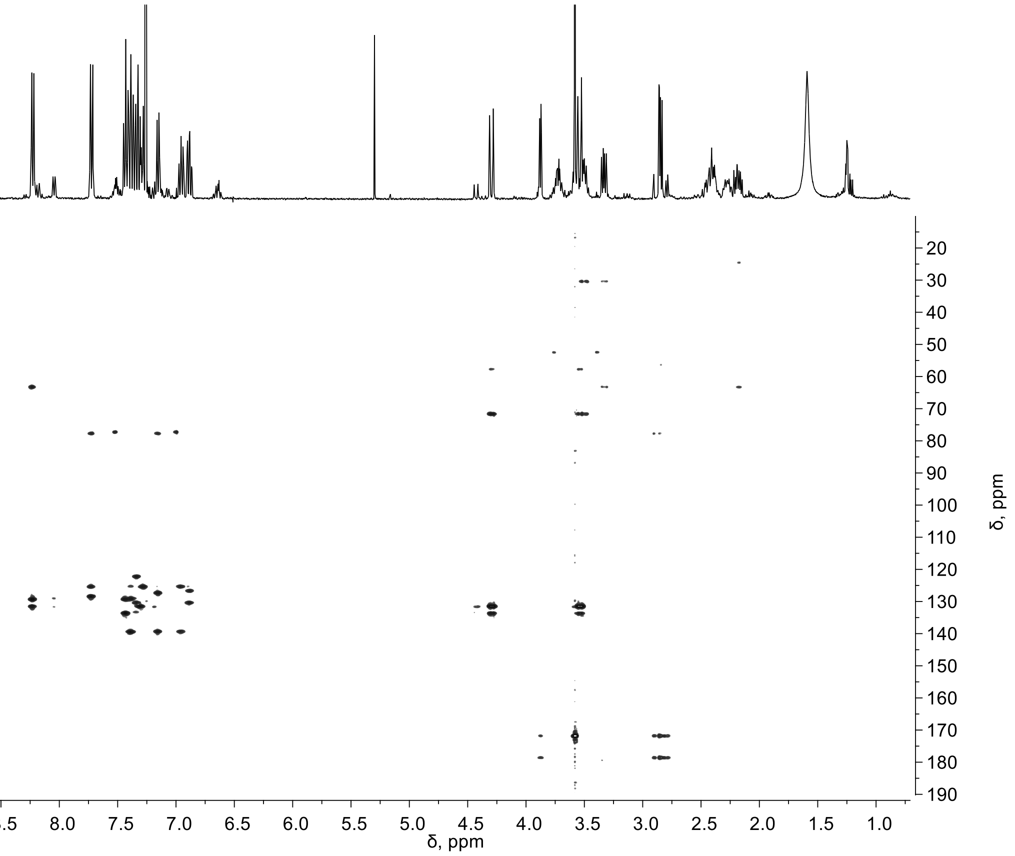

26. ^{1}H NMR spectrum of complexes ***6***

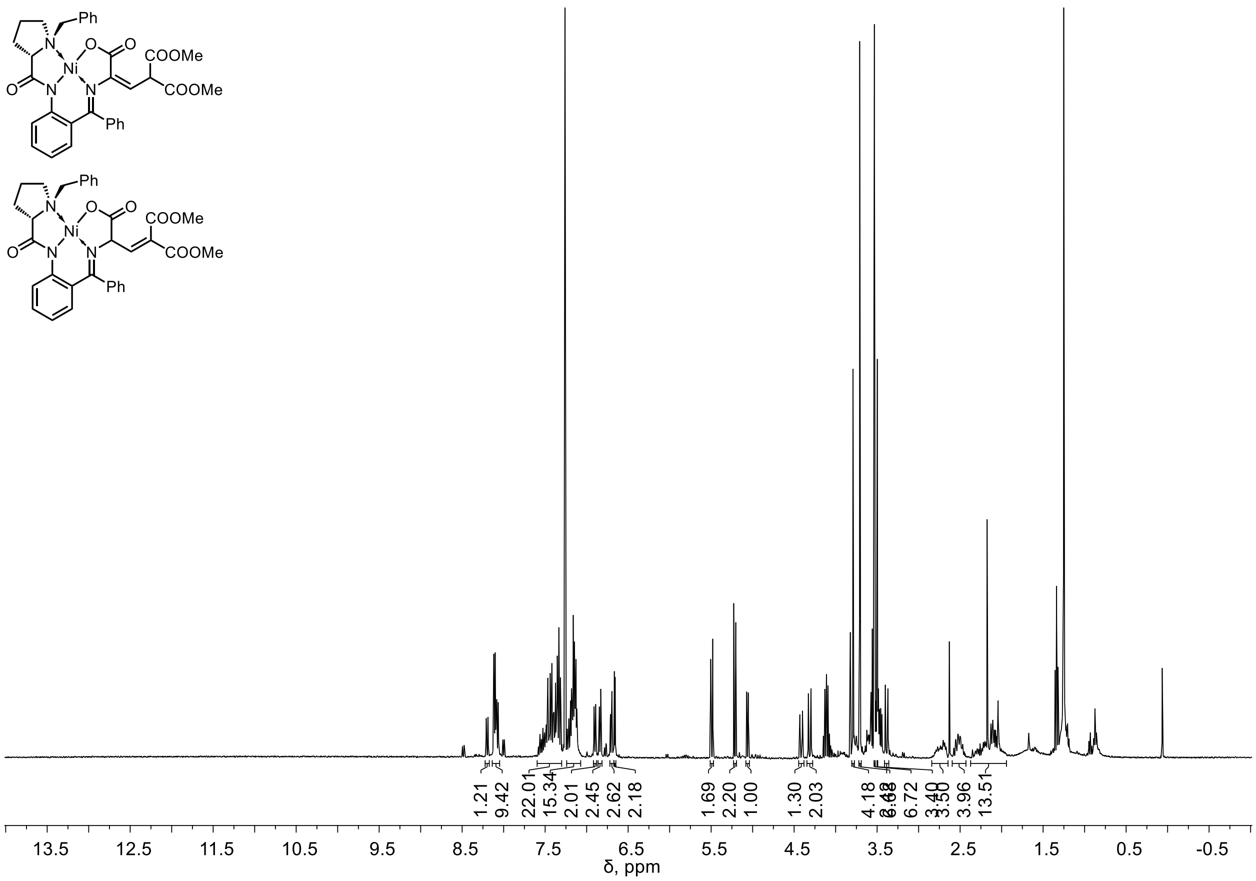

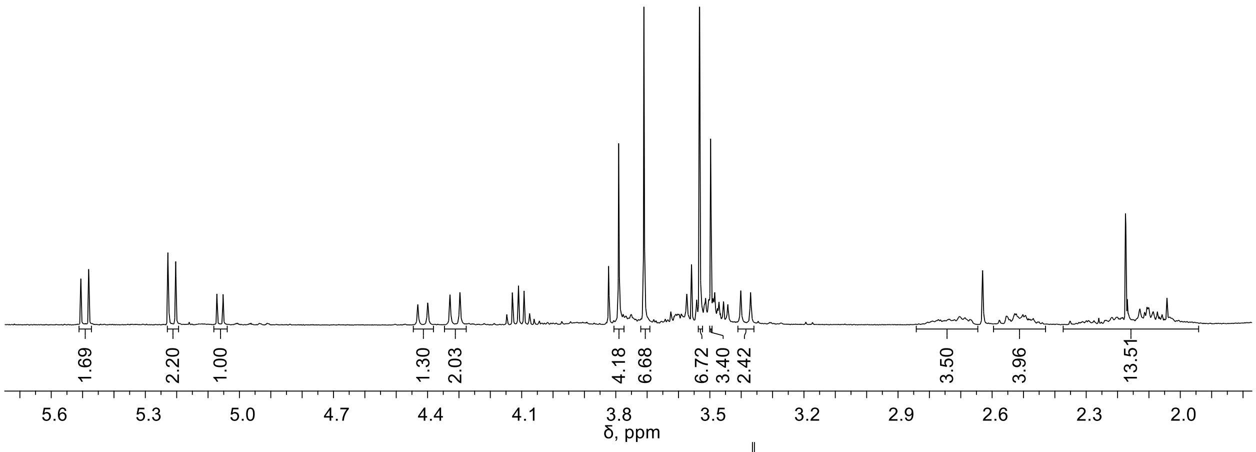

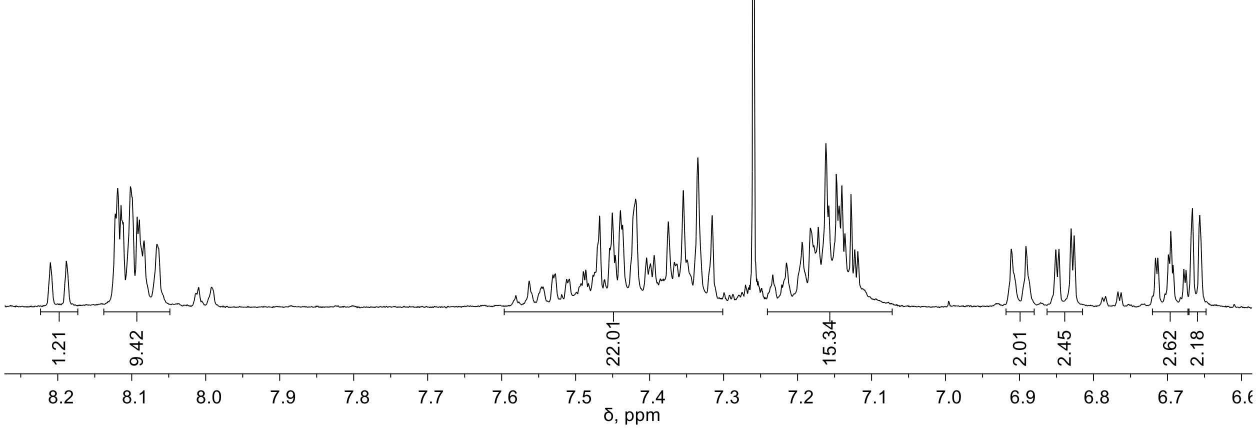

*27. ¹³C NMR spectrum of complexes **6***

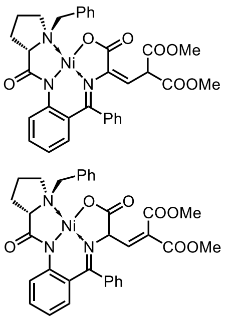

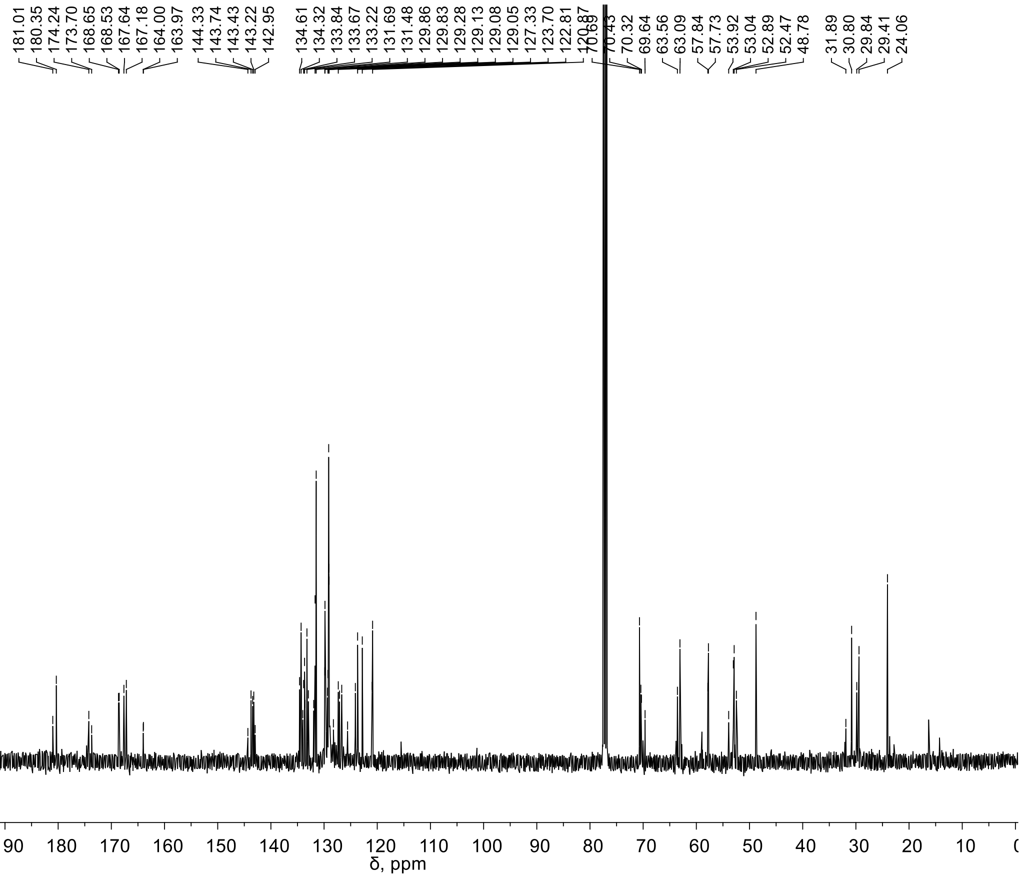

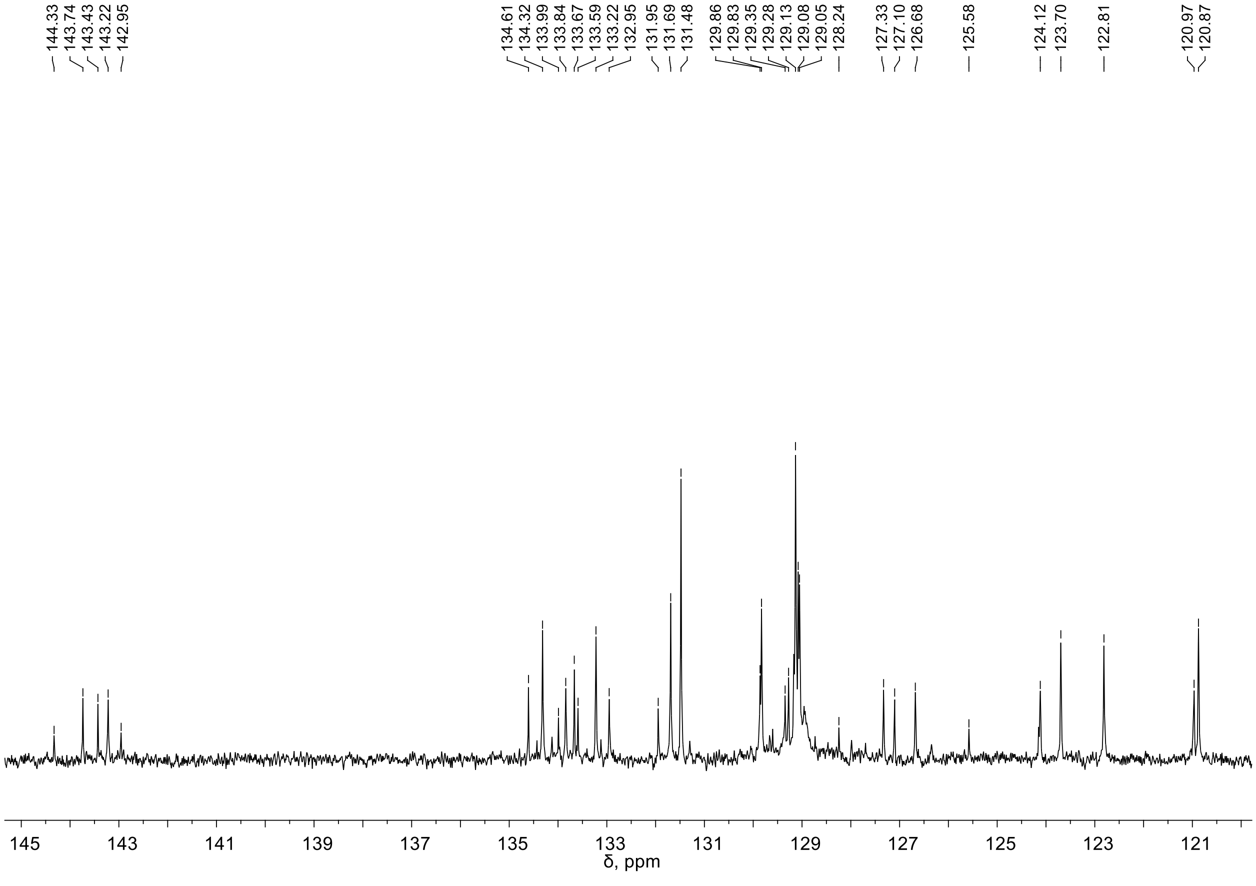

*28. COSY spectrum of complexes **6***

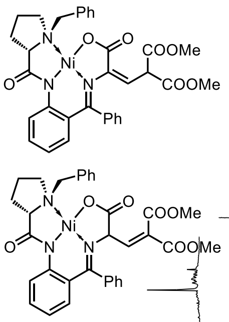

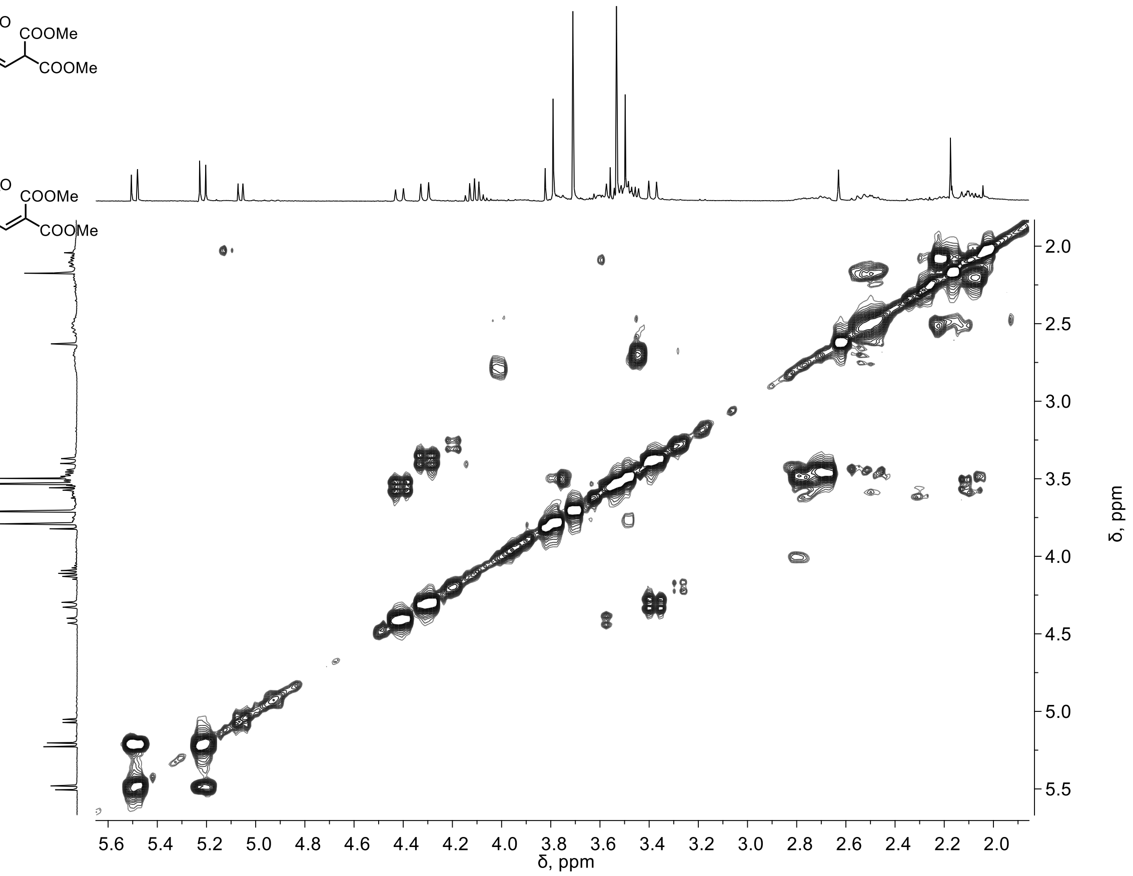

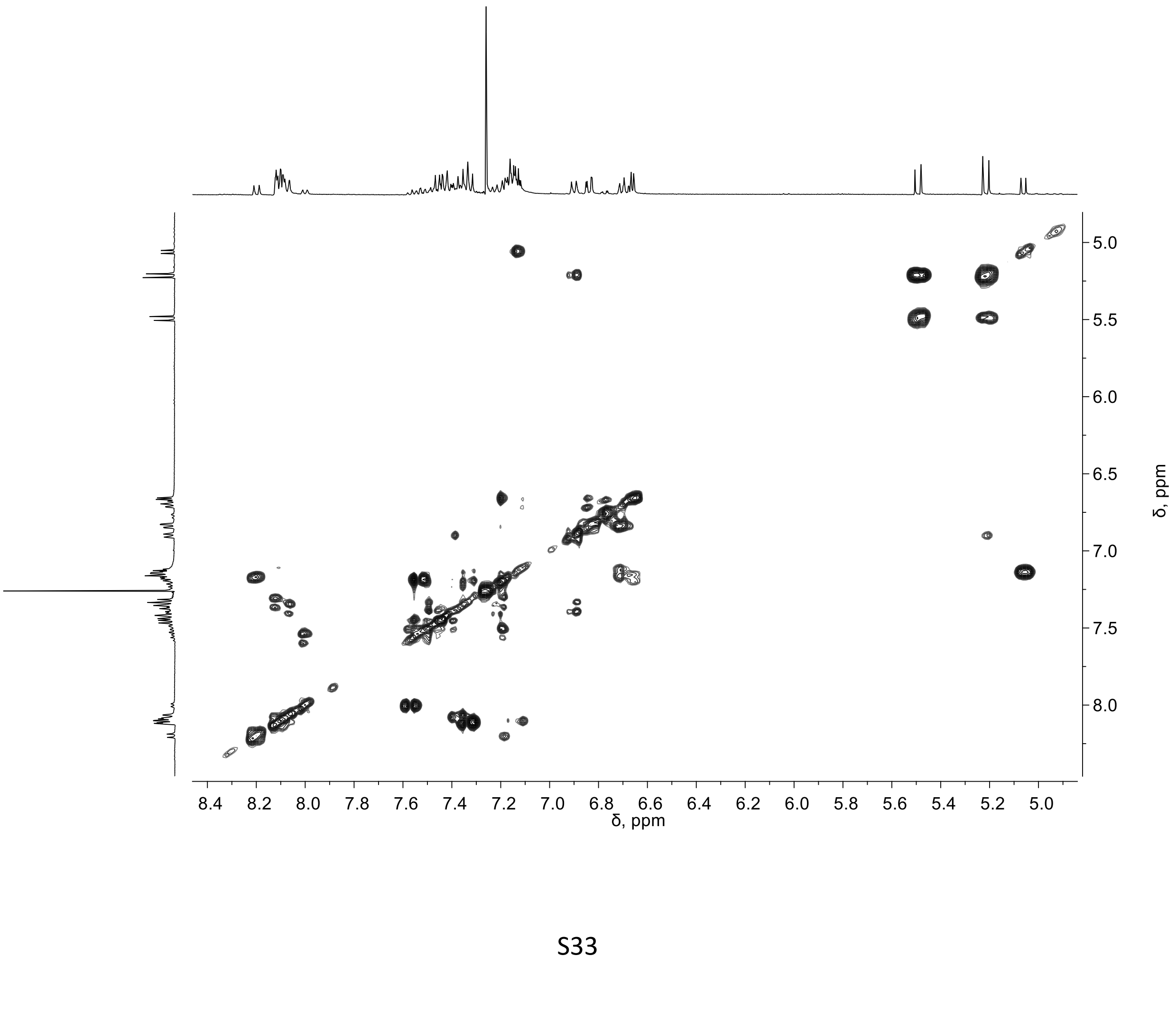

*29. HSQC spectrum of complexes **6***

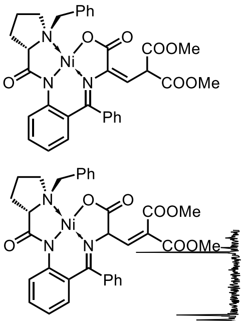

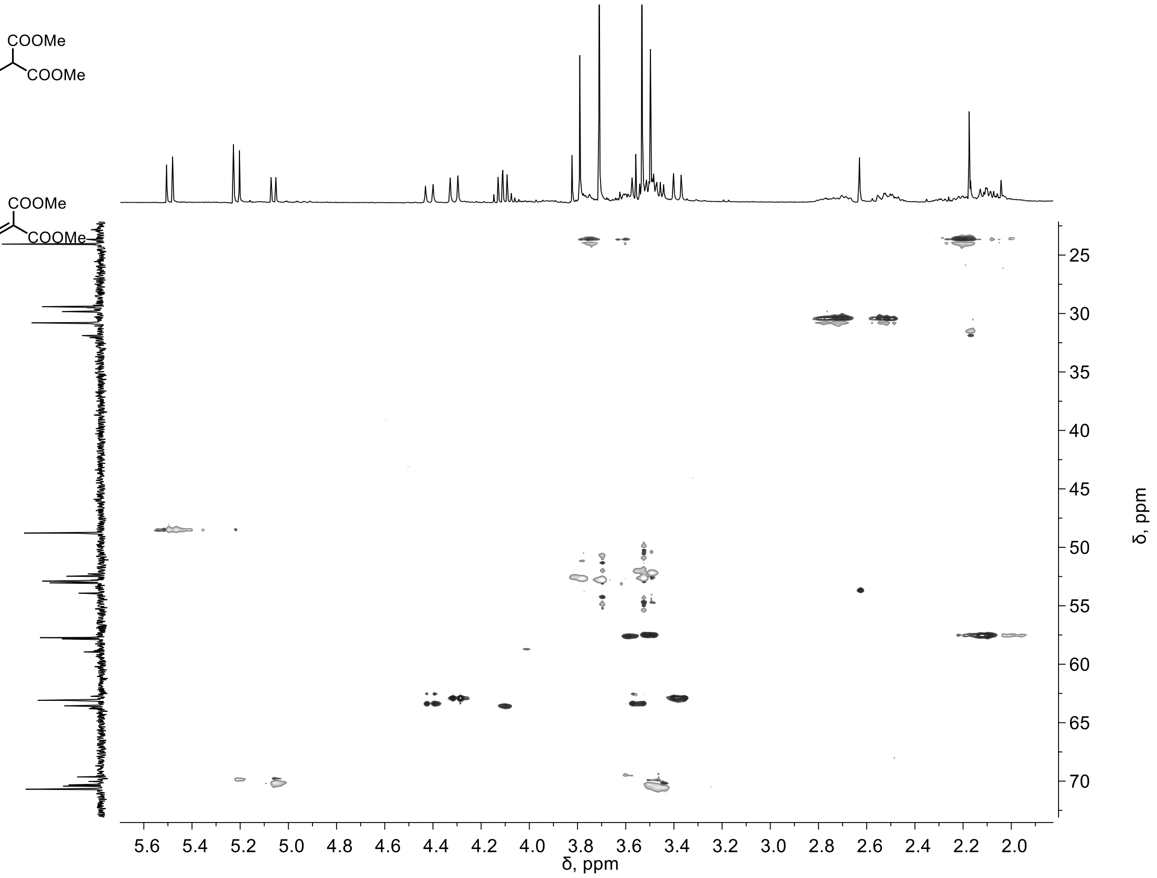

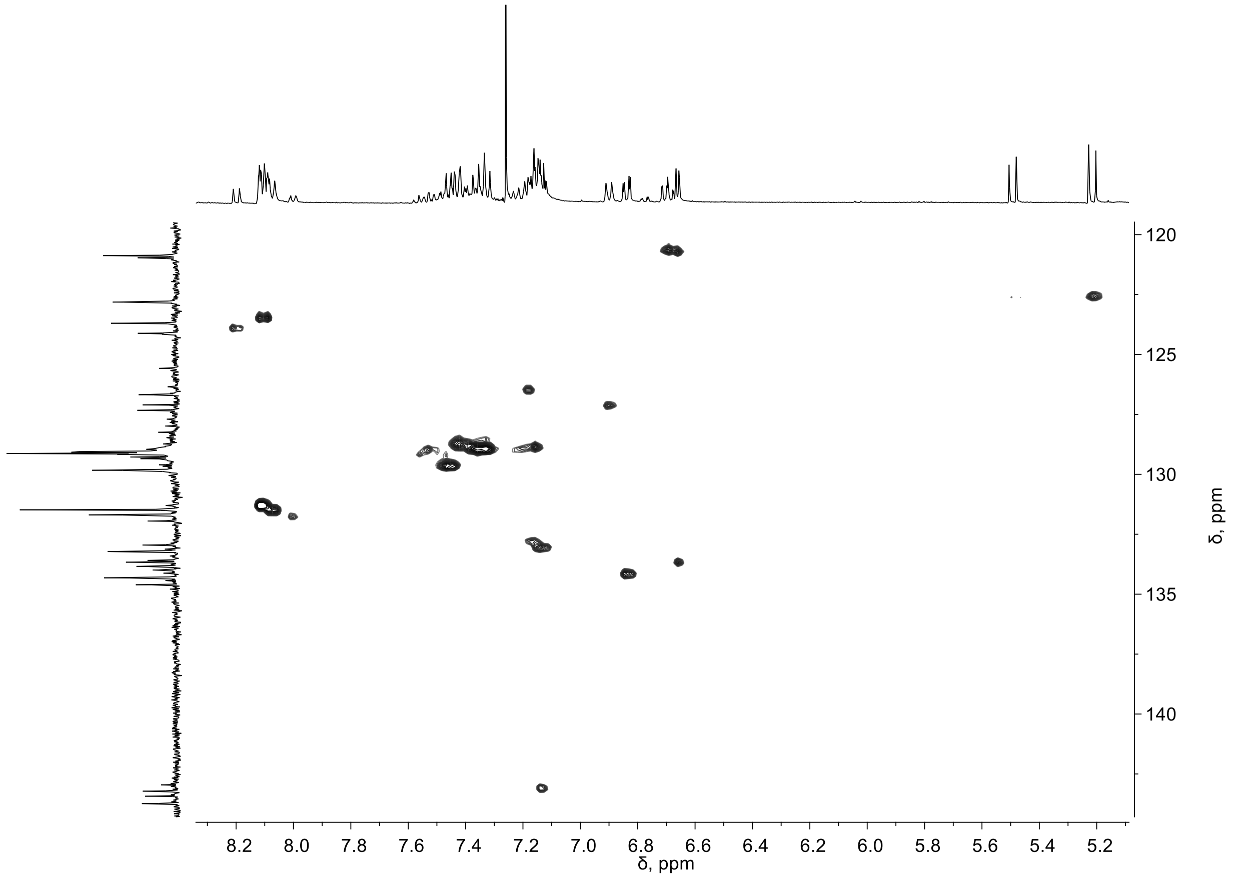

30. HMBC spectrum of complexes ***6***

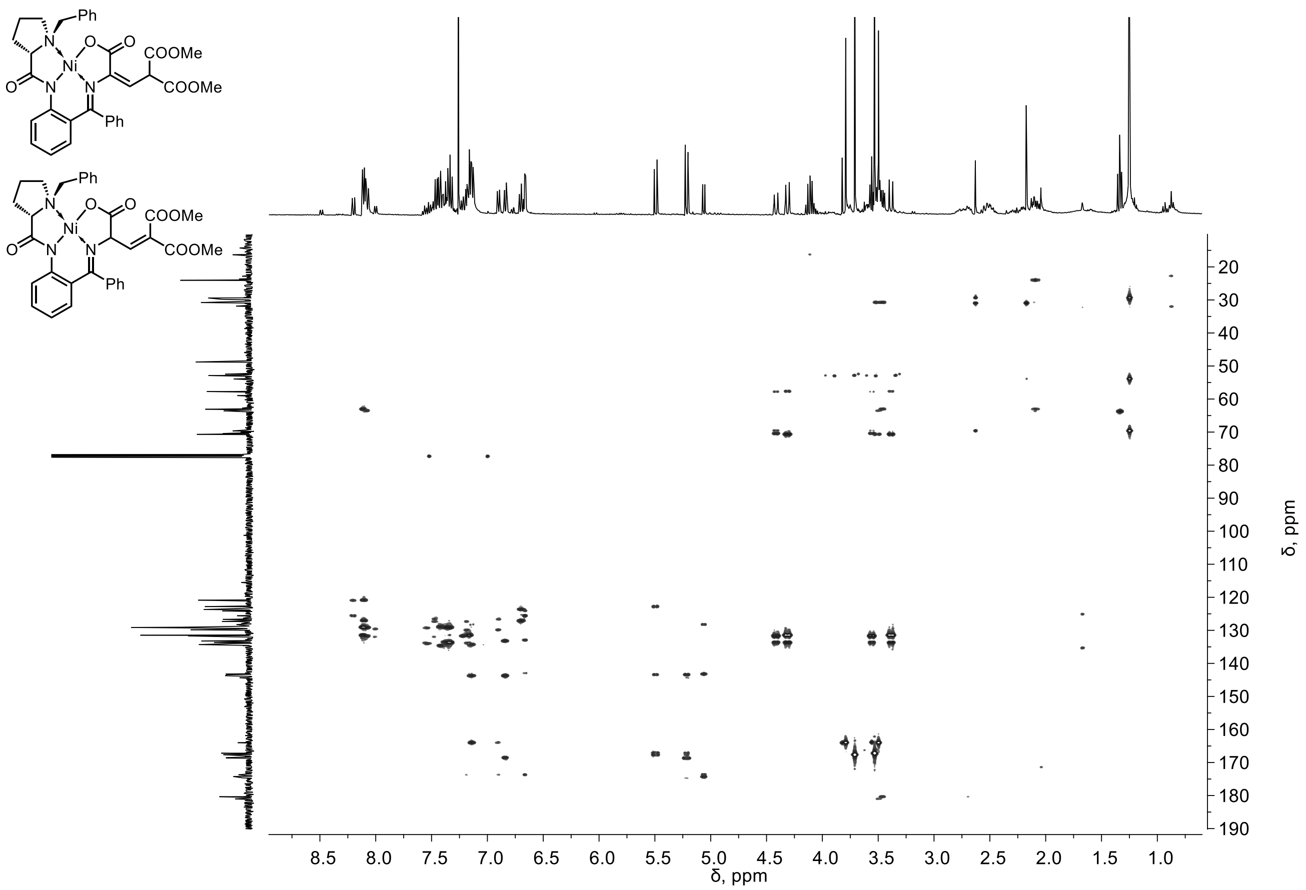

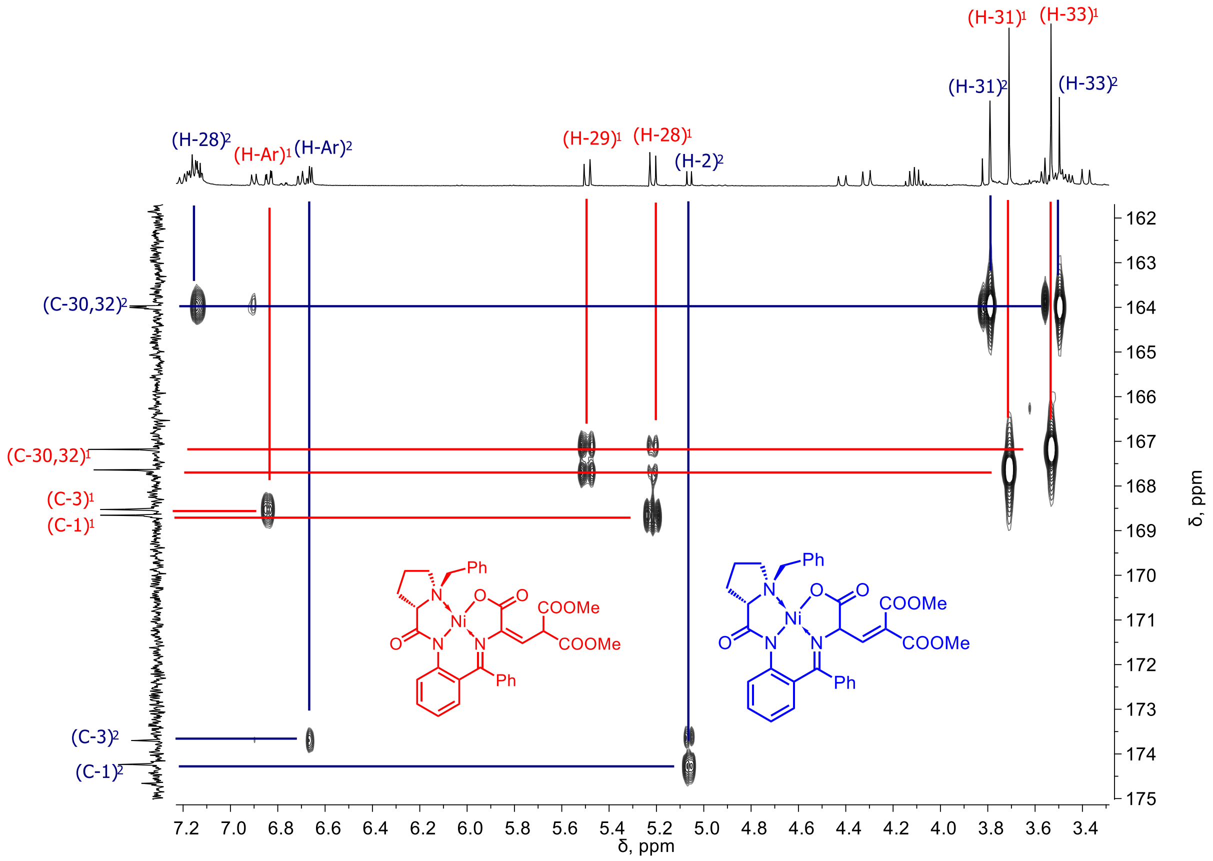

*31. ^{1}H NMR spectrum of complex **8***

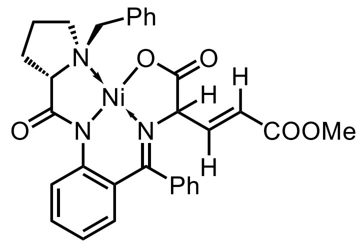

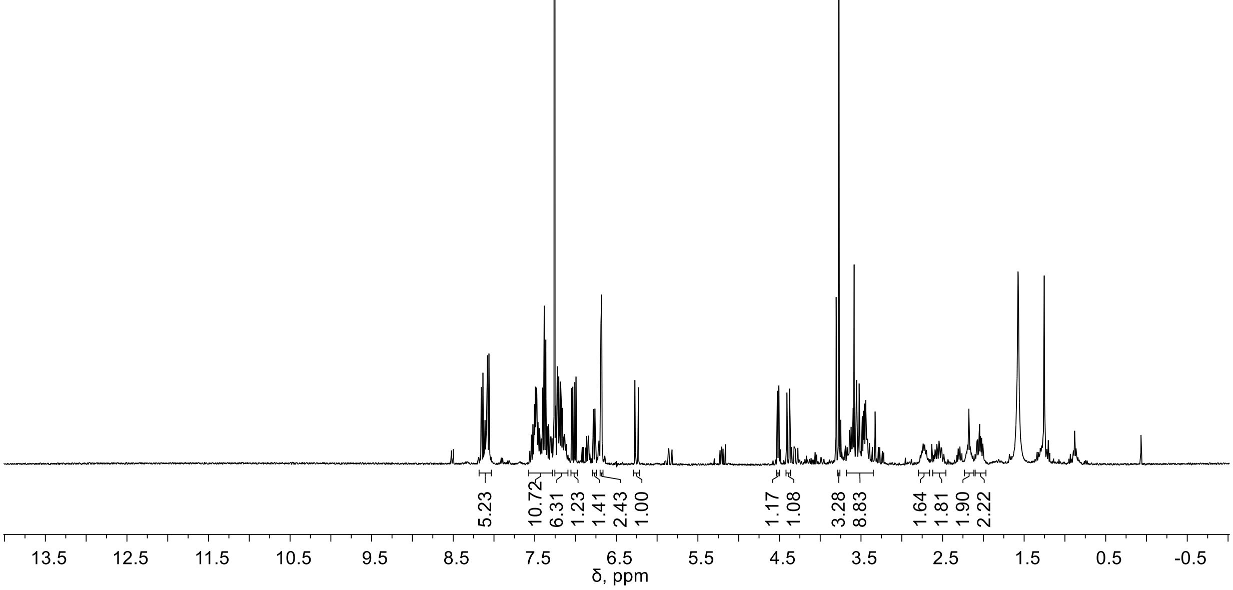

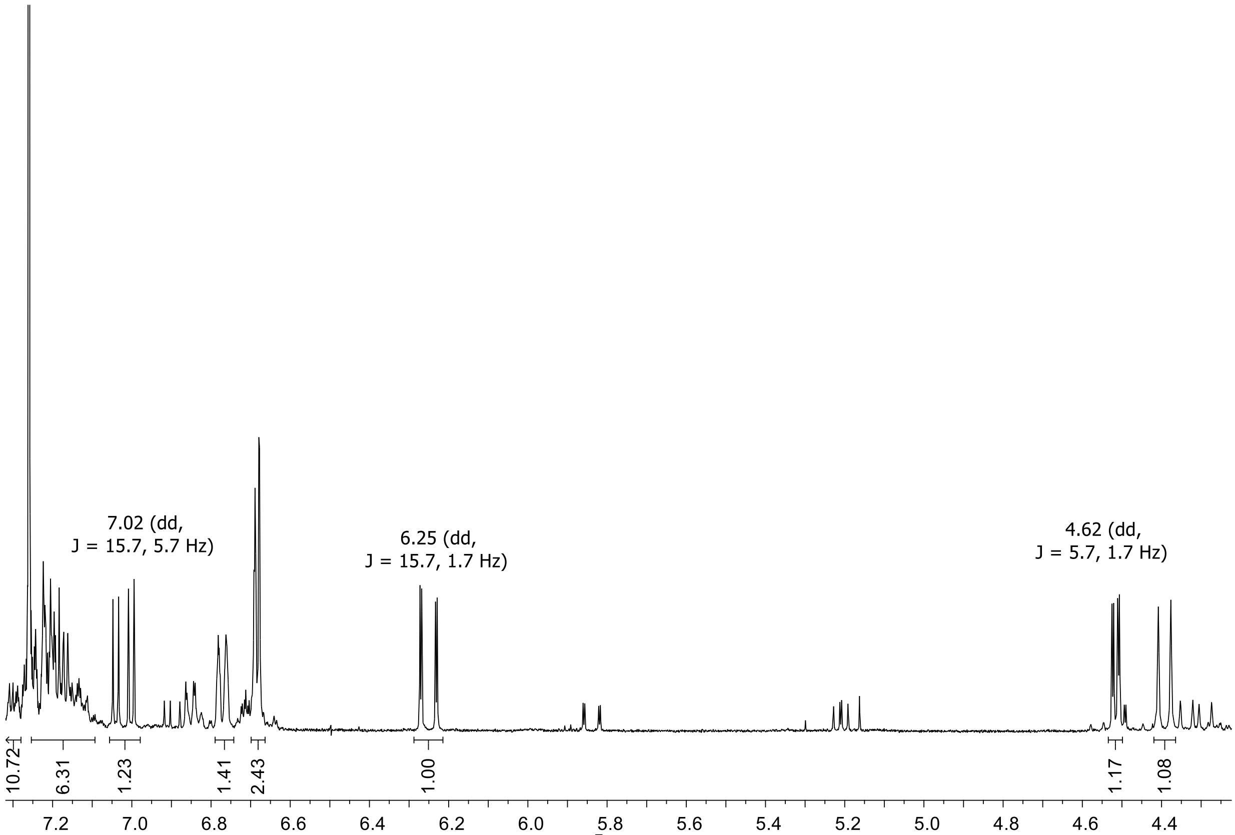

32. Atom numeration and signal assignment in the NMR spectra of complex ***9***

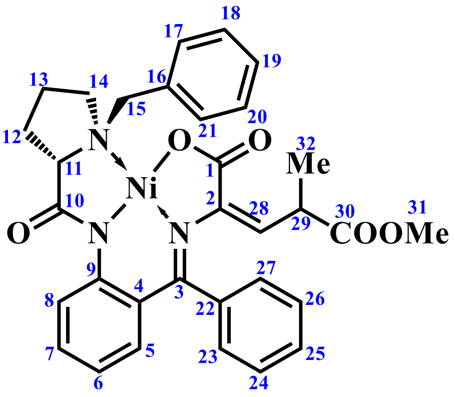

^{1}H NMR (CDCl$_3$ δ, ppm): 8.12-8.07 (m, 3H (H-8,17,21)), 7.47-7.39 (m, 3H (H-23,24,26)), 7.34-7.29 (m, 2H (H-18,20)), 7.18-7.10 (m, 4H (H-7,19,25,27)), 6.89 (dd, ^{3}J = 8.3 Hz, ^{4}J = 1.6 Hz, 1H (H-5)), 6.70 (ddd, ^{3}J = 8.3 Hz, ^{3}J = 7.0 Hz, ^{4}J = 1.2 Hz, 1H (H-6)), 5.02 (d, ^{3}J = 10.3 Hz, 1H (H-28)), 4.36-4.27 (m, 2H (H-15,29)), 3.90-3.77 (m, 1H (H-13)), 3.64 (s, 3H (H-31)), 3.48-3.43 (m, 1H (H-11)), 3.40 (d, ^{3}J = 12.6 Hz, 1H (H-15)), 2.78-2.69 (m, 1H (H-12)), 2.63-2.45 (m, 2H (H-12,14)), 2.25-2.16 (m, 1H (H-13)), 2.11-2.03 (m, 1H (H-14)), 0.74 (d, ^{3}J = 7.1 Hz, 3H (H-32)).

^{13}C NMR (CDCl$_3$ δ, ppm): 180.25 (C-10), 174.26 (C-30), 168.97 (C-1), 167.71 (C-3), 143.46 (C-9), 140.74 (C-2), 135.08 (C-22), 134.09 (C-5), 133.65 (C-16), 132.95 (C-7), 131.49 (C-17,21), 131.35 (C-28), 129.85 (C-24,26), 129.15 (C-25), 129.11 (C-27), 129.07 (C-18,20), 128.97 (C-19), 128.87 (C-23), 127.02 (C-4), 123.67 (C-8), 120.83 (C-6), 70.75 (C-11), 63.04 (C-15), 57.53 (C-14), 52.14 (C-31), 36.16 (C-29), 30.82 (C-12), 24.14 (C-13), 17.28 (C-32).

33. ^{1}H NMR spectrum of complex ***9***

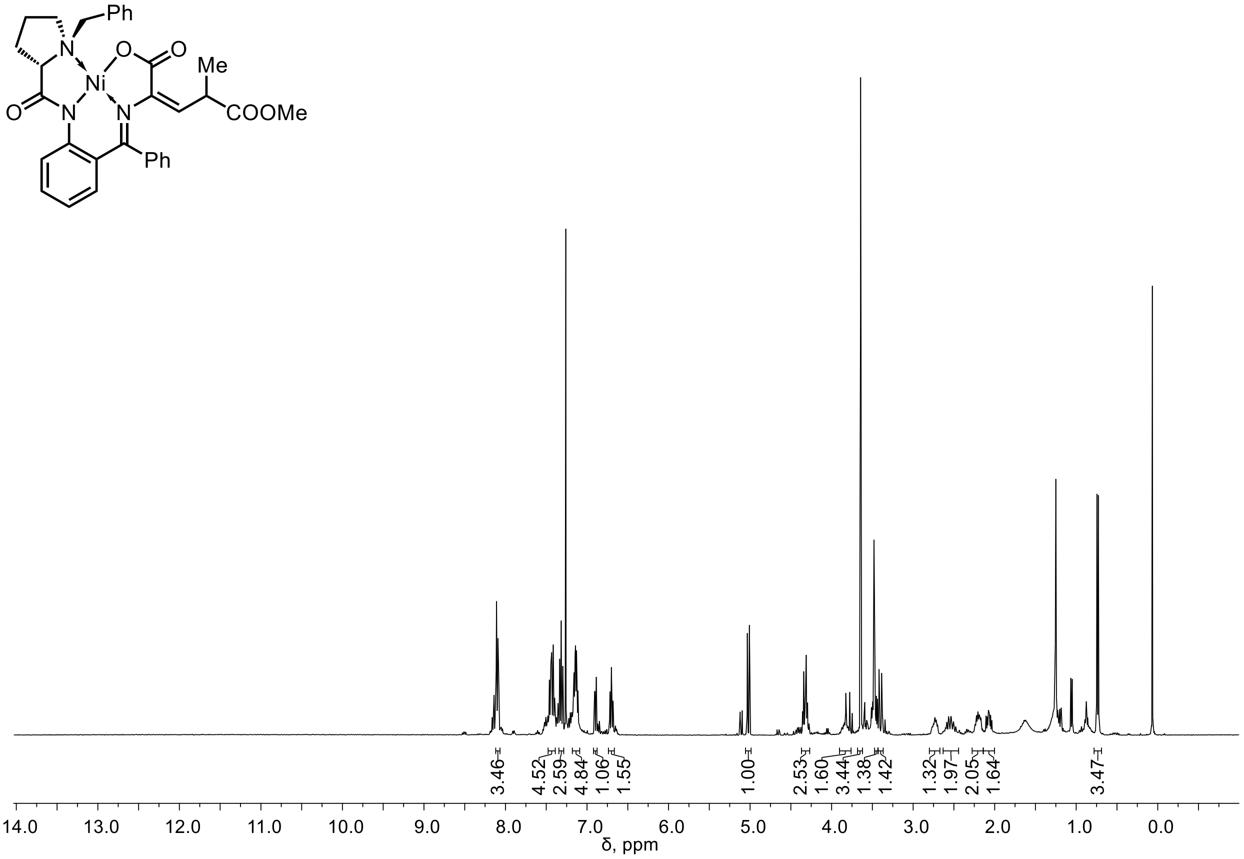

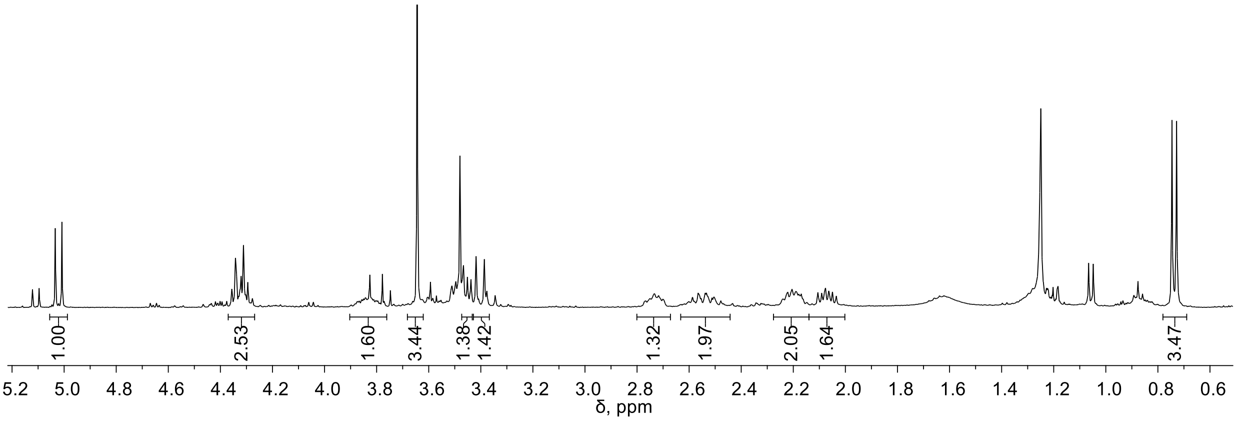

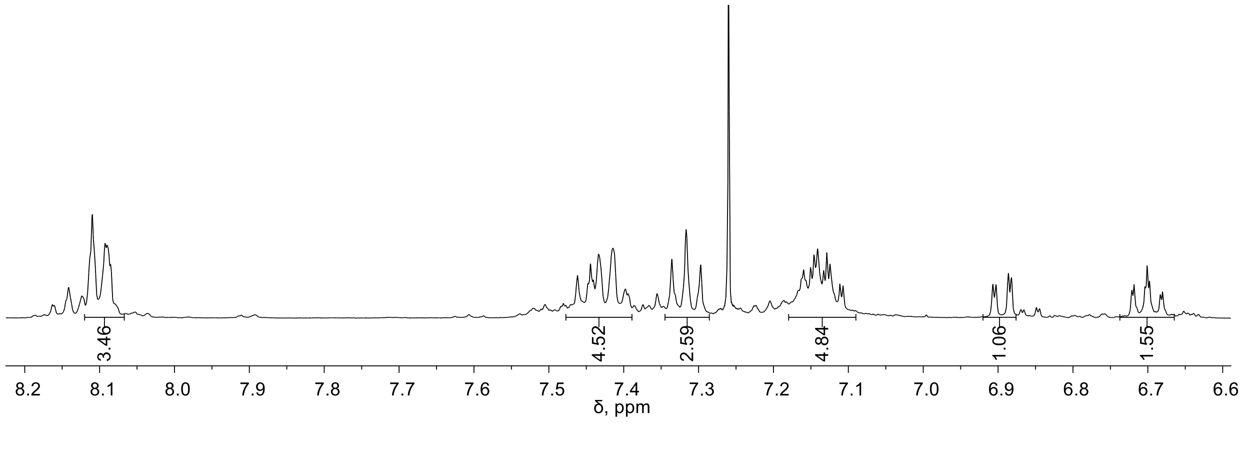

34. ^{13}C NMR spectrum of complex ***9***

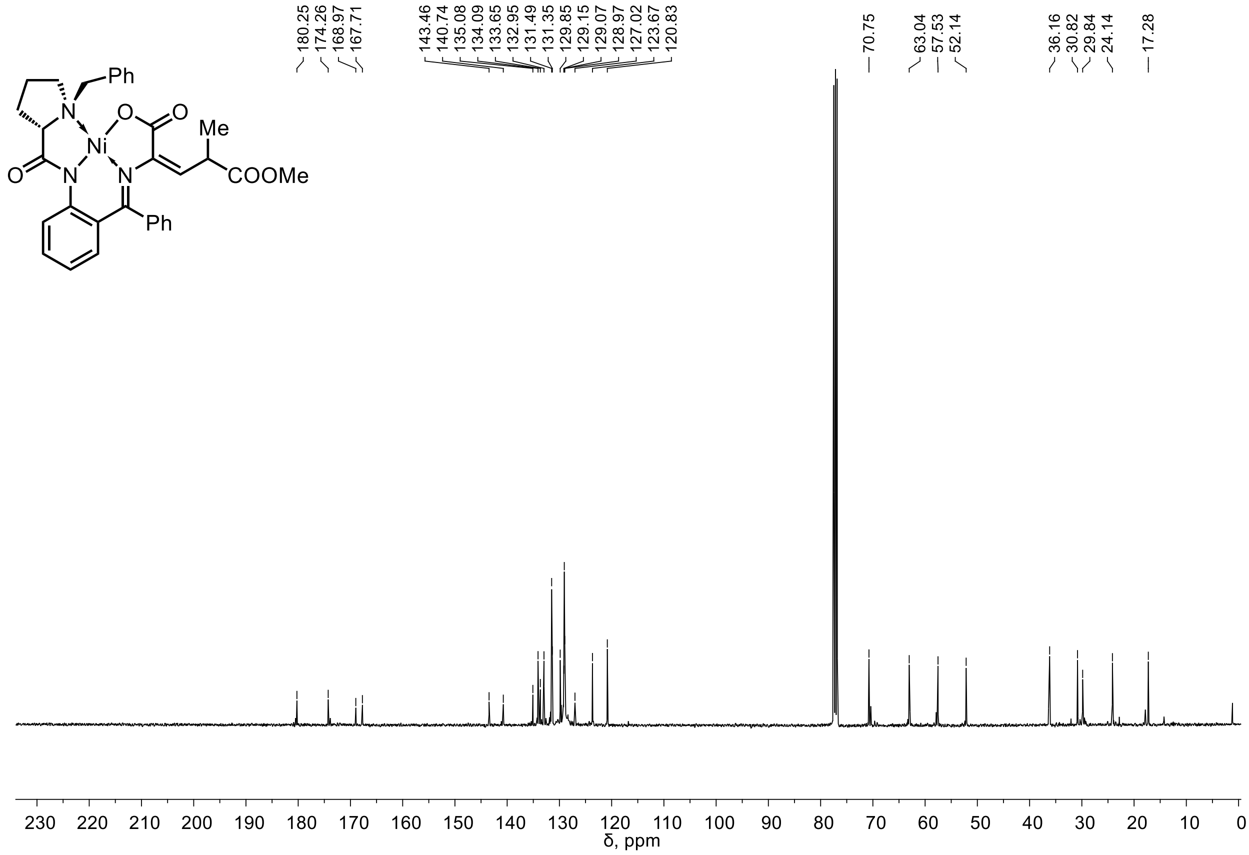

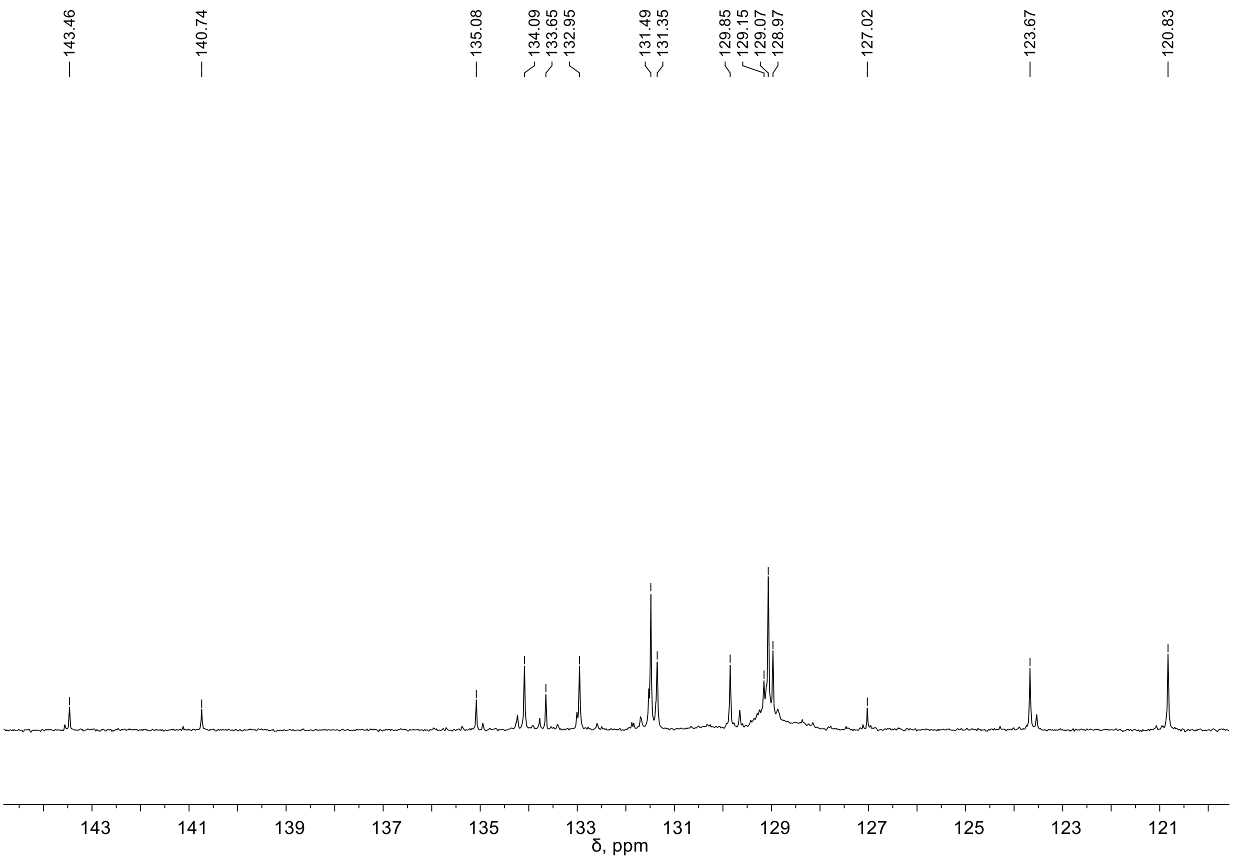

*35. COSY spectrum of complex **9***

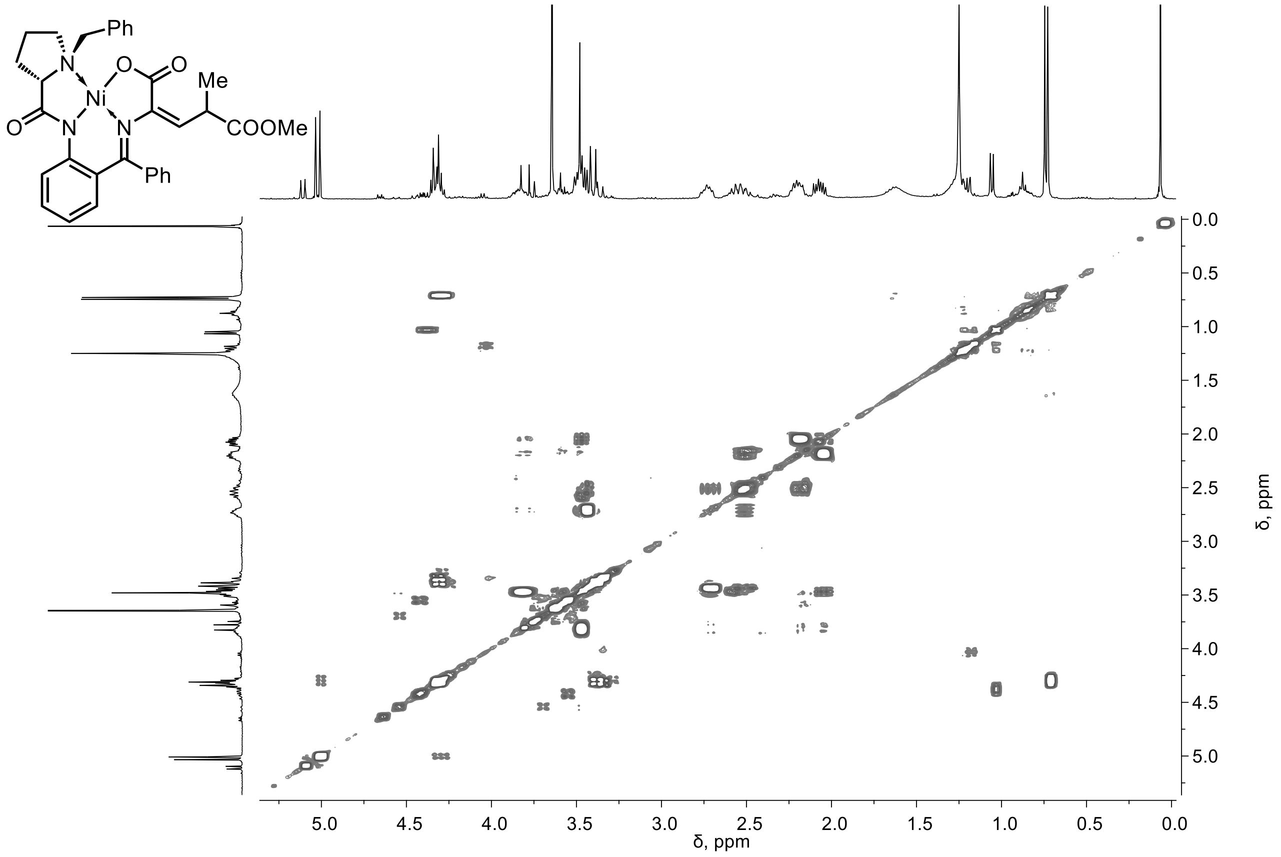

36. HSQC spectrum of complex ***9***

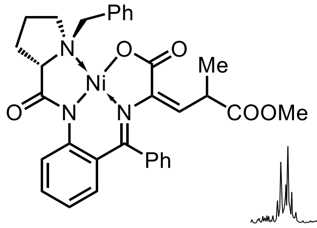

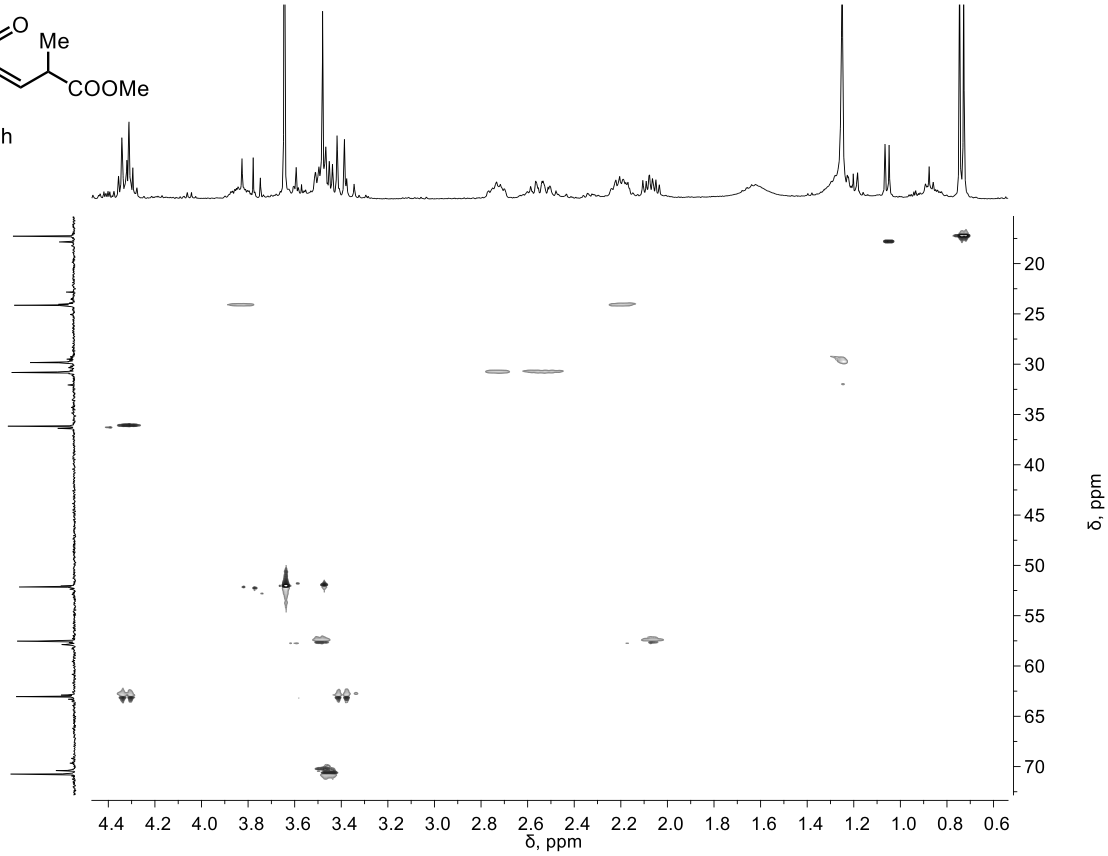

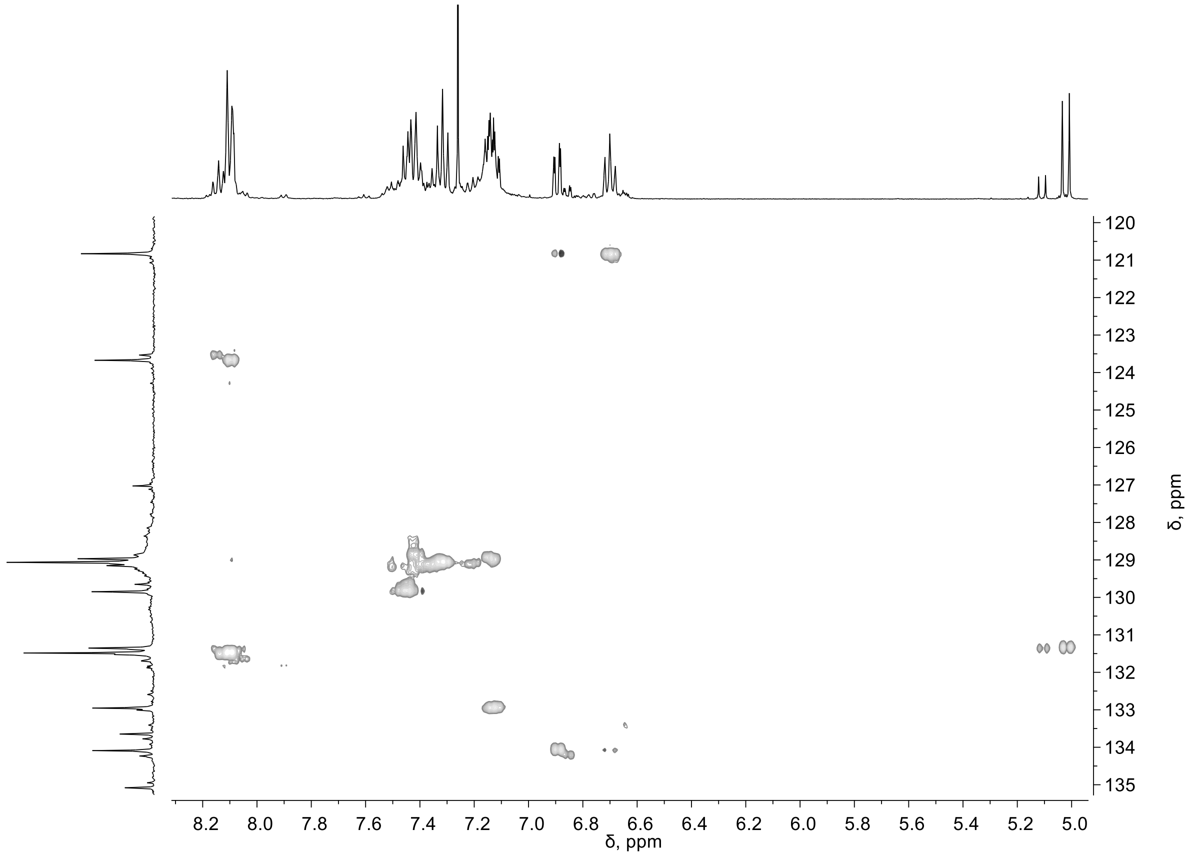

37. HMBC spectrum of complex ***9***

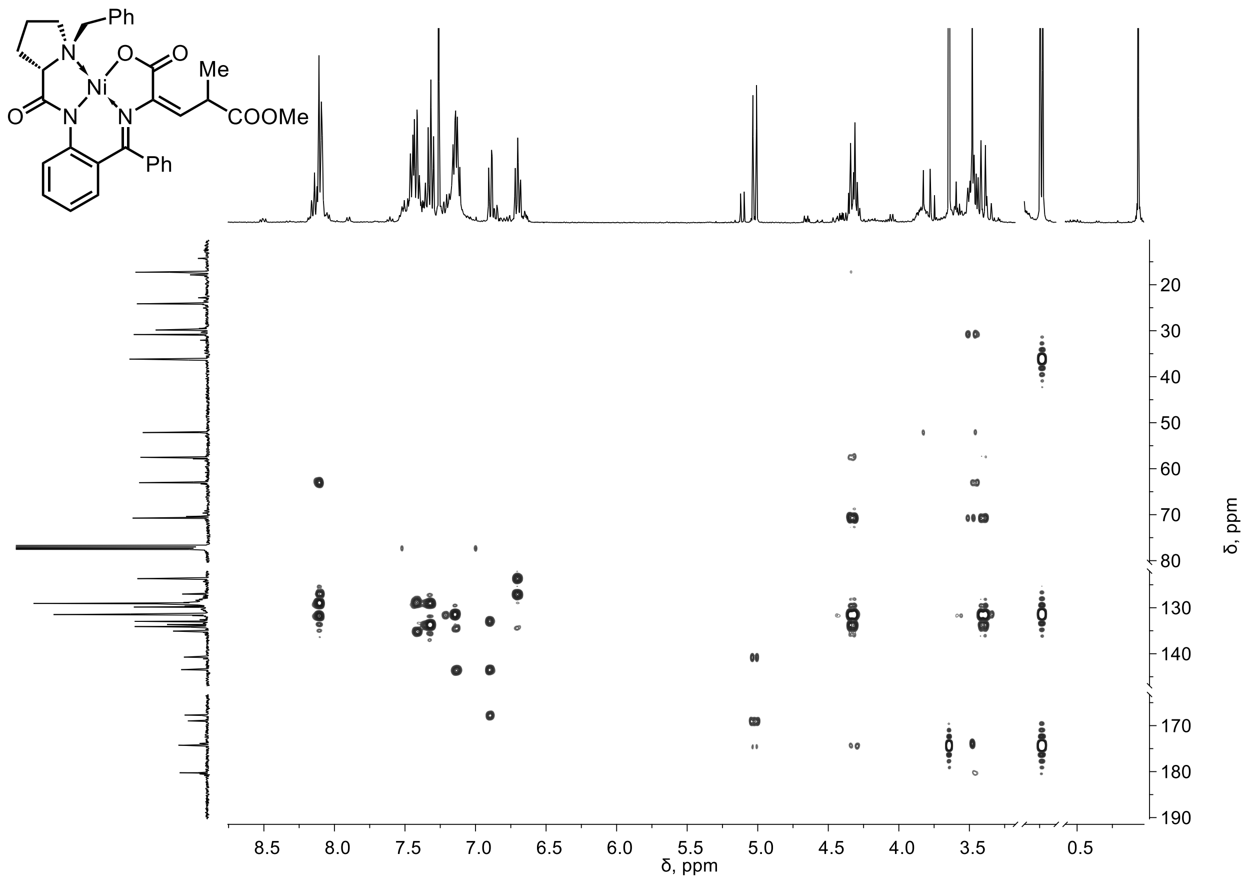

*38. Atom numeration and signal assignment in the NMR spectra of complex (R,S)-**10***

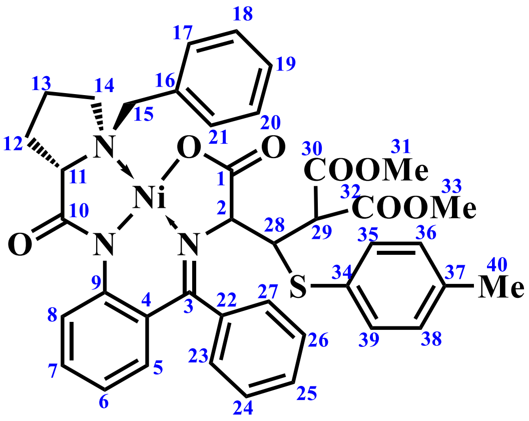

(*R*,*S*)-**10**

^{1}H NMR ($CDCl_3$ δ, ppm): 8.37 (dd, $^{3}J = 8.8$ Hz, $^{4}J = 1.1$ Hz, 1H (H-8)), 8.10-8.05 (m, 2H (H-17,21)), 7.53-7.43 (m, 4H (H-25,26,35,39)), 7.36-7.30 (m, 2H (H-18,20)), 7.25-7.11 (m, 4H (H-7,19,24,27), 6.84-6.80 (m, 2H (H-36,38)), 6.57 (ddd, $^{3}J = 8.3$ Hz, $^{3}J = 7.0$ Hz, $^{4}J = 1.1$ Hz, 1H (H-6)), 6.41 (dd, $^{3}J = 8.3$ Hz, $^{4}J = 1.6$ Hz, 1H (H-5)), 5.66-5.62 (m, 1H (H-23)), 4.61 (d, $^{3}J = 5.6$ Hz, 1H (H-2)), 4.48 (d, $^{2}J = 12.6$ Hz, 1H (H-15)), 4.06 (d, $^{3}J = 11.4$ Hz, 1H (H-29)), 3.89-3.78 (m, 1H (H-13)), 3.74 (s, 3H (H-31)), 3.71-3.64 (m, 1H (H-14)), 3.61 (d, $^{2}J = 12.6$ Hz, 1H (H-15)), 3.52-3.48 (m, 4H (H-11,33)), 3.41 (dd, $^{3}J = 11.4$ Hz, $^{3}J = 5.6$ Hz, 1H (H-28)), 2.88-2.78 (m, 1H (H-12)), 2.54-2.41 (m, 1H (H-12)), 2.12 (s, 3H (H-40)), 2.15-2.01 (m, 2H (H-13,14)).

^{13}C NMR ($CDCl_3$ δ, ppm): 180.58 (C-10), 176.57 (C-1), 172.40 (C-3), 168.56 (C-30), 166.63 (C-32), 143.01 (C-9), 138.83 (C-37), 134.28 (C-35,39), 133.71 (C-22), 133.63 (C-5), 133.53 (C-16), 132.44 (C-7), 131.76 (C-17,21), 131.20 (C-34), 129.91 (C-36,38), 129.81 (C-25), 129.07 (C-26), 128.90 (C-19), 128.84 (C-18,20), 128.66 (C-24), 127.37 (C-23), 127.06 (C-27), 125.88 (C-4), 123.58 (C-8), 120.43 (C-6), 70.67 (C-11), 70.06 (C-2), 63.55 (C-15), 57.40 (C-14), 55.26 (C-29), 52.83 (C-31), 52.71 (C-33), 52.24 (C-28), 30.75 (C-12), 23.44 (C-13), 21.03 (C-40).

(*R*,*R*)-**10**

^{1}H NMR ($CDCl_3$ δ, ppm): 8.38 (dd, $^{3}J = 8.8$ Hz, $^{4}J = 1.1$ Hz, 1H (H-8)), 8.01-7.96 (m, 2H (H-17,21)), 7.54-7.43 (m, 4H (H-24,25,26,27)), 7.28-7.17 (m, 5H (H-18,20,23,35,39)), 7.14 (ddd, $^{3}J = 8.8$ Hz, $^{3}J = 6.9$ Hz, $^{4}J = 1.7$ Hz, 1H (H-7)), 7.10-7.05 (m, 1H (H-19)), 6.99-6.95 (m, 2H (H-36,38)), 6.77 (dd, $^{3}J = 8.3$ Hz, $^{4}J = 1.7$ Hz, 1H (H-5)), 6.69 (ddd, $^{3}J = 8.3$ Hz, $^{3}J = 6.9$ Hz, $^{4}J = 1.1$ Hz, 1H (H-6)), 4.78 (dd, $^{3}J = 9.7$ Hz, $^{3}J = 4.4$ Hz, 1H (H-28)), 4.47 (d, $^{3}J = 4.4$ Hz, 1H (H-29)), 4.28 (d, $^{2}J = 12.6$ Hz, 1H (H-15)), 4.13 (d, $^{3}J = 9.7$ Hz, 1H (H-2)), 3.72 (s, 3H (H-31)), 3.78 (s, 3H (H-33)), 3.38 (d, $^{2}J = 12.6$ Hz, 1H (H-15)), 3.35-3.28 (m, 2H (H-11,14)), 3.23-3.07 (m, 1H (H-13)), 2.28 (s, 3H (H-40)), 2.26-2.16 (m, 1H (H-12)), 2.15-2.06 (m, 1H (H-12)), 2.01-1.90 (m, 2H (H-13,14)).

^{13}C NMR ($CDCl_3$ δ, ppm): 179.65 (C-10), 176.80 (C-1), 173.75 (C-3), 168.48 (C-30), 167.29 (C-32), 143.28 (C-9), 138.58 (C-37), 134.61 (C-5), 134.41 (C-22), 133.63 (C-35,39), 133.55 (C-16), 133.07 (C-7), 132.43 (C-34), 131.41 (C-17,21), 130.17 (C-36,38), 128.91 (C-18,20), 128.78 (C-19), 131.78, 129.67,128.69, 128.56, 127.87 (C-23,24,25,26,27), 126.06 (C-4), 122.95 (C-8), 120.57 (C-6), 73.12 (C-2), 70.43 (C-11), 63.19 (C-15), 57.92 (C-28), 57.33 (C-14), 54.26 (C-29), 53.07 (C-31), 52.46 (C-33), 29.94 (C-12), 23.77 (C-13), 21.24 (C-40).

*39. ^{1}H NMR spectrum of complex (R,S)-**10***

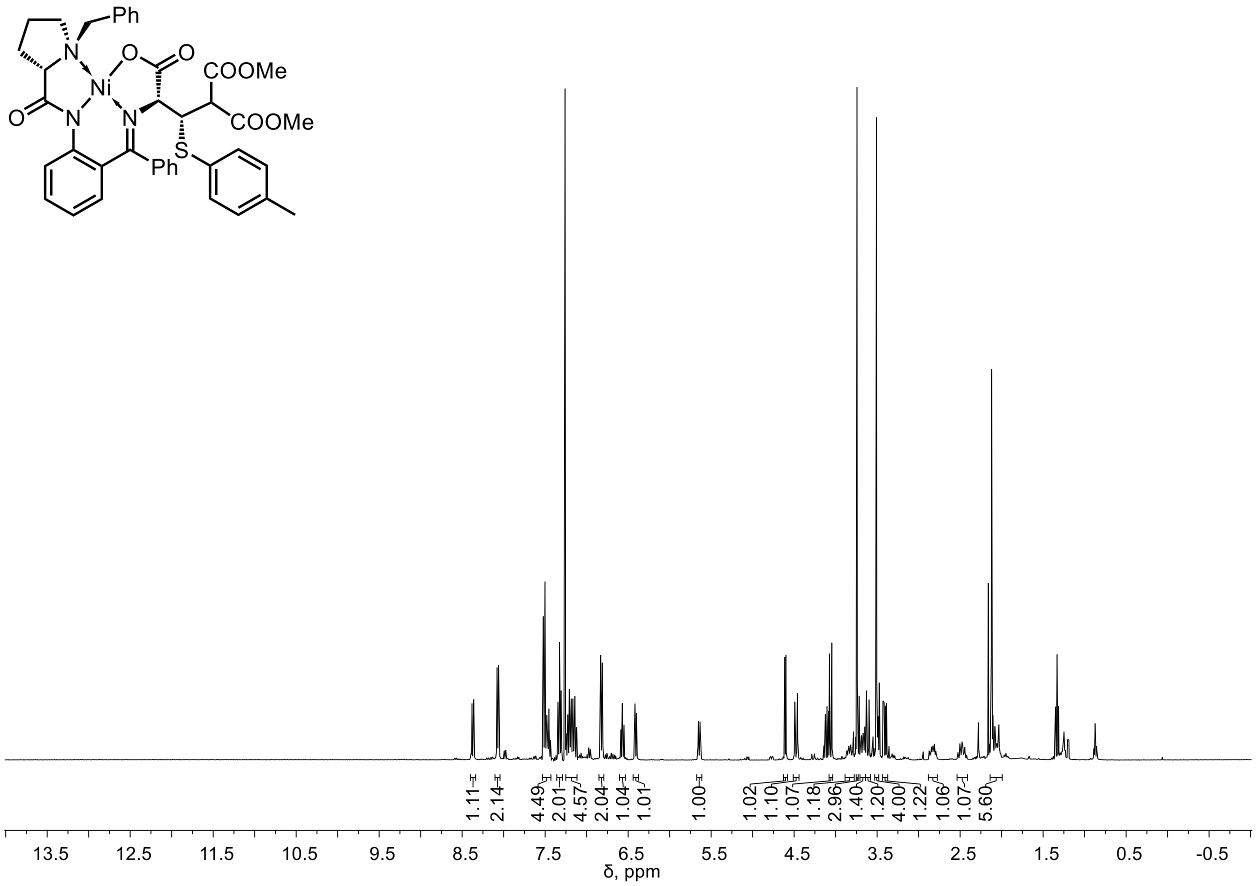

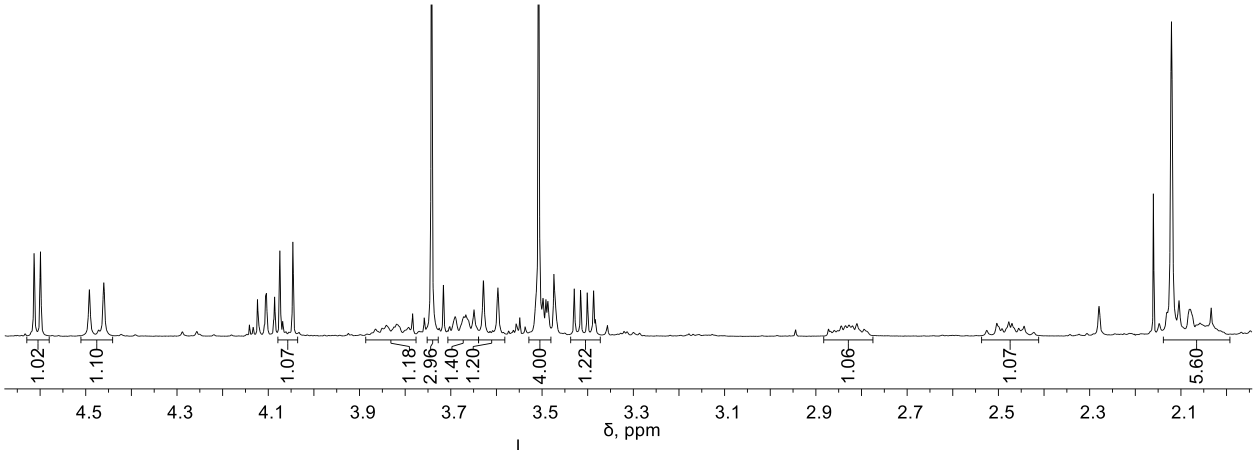

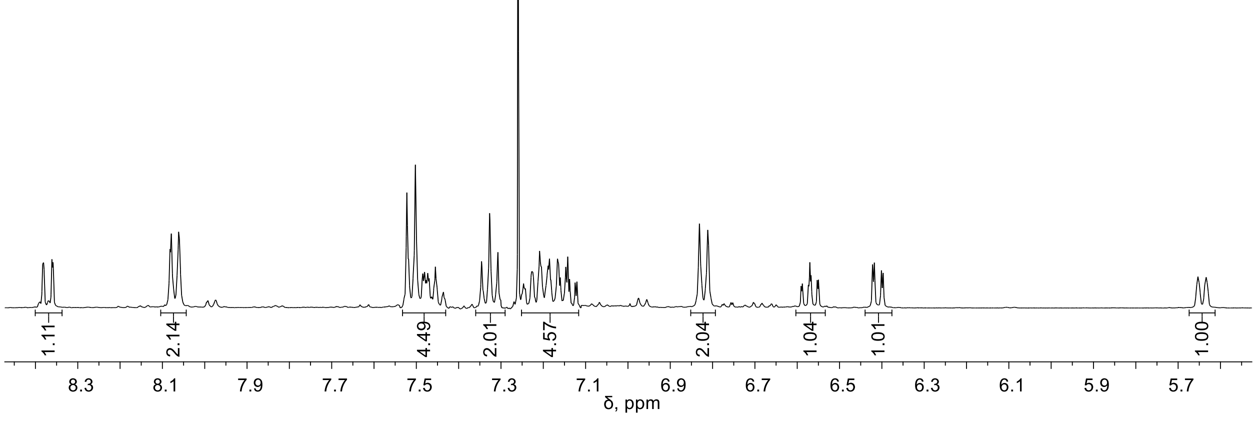

*40. ^{13}C NMR spectrum of complex (R,S)-**10***

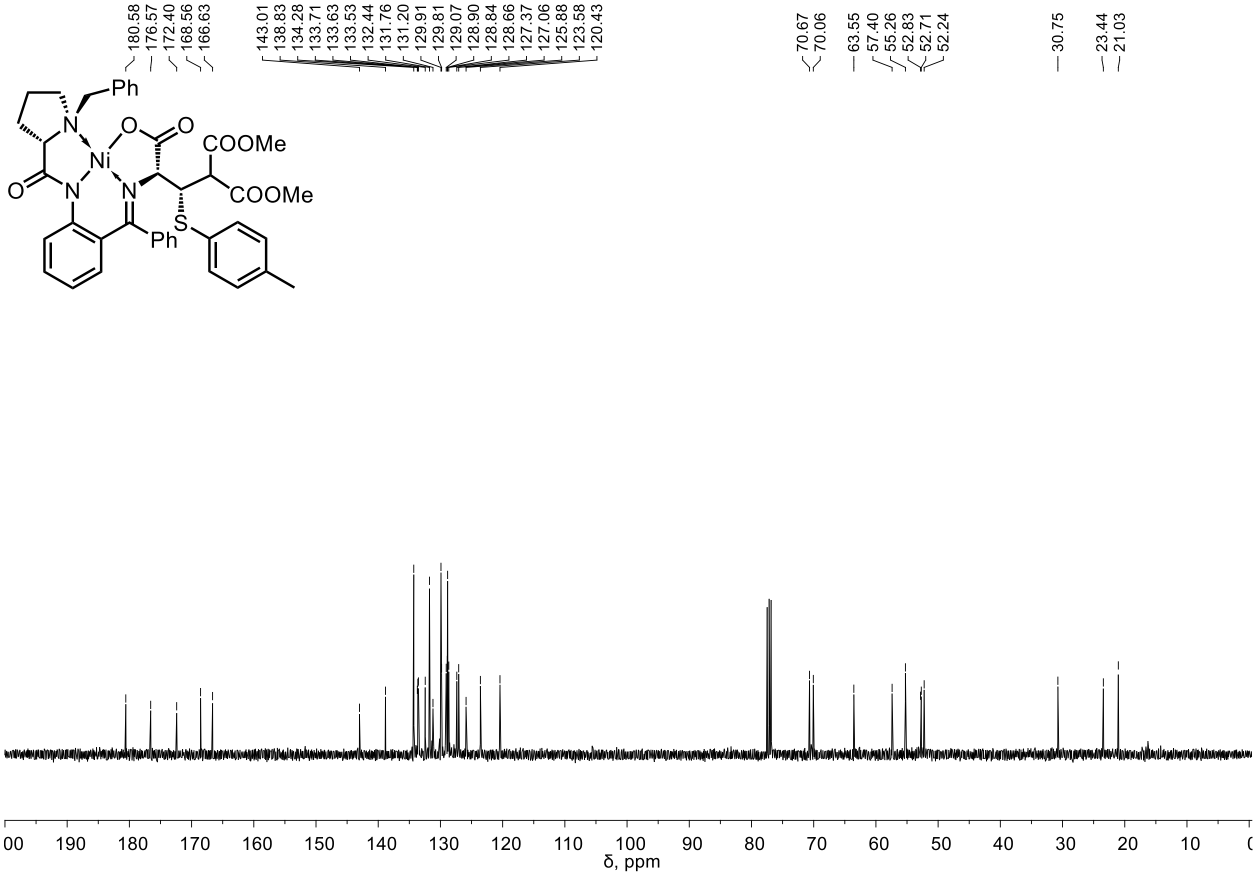

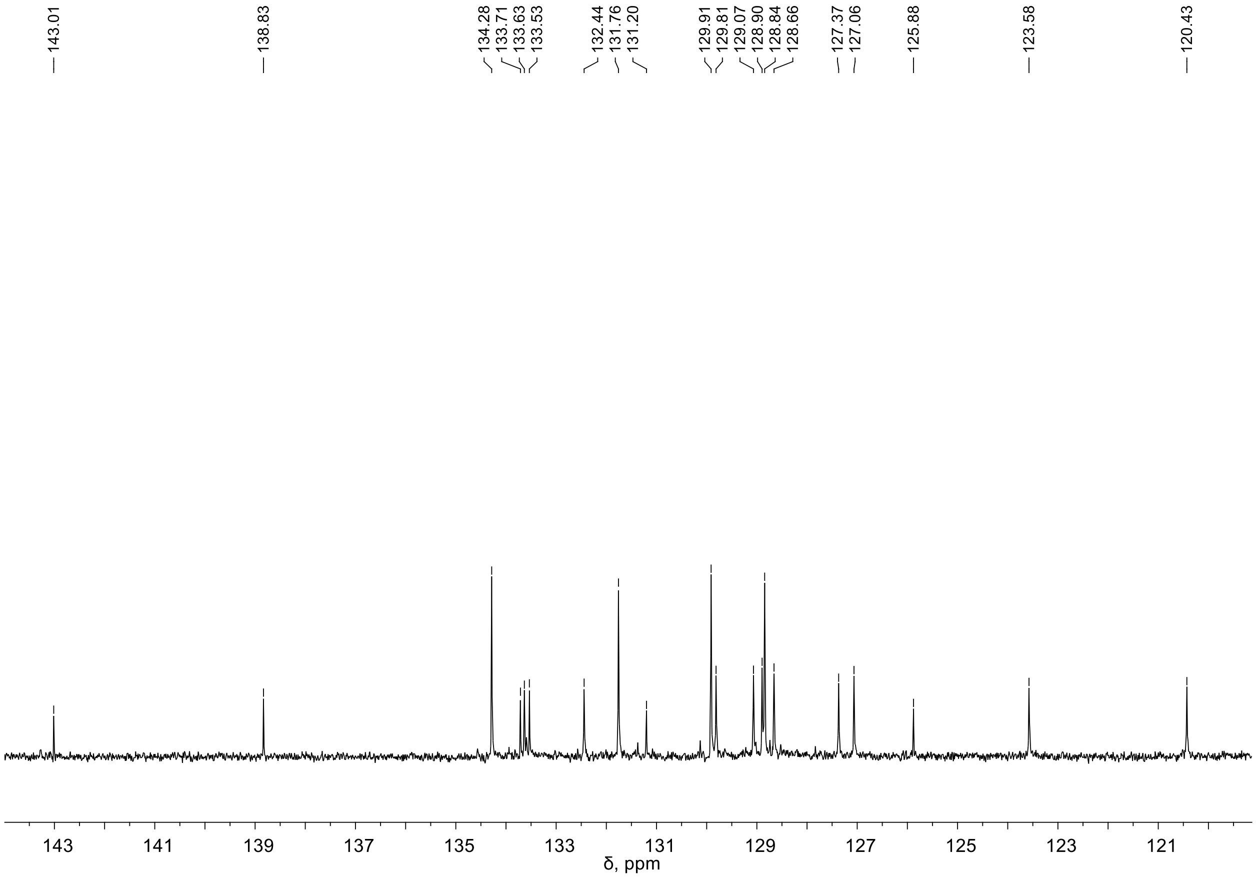

*41. COSY spectrum of complex (R,S)-**10***

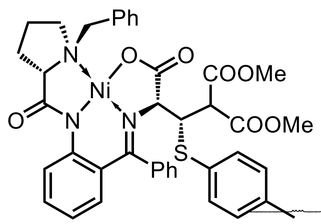

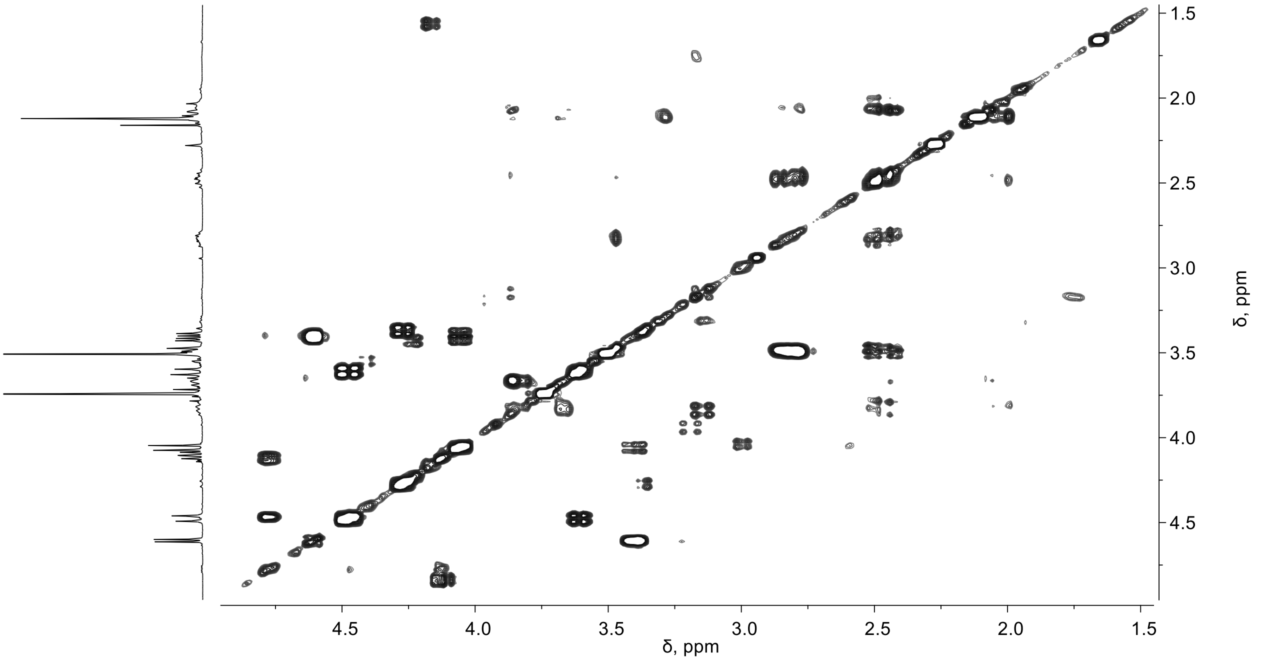

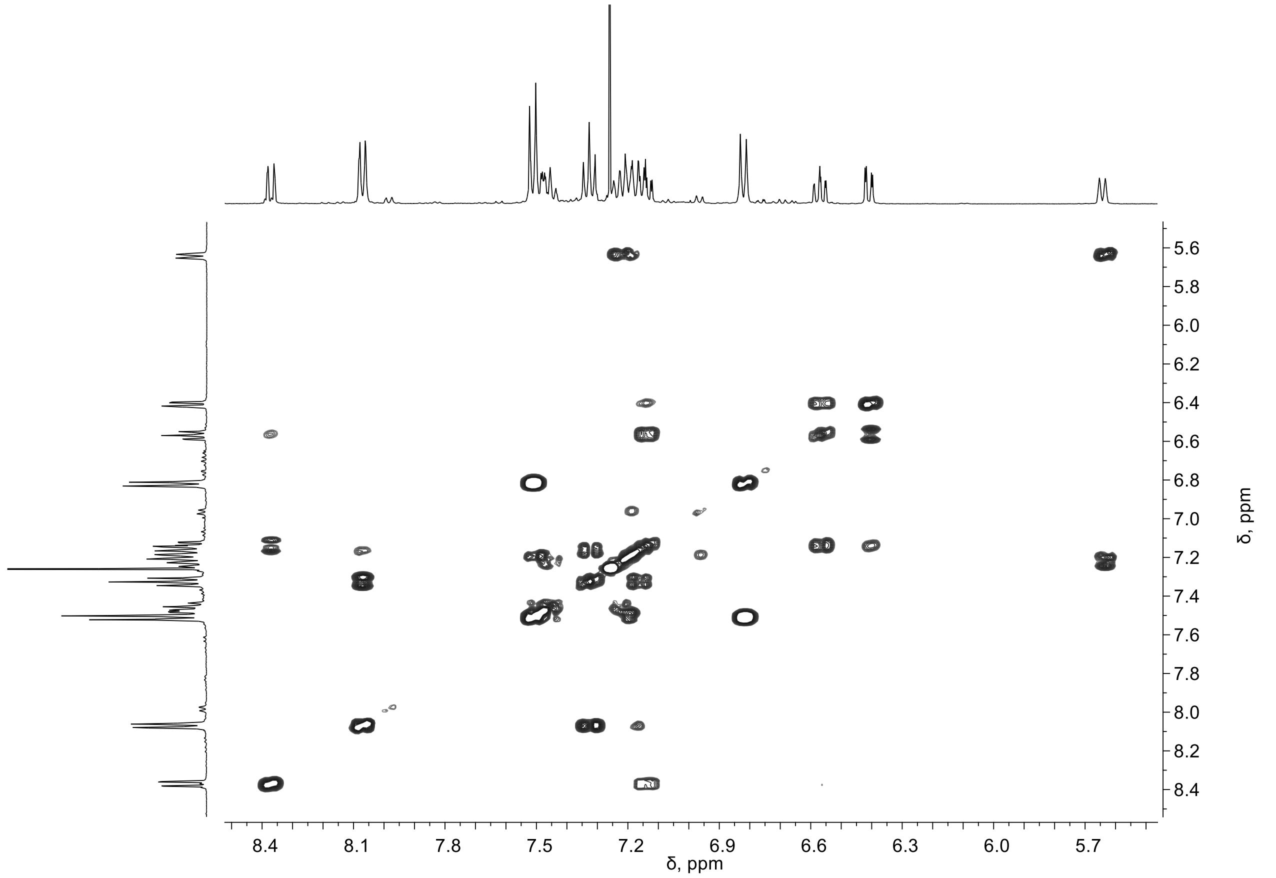

*42. HSQC spectrum of complex (R,S)-**10***

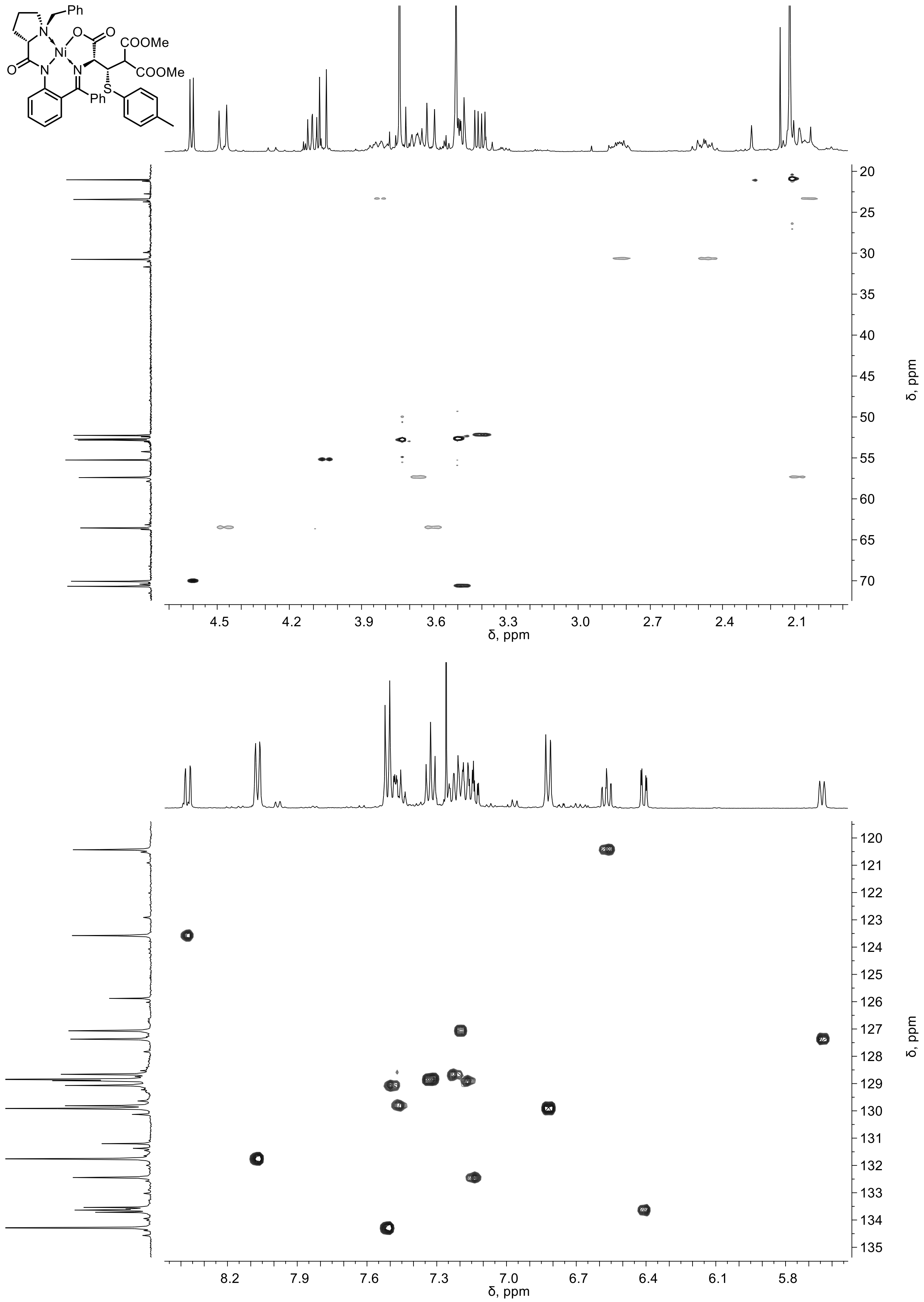

*43. HMBC spectrum of complex (R,S)-**10***

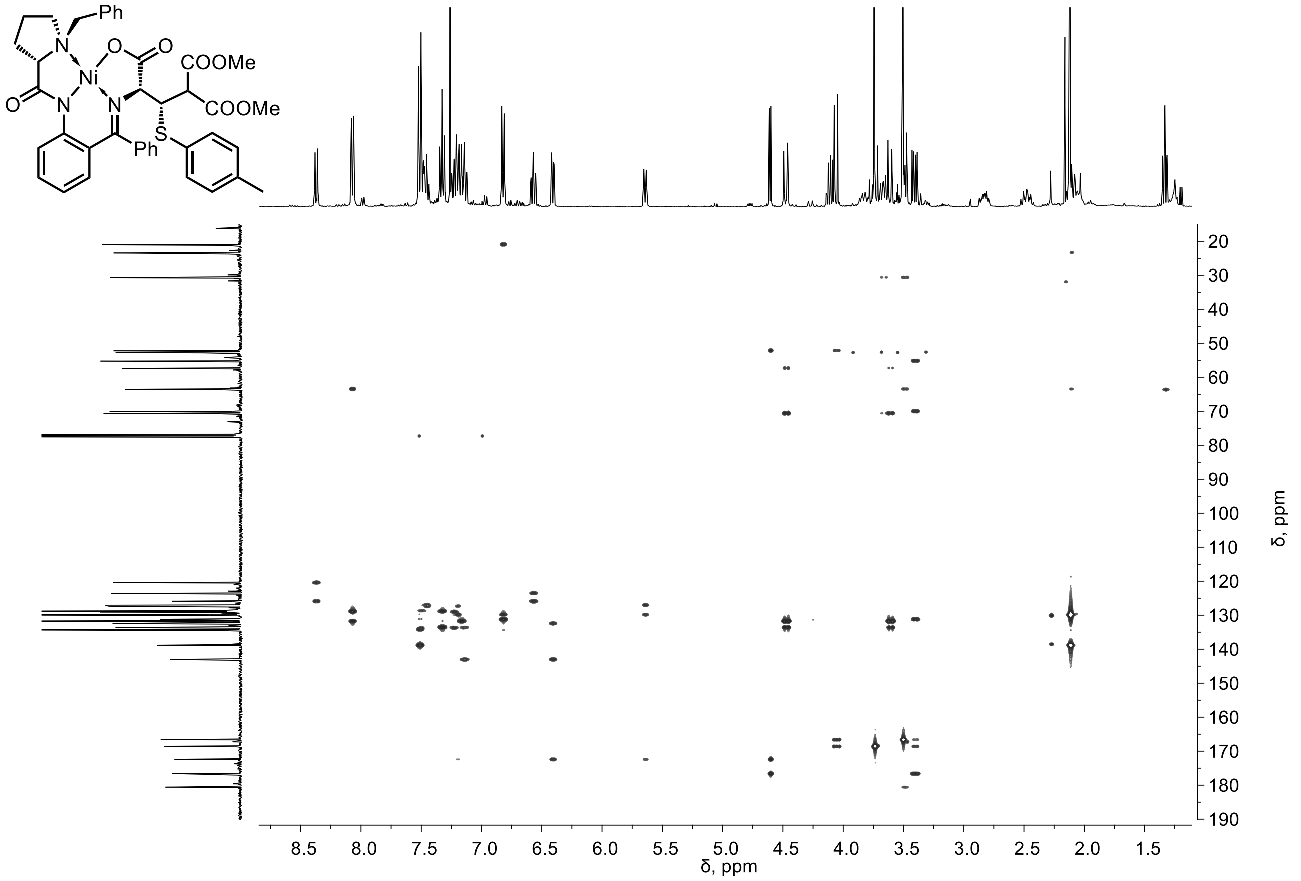

*44. NOESY spectrum of complex (R,S)-**10***

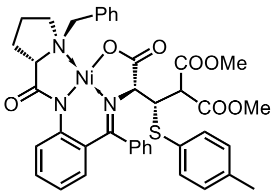

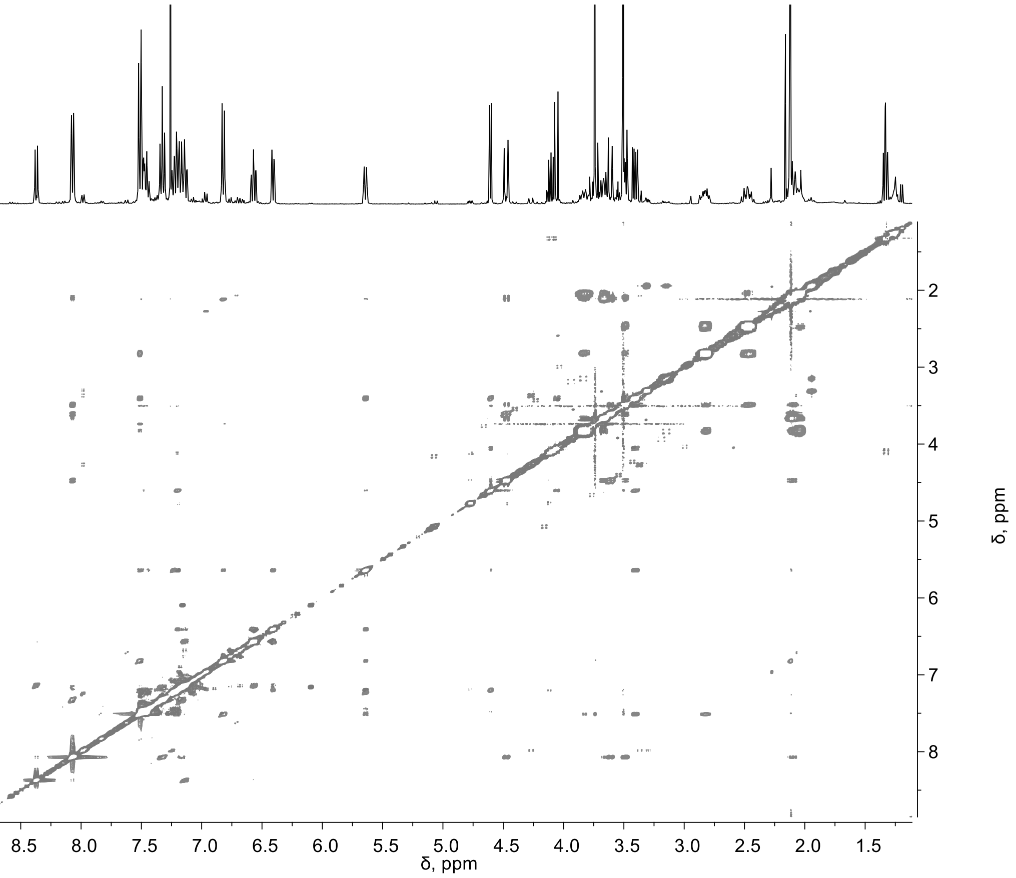

*45. ^{1}H NMR spectrum of complex (R,R)-**10***

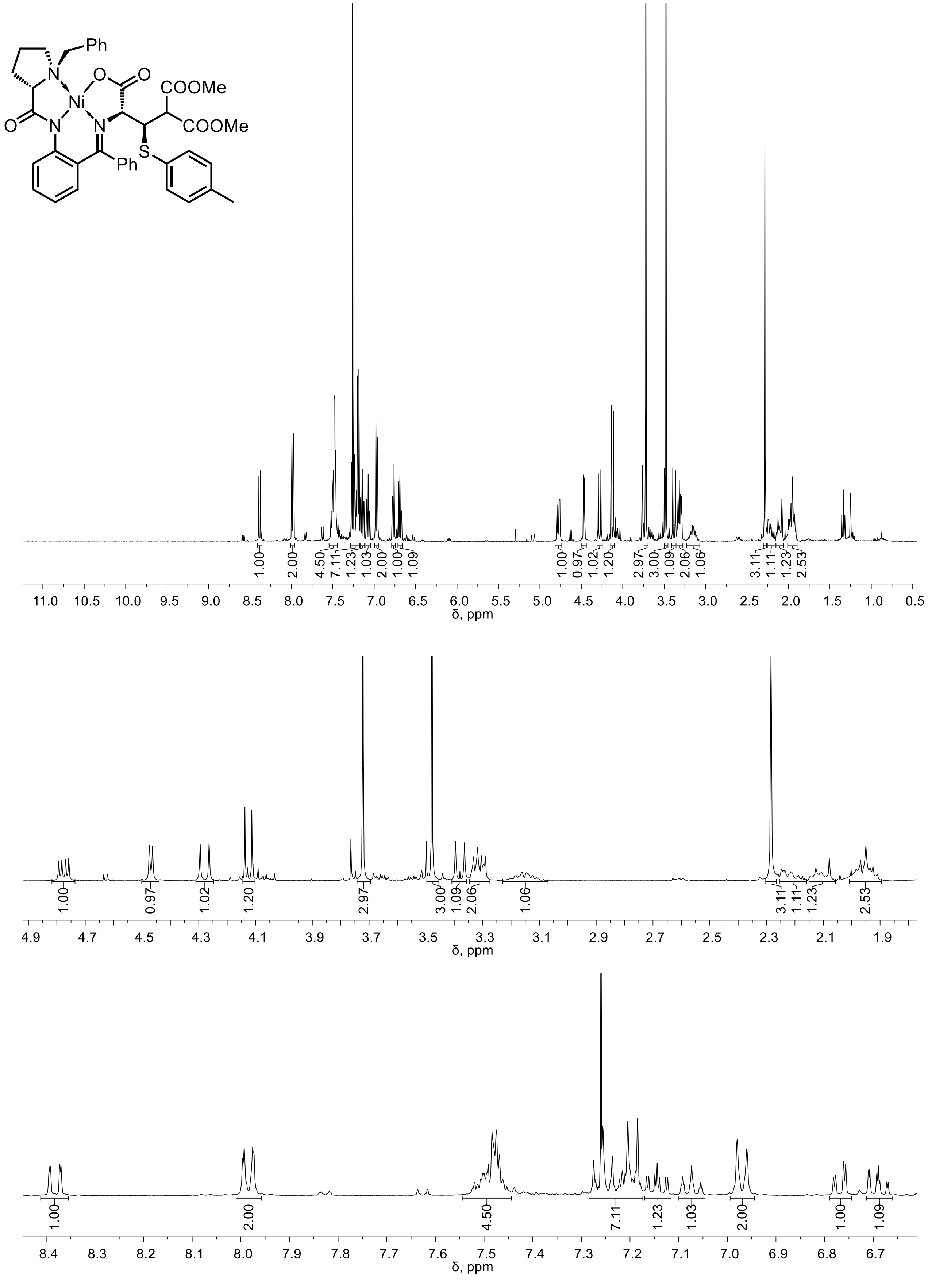

*46. ^{13}C NMR spectrum of complex (R,R)-**10***

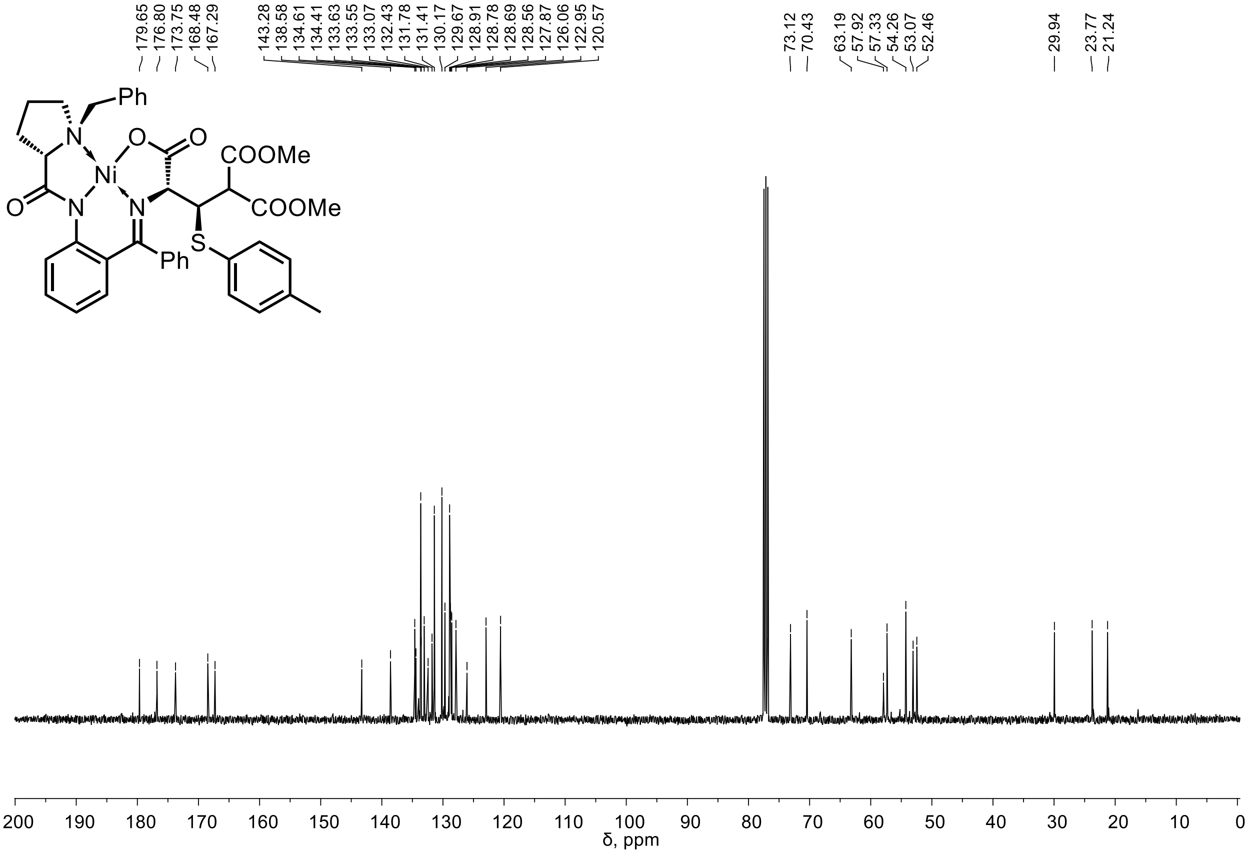

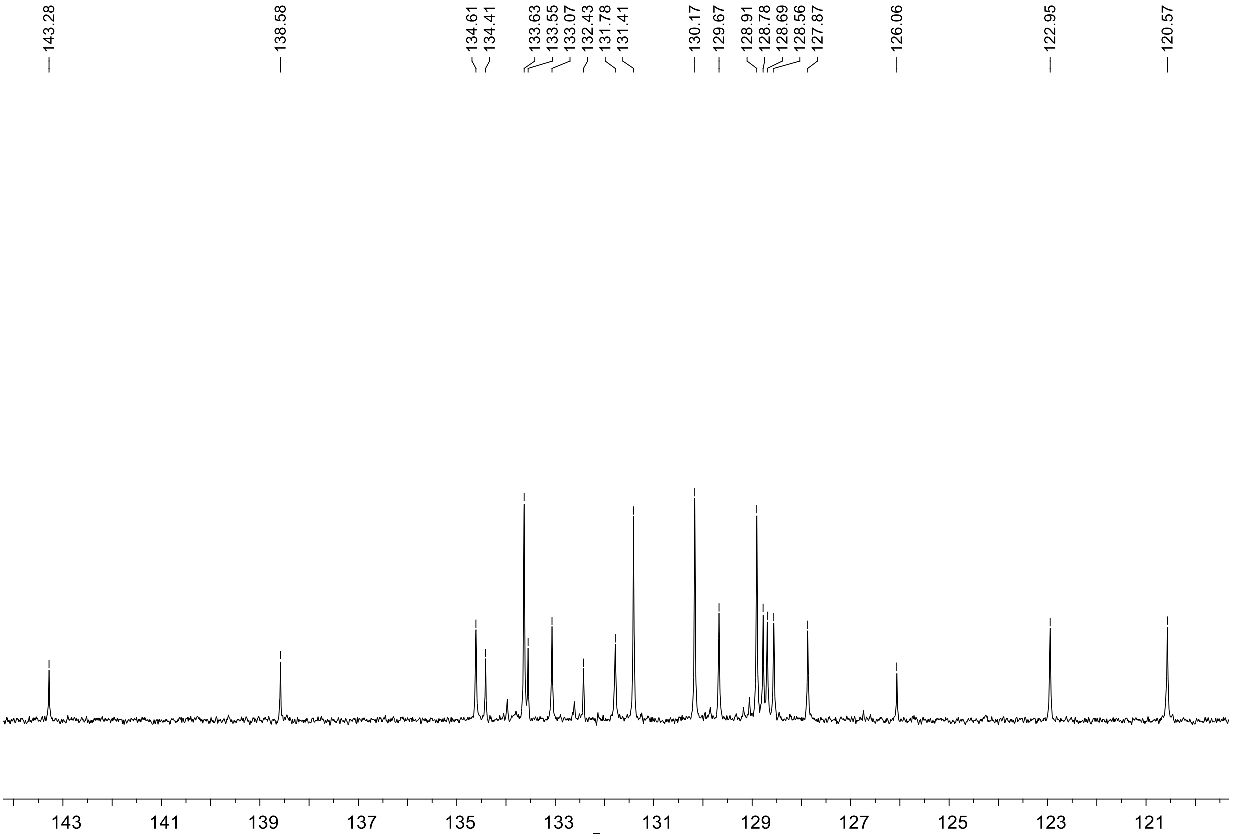

*47. COSY spectrum of complex (R,R)-**10***

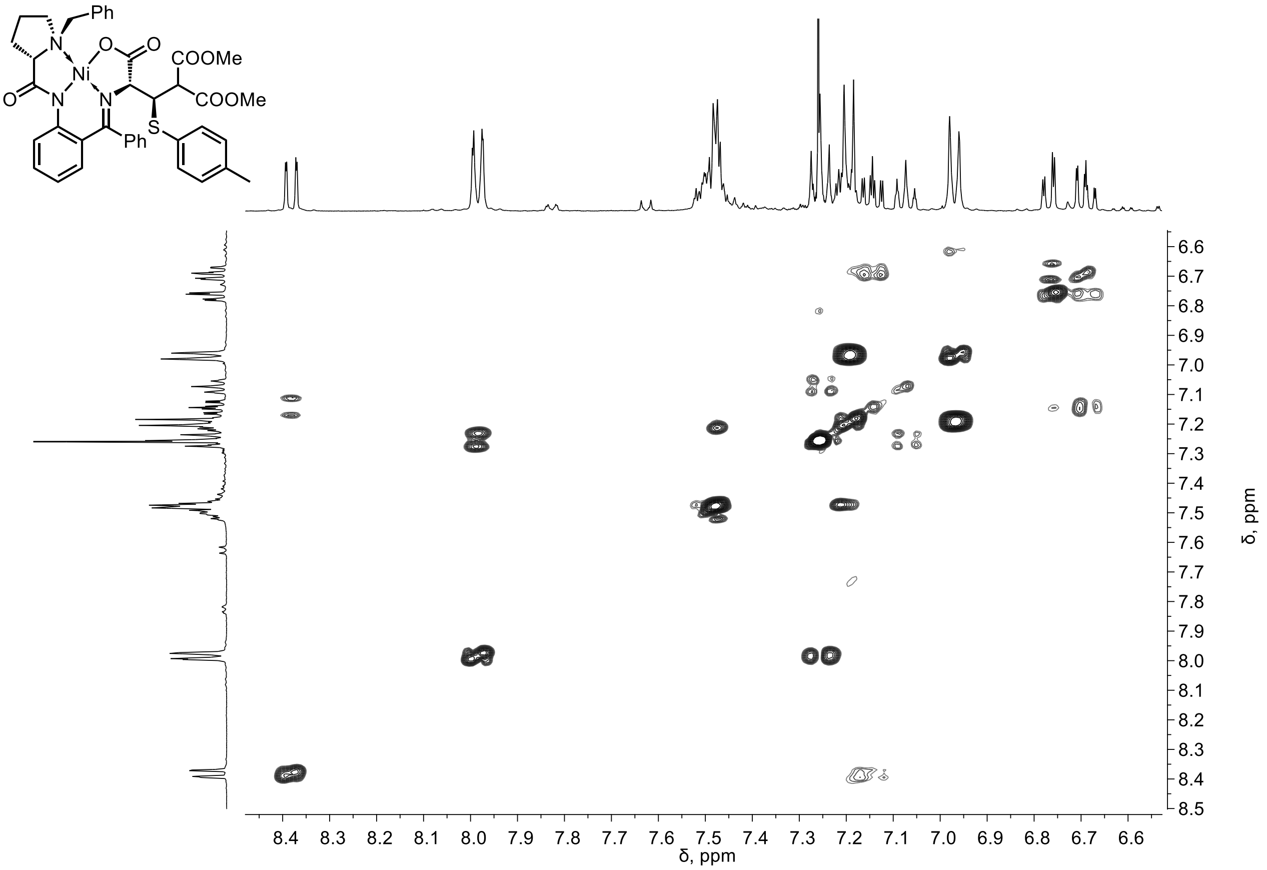

*48. HSQC spectrum of complex (R,R)-**10***

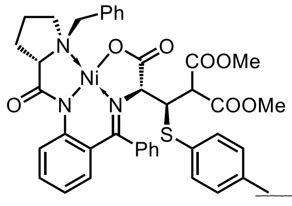

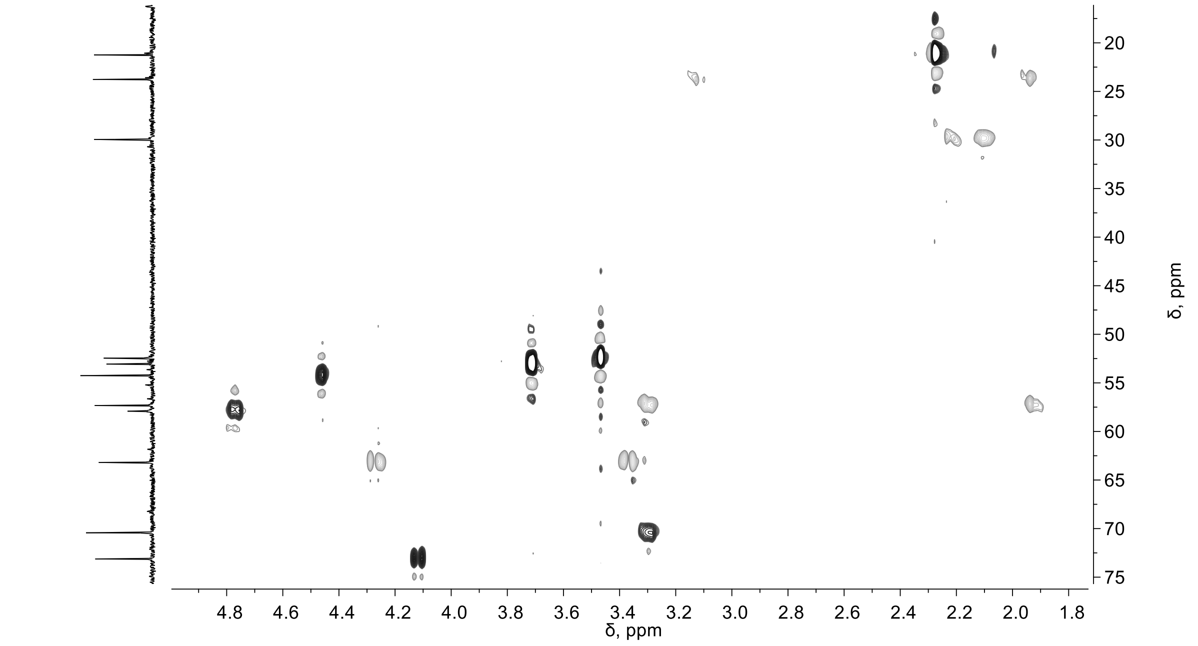

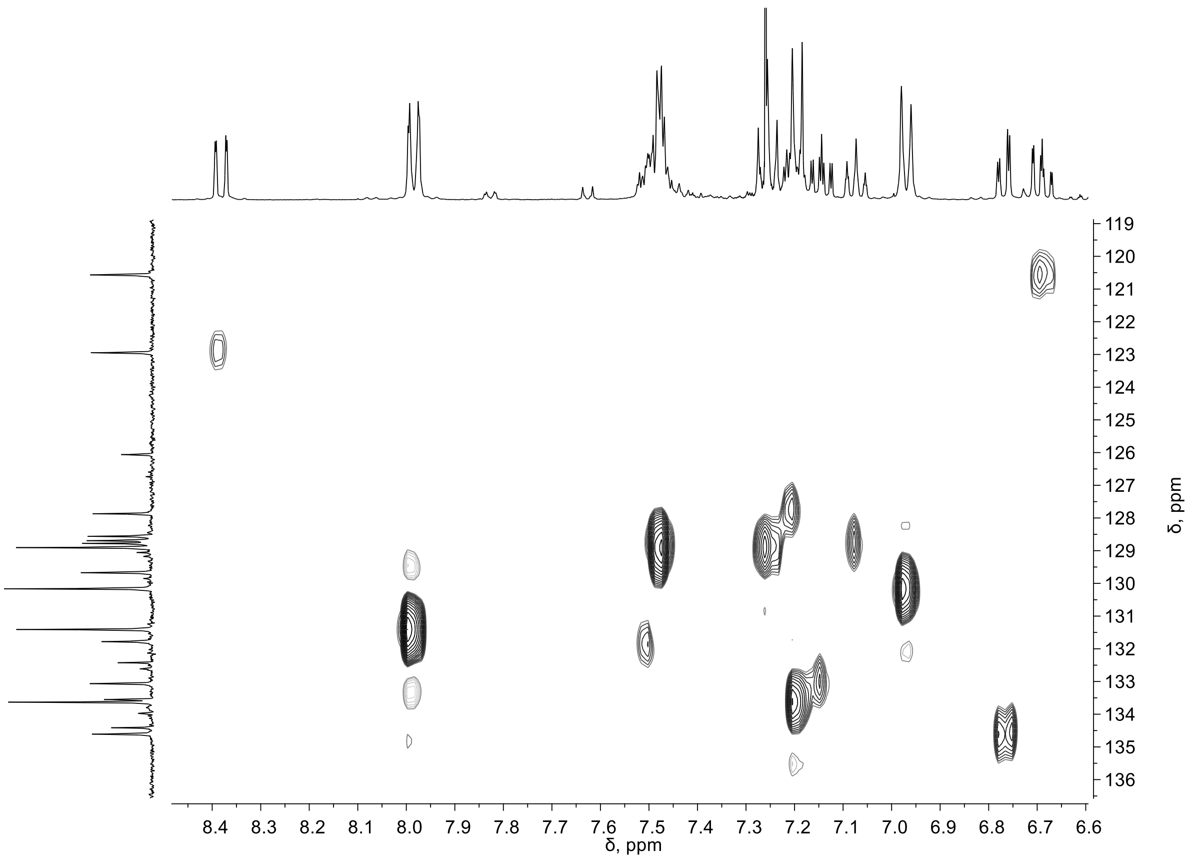

*49. HMBC spectrum of complex (R,R)-**10***

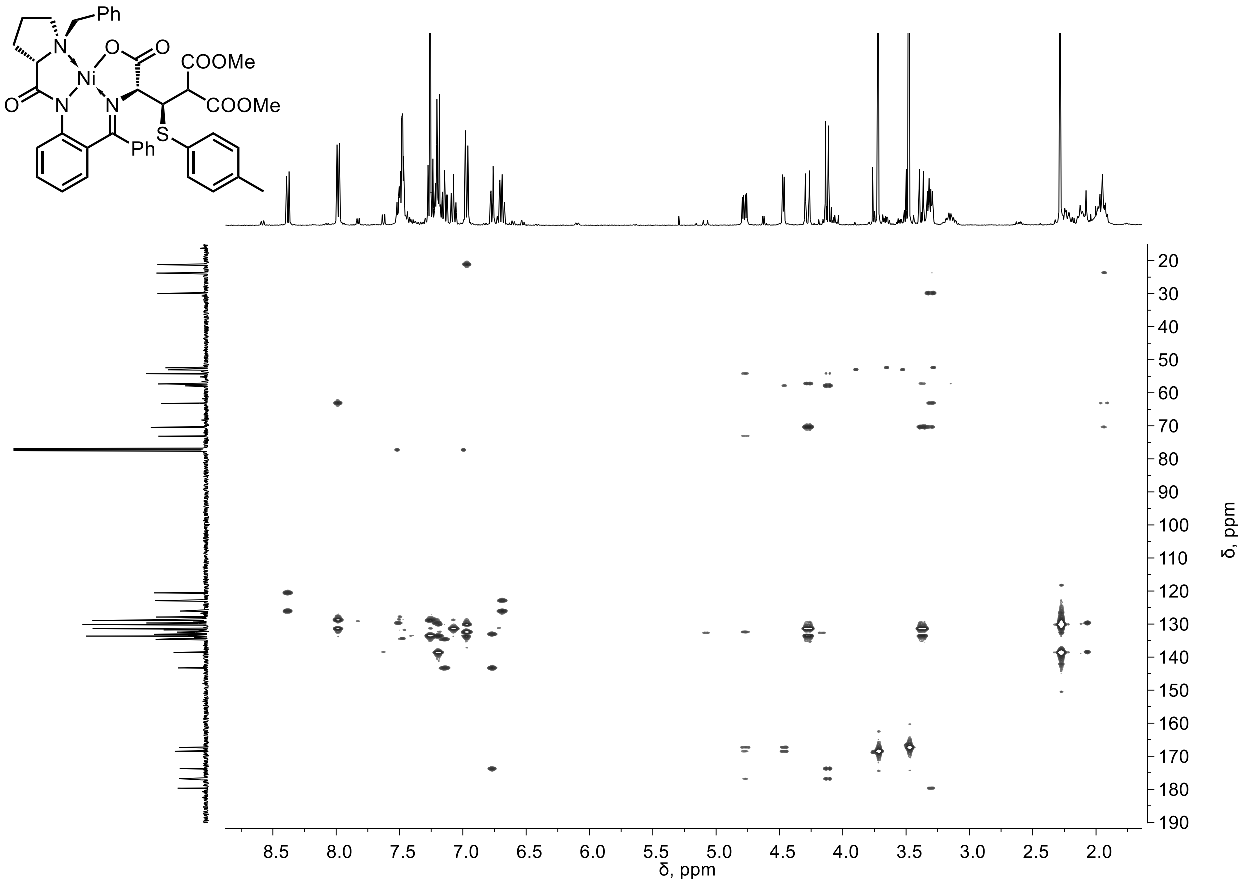

*50. NOESY spectrum of complex (R,R)-**10***

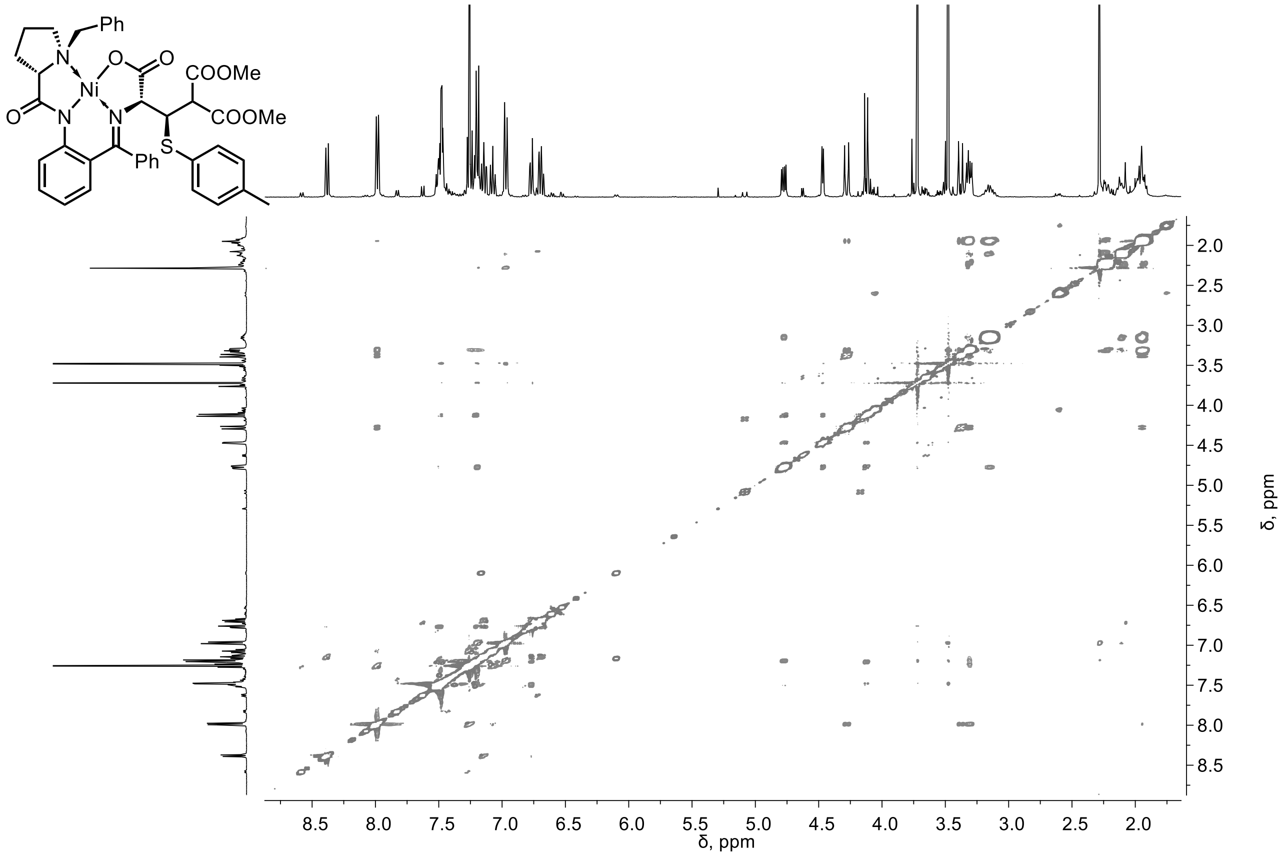

*51. ^{1}H NMR spectrum of complex (R,S)-**11***

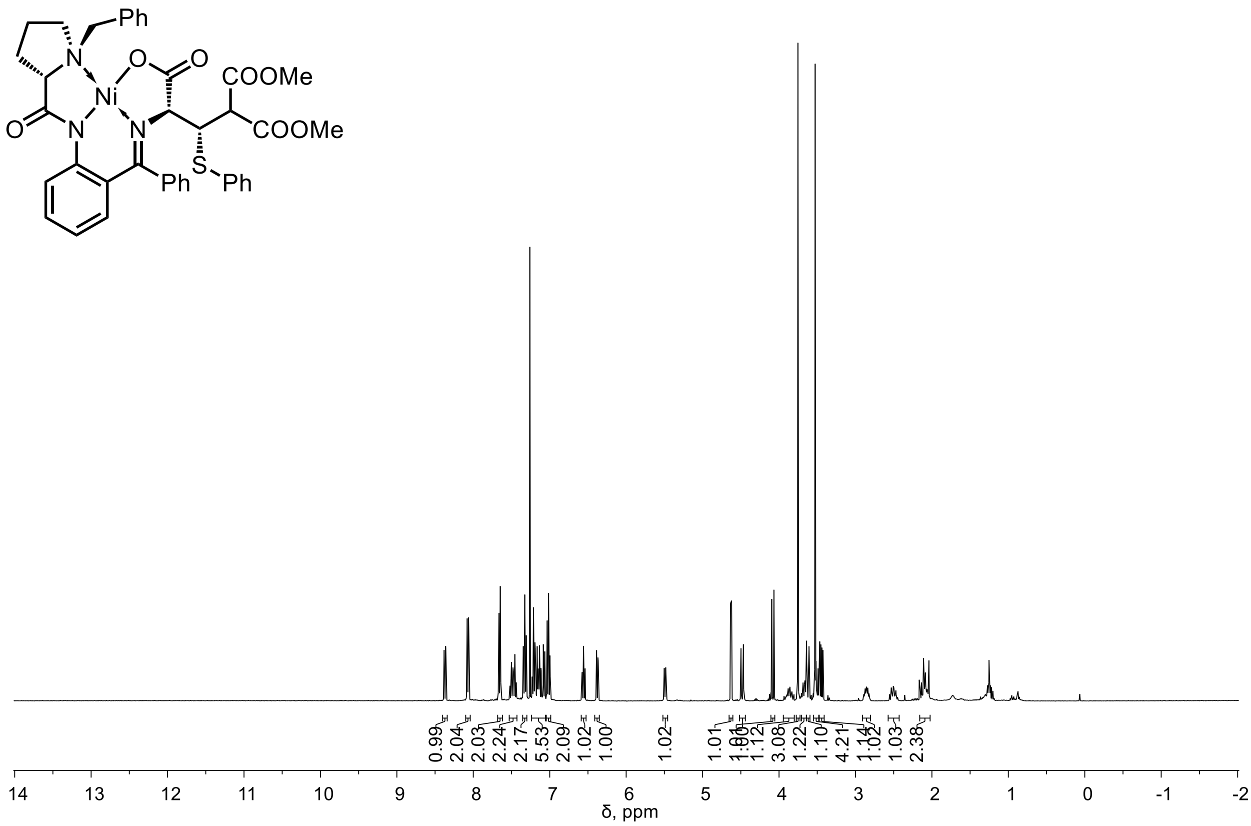

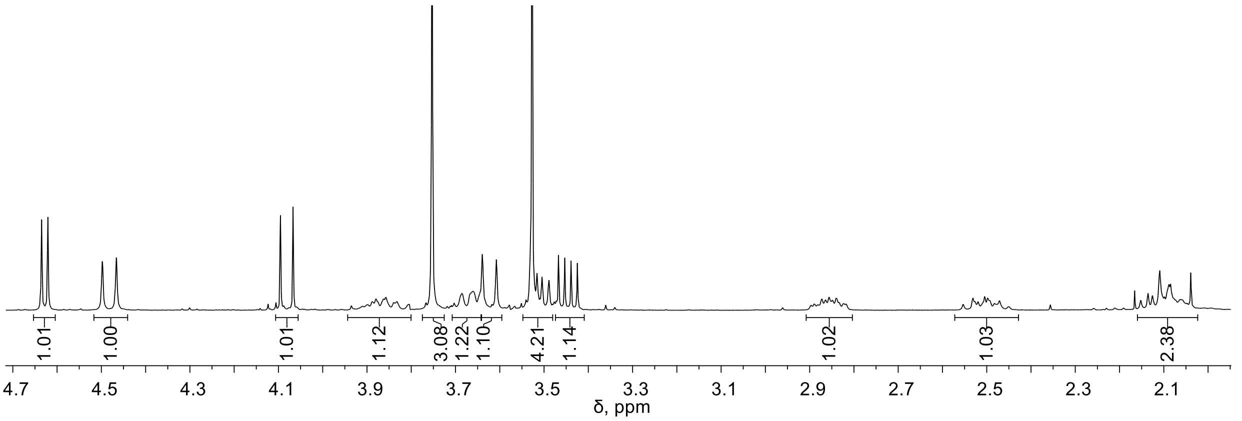

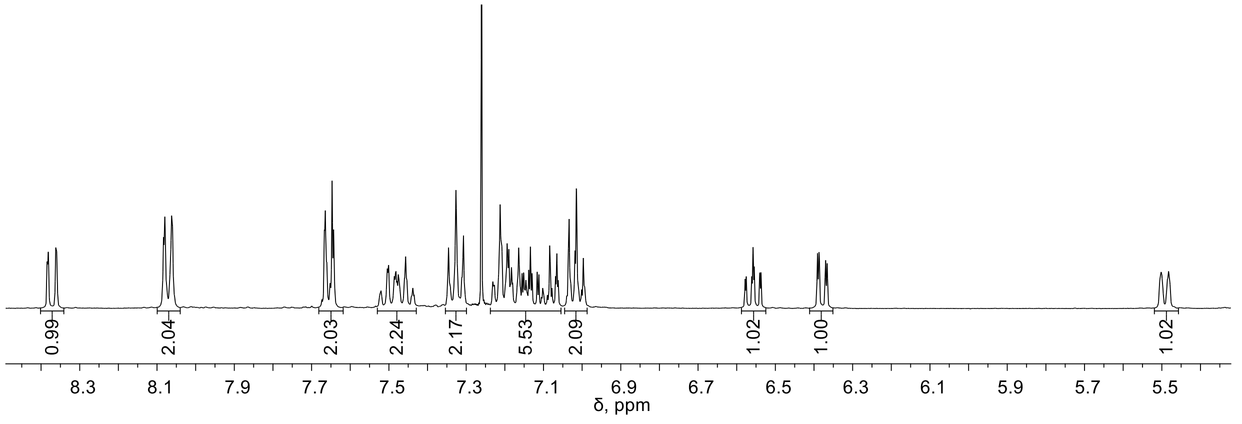

52. ^{13}C NMR spectrum of complex (R,S)-**11**

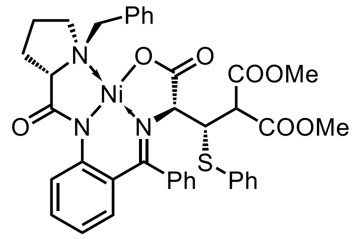

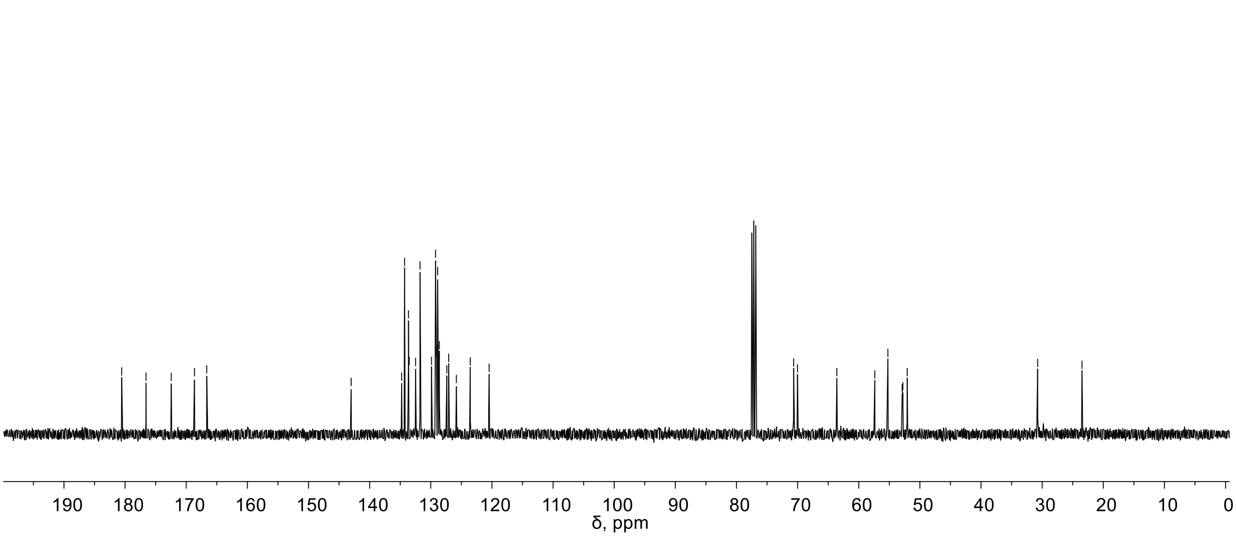

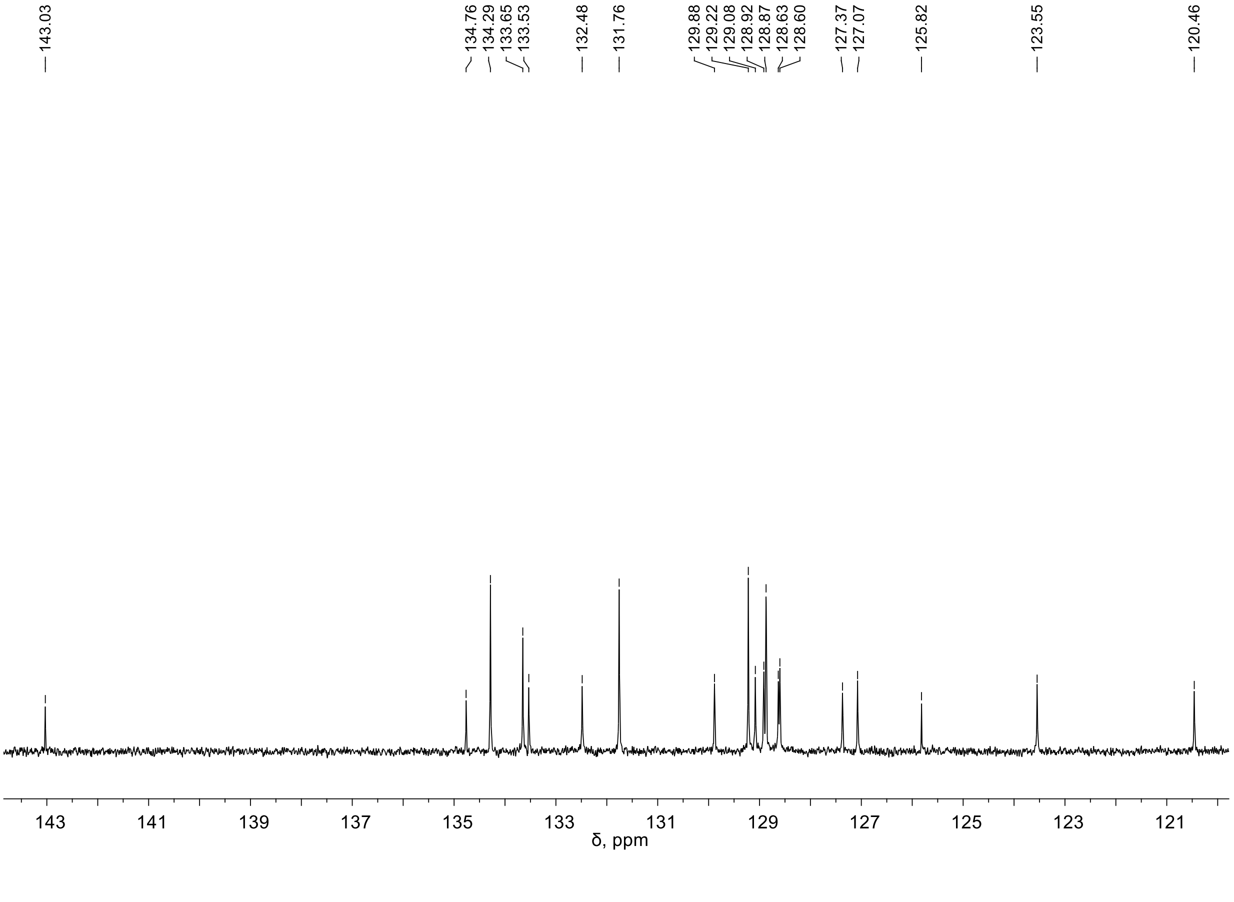

*53. ^{1}H NMR spectrum of complex (R,S)-**12***

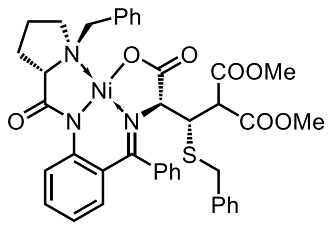

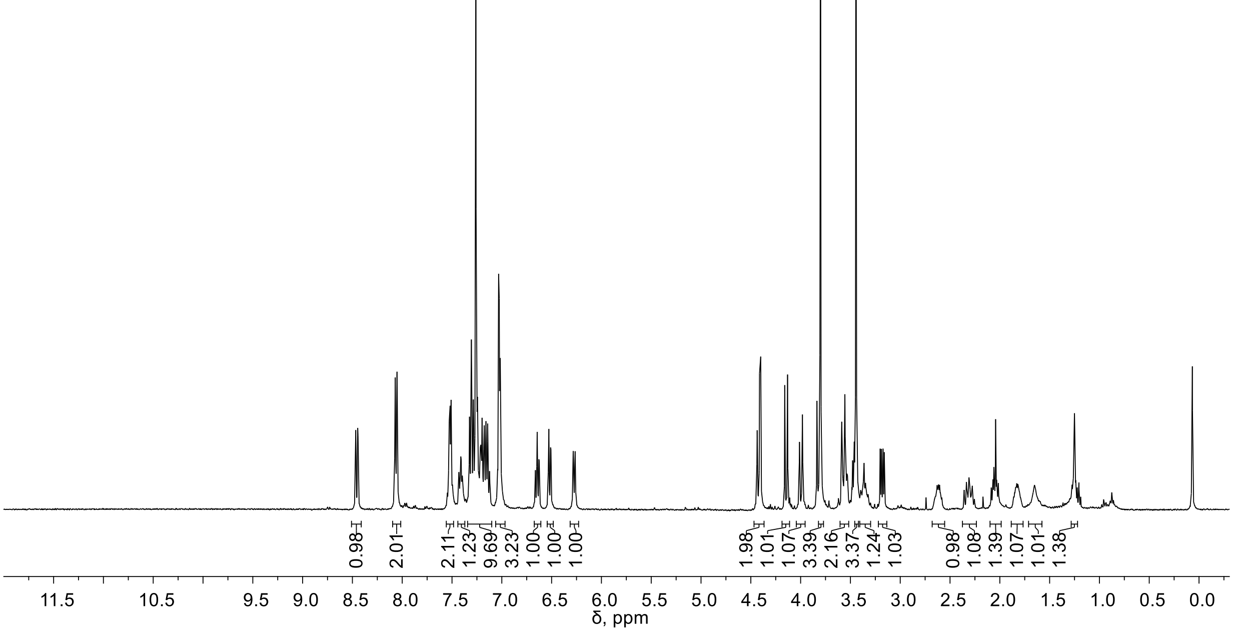

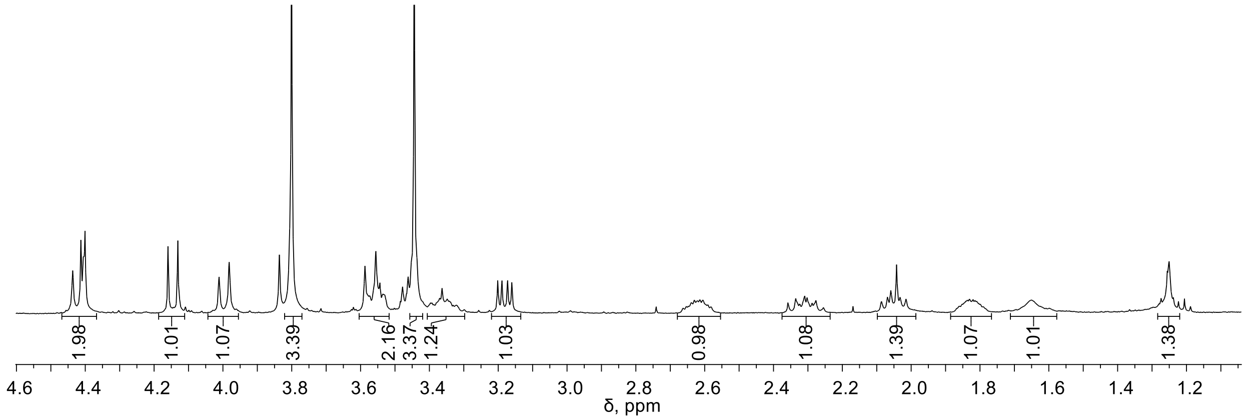

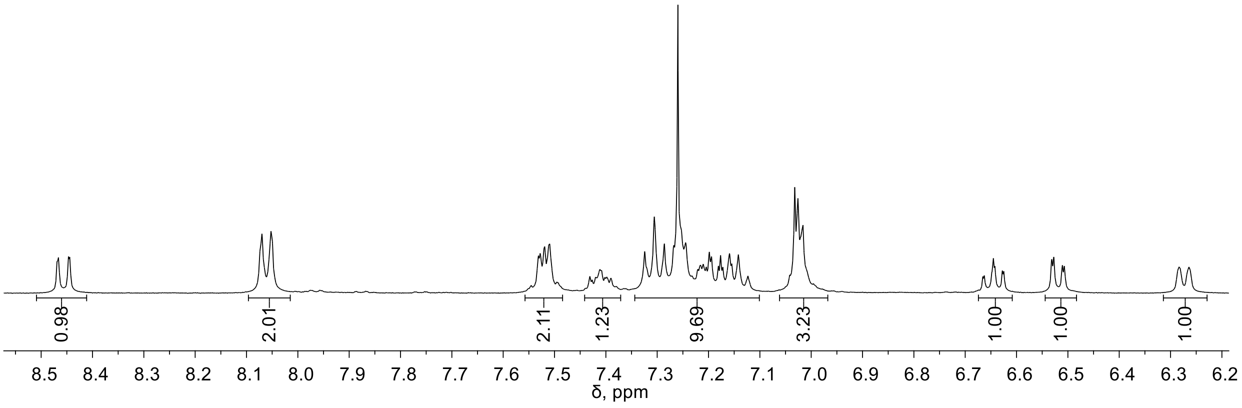

*54. ^{13}C NMR spectrum of complex (R,S)-**12***

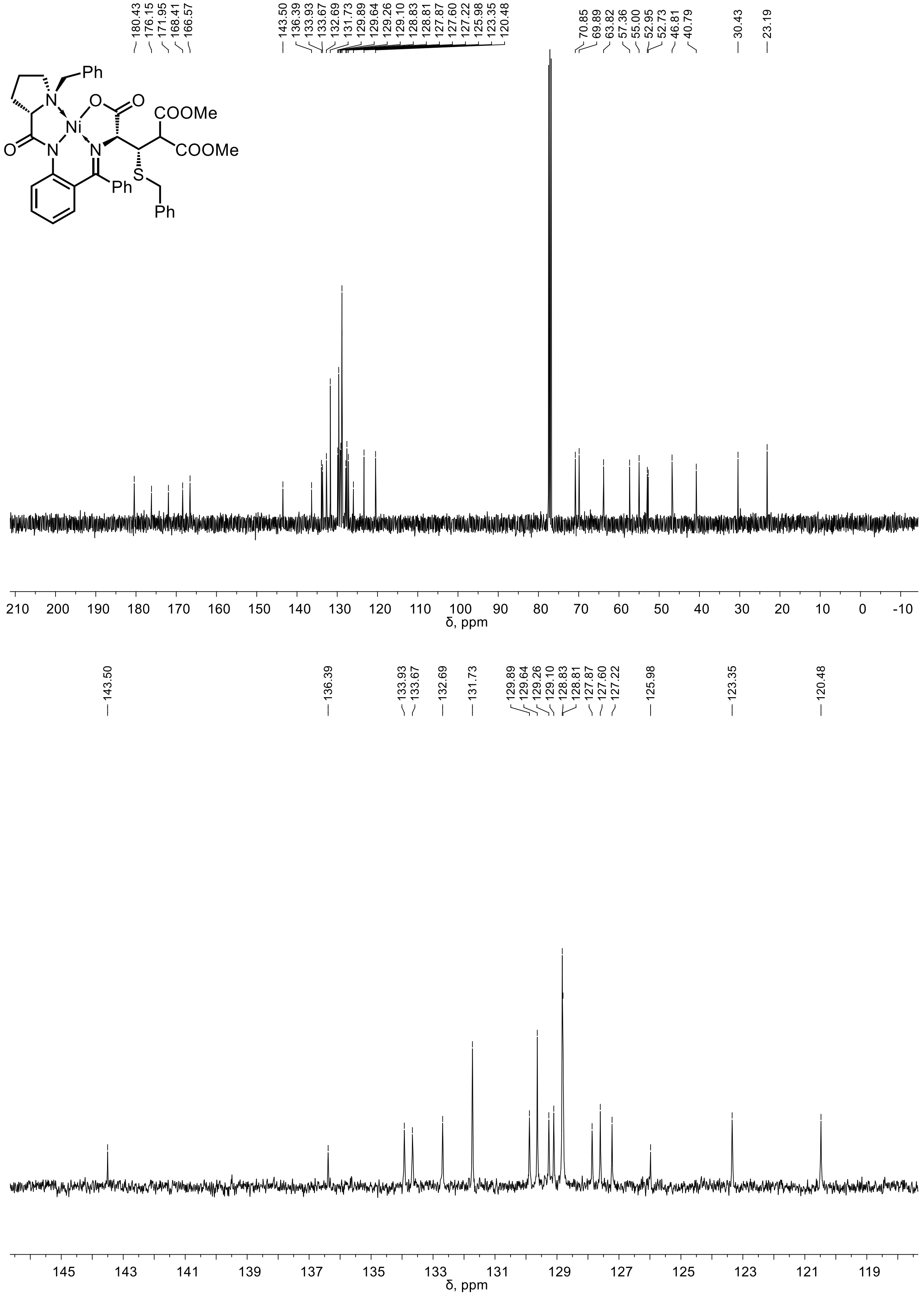

55. *^{1}H NMR spectrum of complex (R,R)-**12***

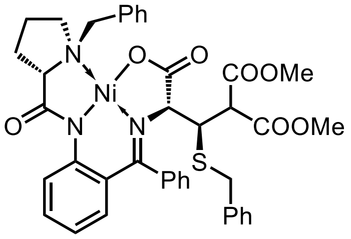

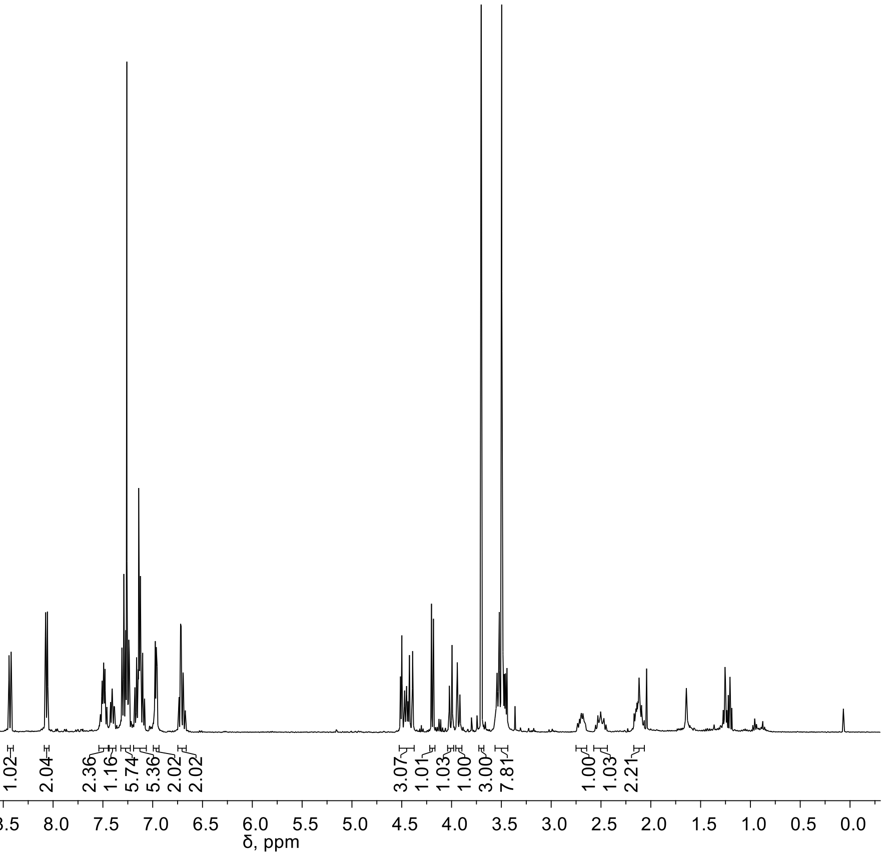

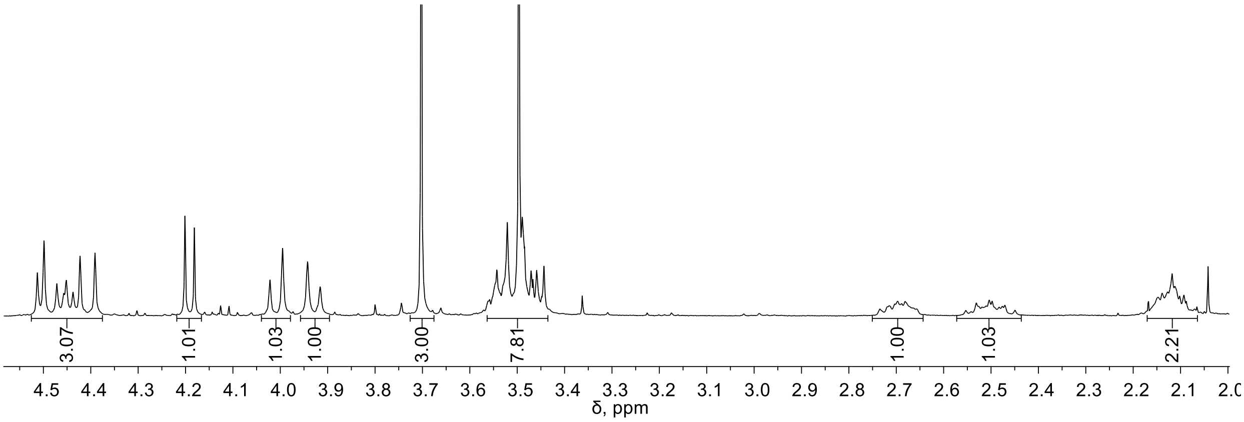

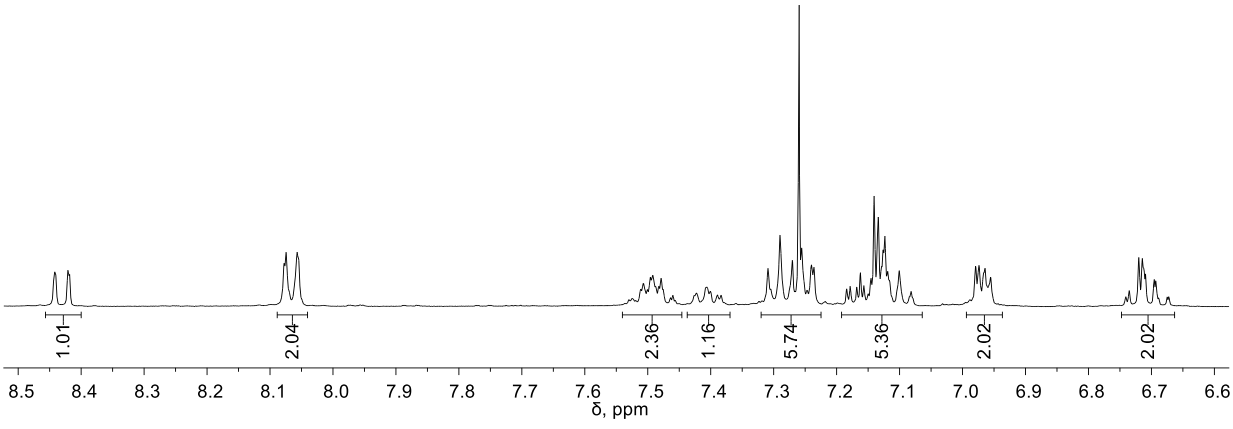

*56. ^{13}C NMR spectrum of complex (R,R)-**12***

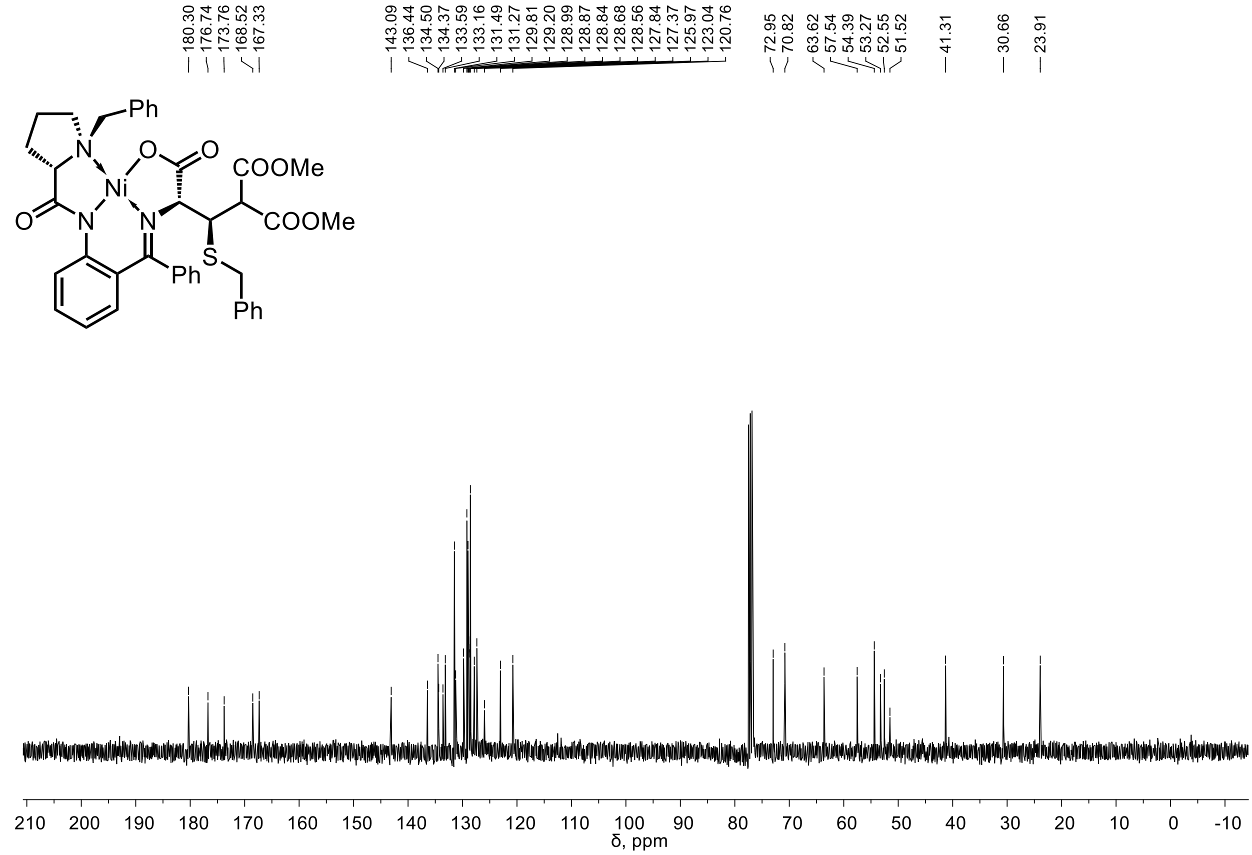

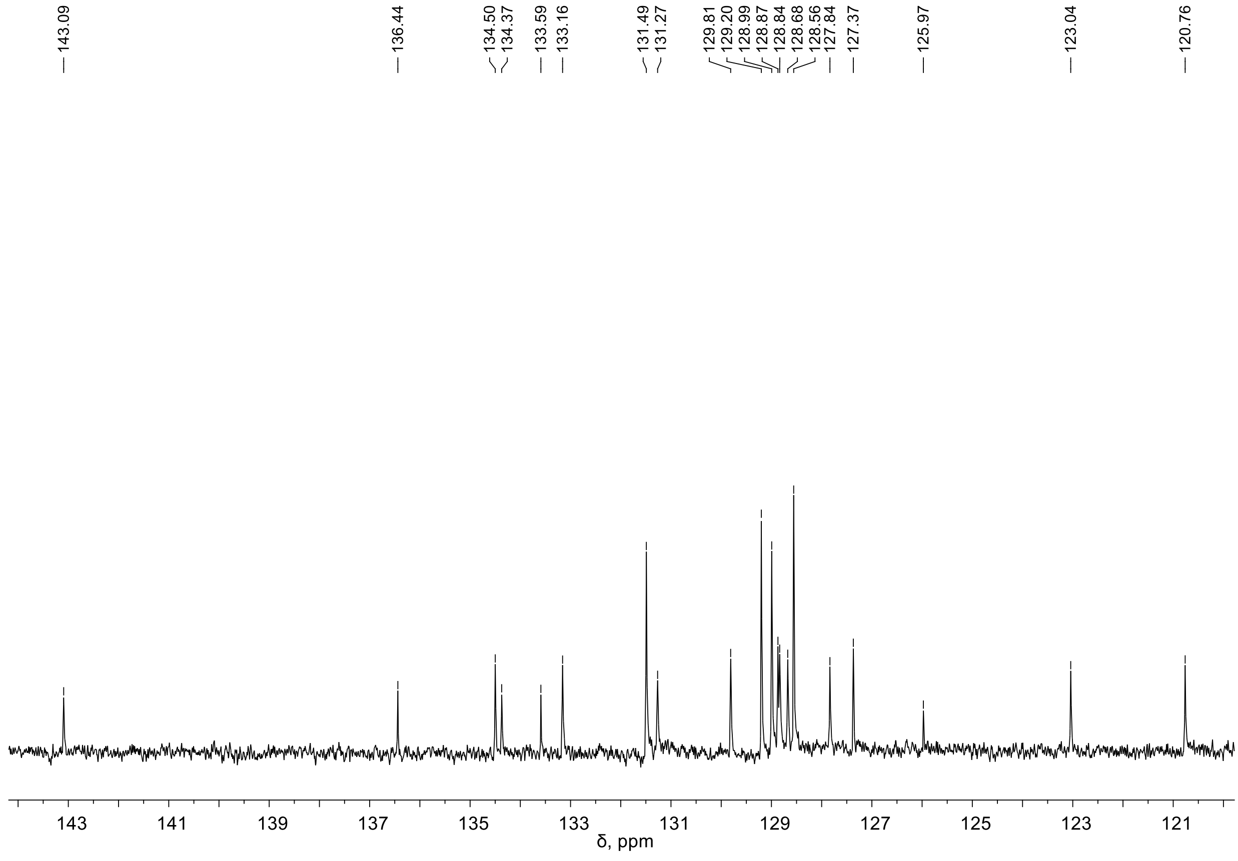

*57. ESI-HRMS data for complex (S)-**4***

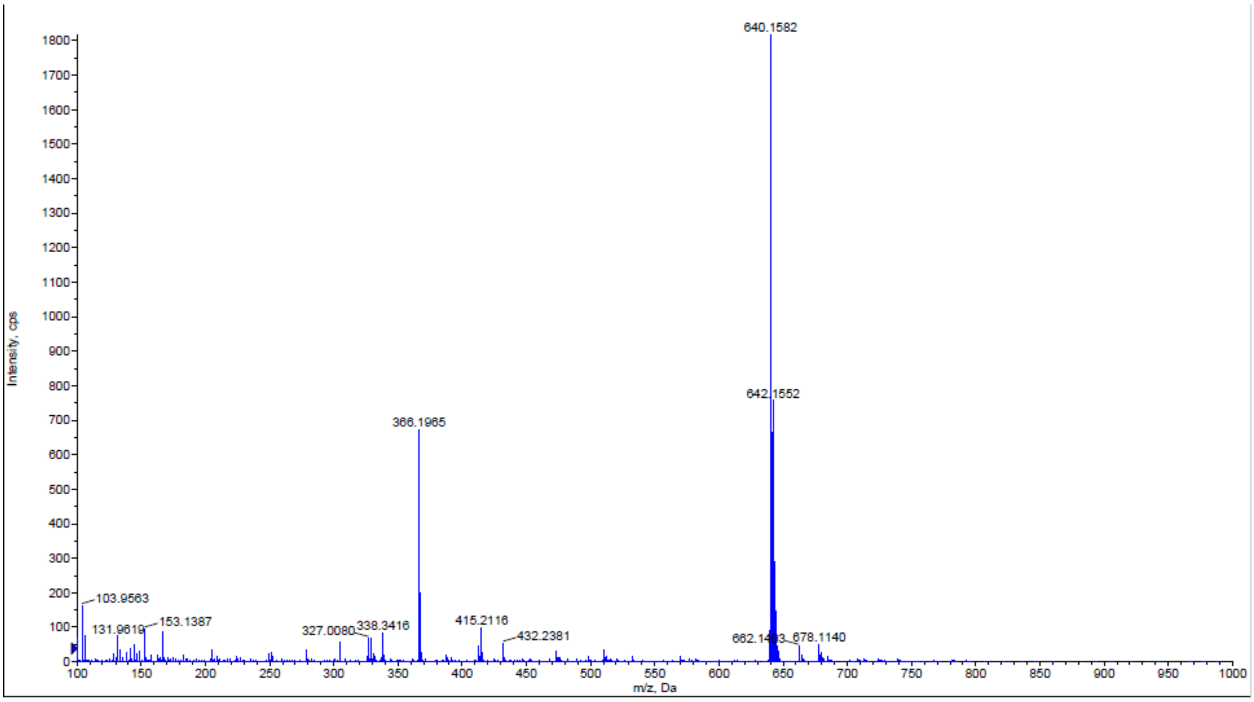

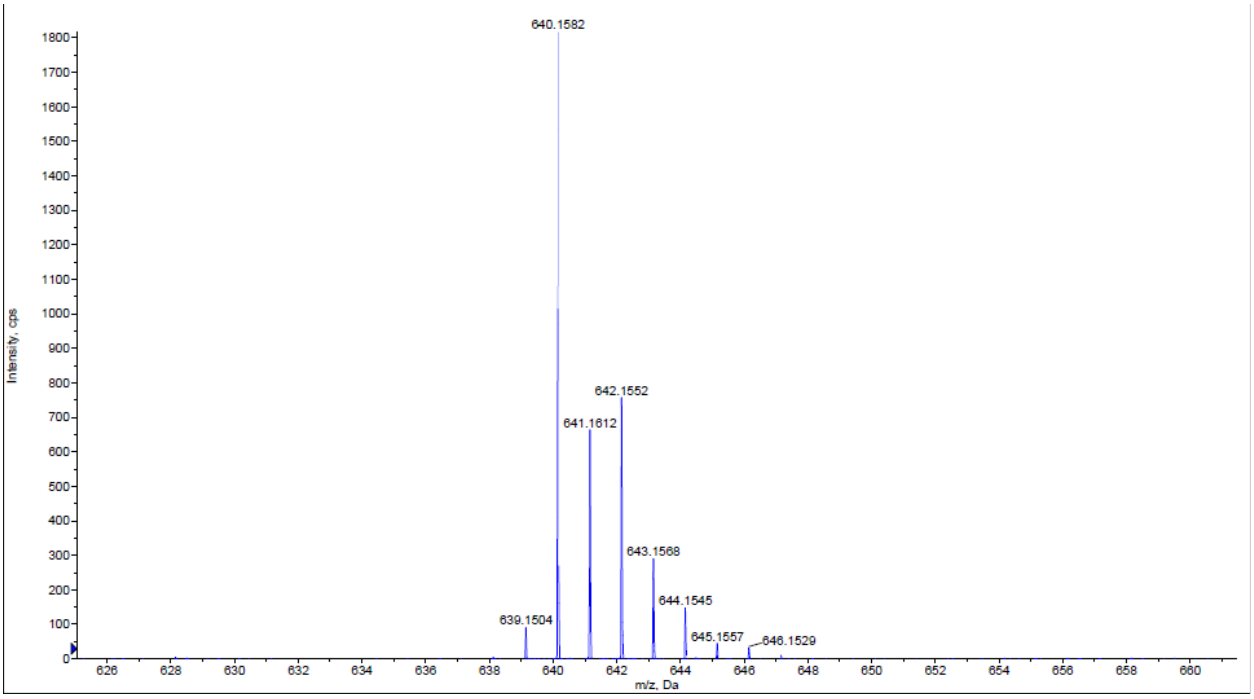

*58. ESI-HRMS data for complex **5**, diastereomer 1*

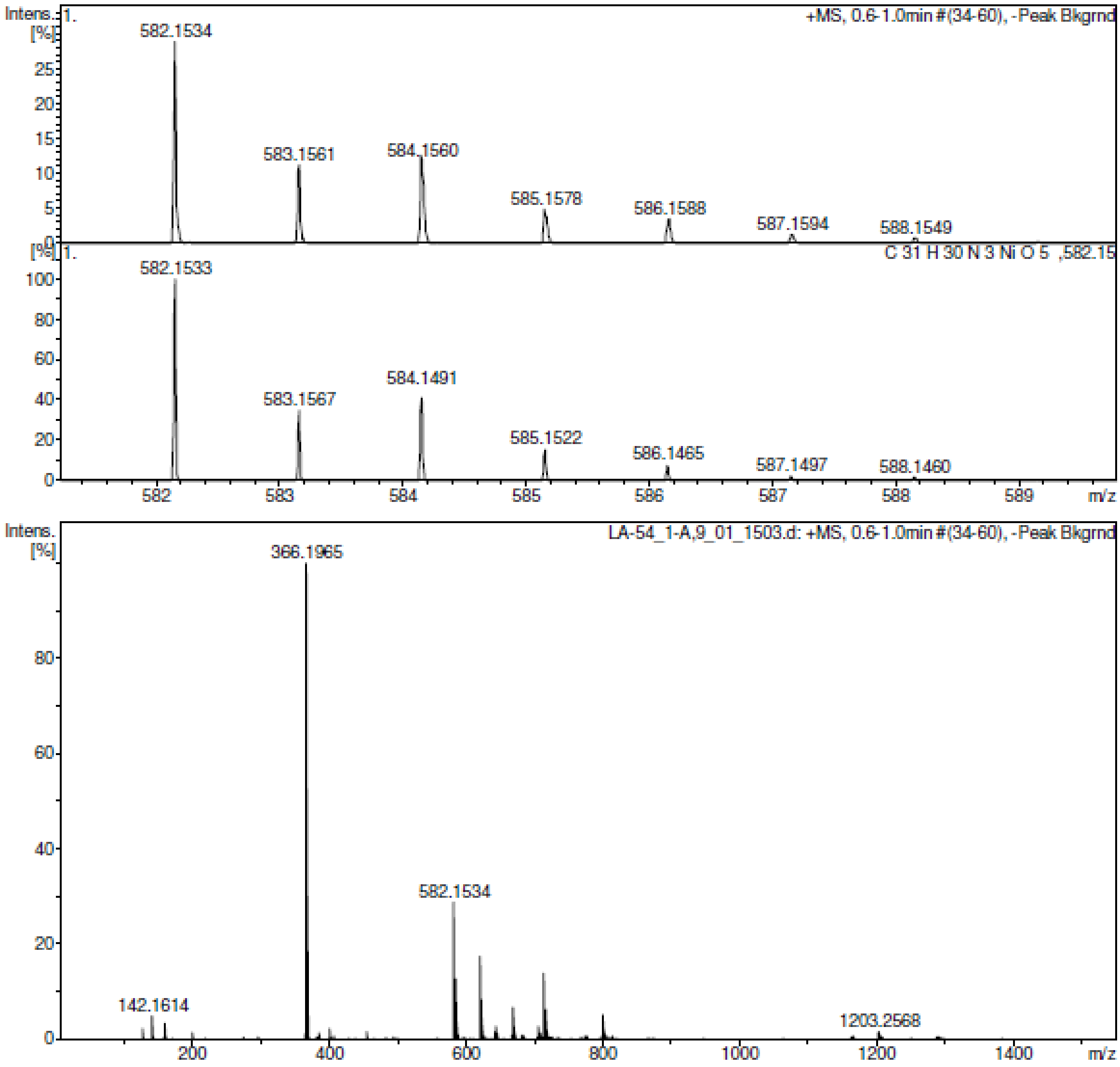

*59. ESI-HRMS data for complex **8***

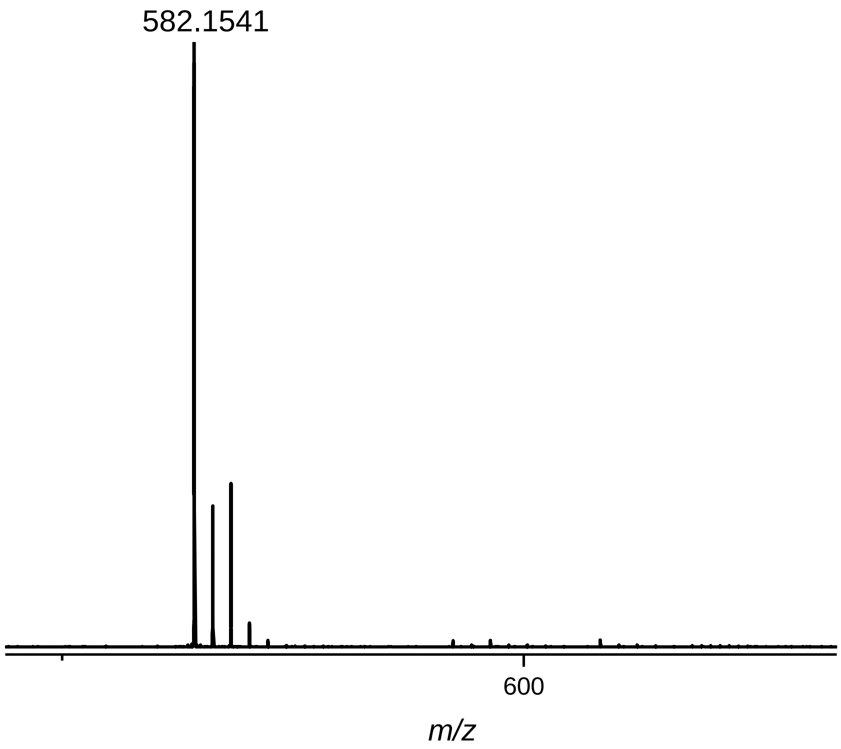

*60. ESI-HRMS data for complex **9***

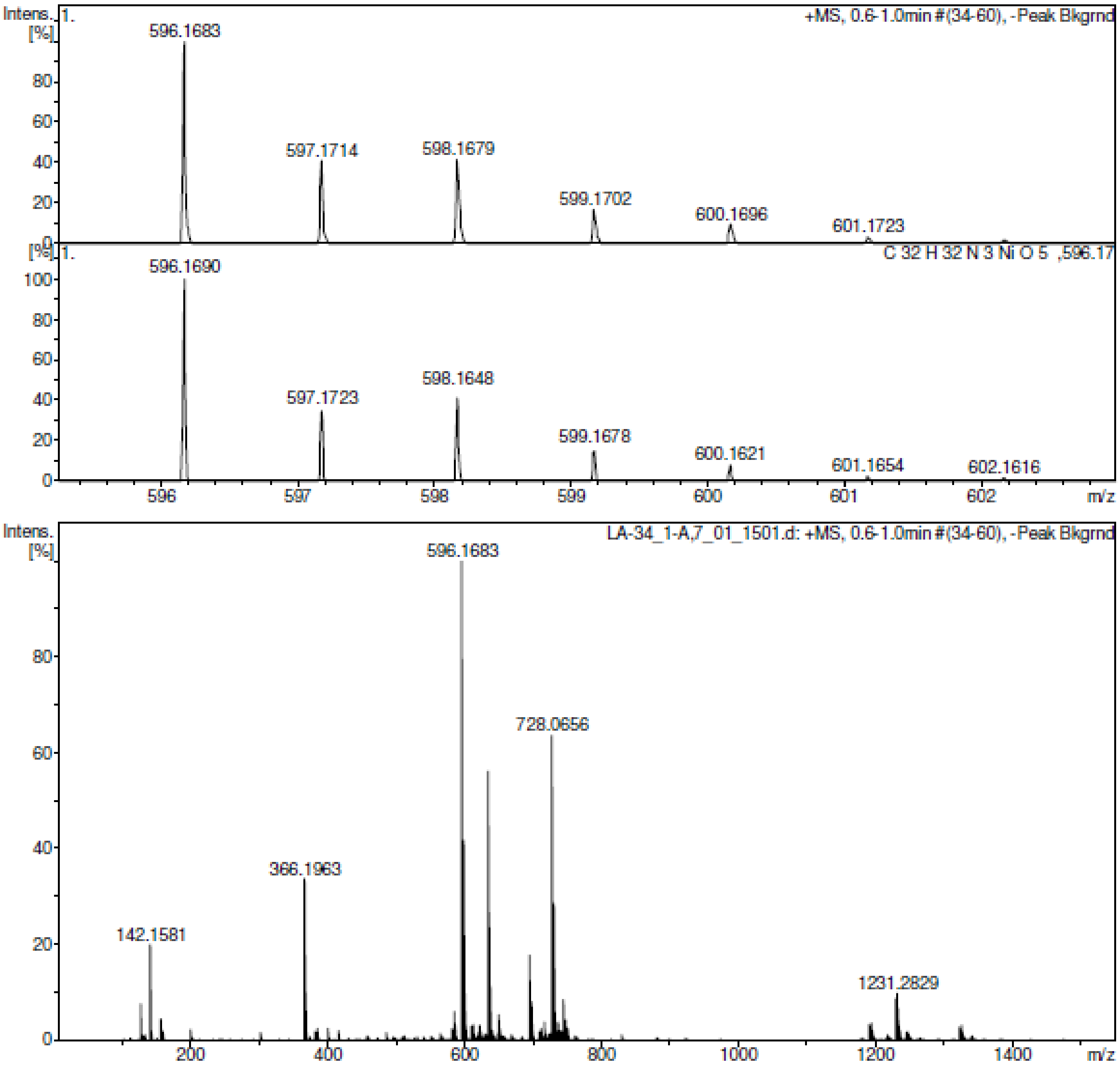

61. *ESI-HRMS data for complex (R,S)-**10***

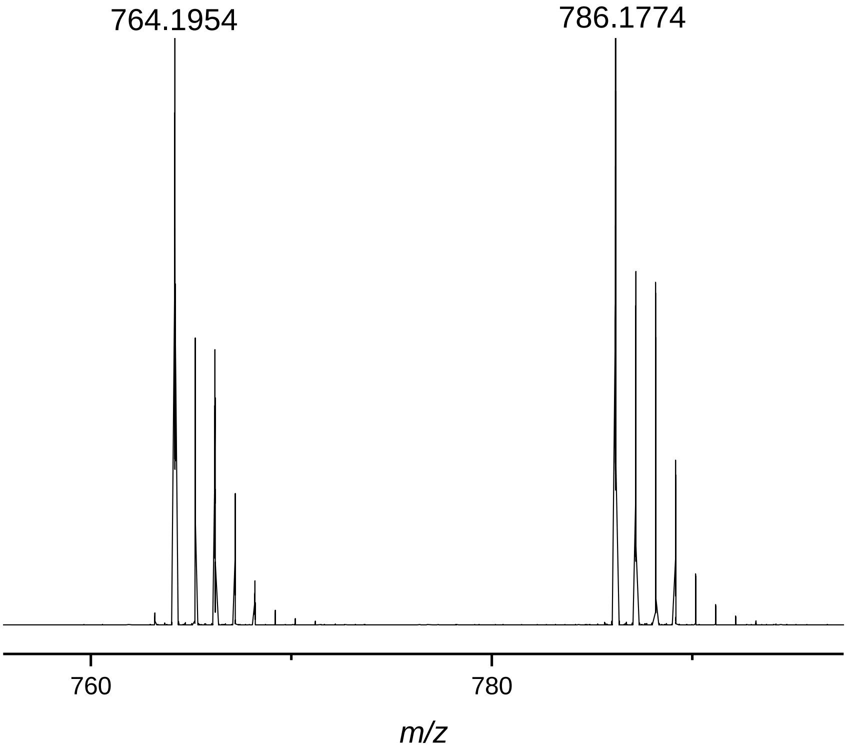

62. Results of the quantum chemical calculations

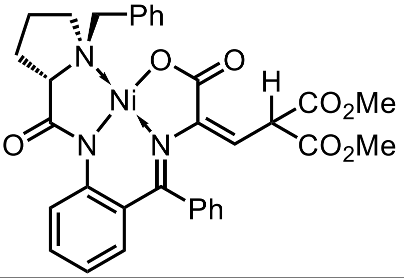

E_e = -3470.401565845330 Hartree

Element	x, Å	y, Å	z, Å
6	1.042189000	-1.402104000	0.697121000
6	1.747990000	-2.196185000	1.618530000
6	1.110668000	-3.053105000	2.484198000
6	-0.276357000	-3.145913000	2.422292000
6	-1.001629000	-2.379957000	1.535671000
6	-0.380249000	-1.460406000	0.667638000
7	-1.105026000	-0.599660000	-0.125584000
6	1.826030000	-0.575329000	-0.210982000
1	2.825036000	-2.121124000	1.664279000
1	1.678940000	-3.640354000	3.192311000
1	-0.805297000	-3.817417000	3.087199000
1	-2.073965000	-2.474359000	1.537251000
6	5.562468000	-0.173128000	-0.052589000
6	5.997593000	-1.371365000	-0.603399000
6	5.071986000	-2.318952000	-1.019611000
6	3.714017000	-2.072420000	-0.883024000
6	3.277710000	-0.864506000	-0.347624000
6	4.205689000	0.084160000	0.070498000
1	3.869905000	1.020932000	0.498242000
1	6.280882000	0.563533000	0.282500000
1	7.057077000	-1.568108000	-0.704435000
1	5.406533000	-3.254521000	-1.448509000
1	2.993170000	-2.814006000	-1.204018000
7	1.286636000	0.397858000	-0.861817000
6	1.908690000	1.200516000	-1.845777000
6	1.397818000	2.611619000	-1.753775000
8	2.005961000	3.543331000	-2.243810000
8	0.274800000	2.692540000	-1.134199000
6	-2.412390000	-0.802758000	-0.444577000
6	-3.040649000	0.423352000	-1.072842000
8	-3.058355000	-1.829496000	-0.343697000
6	-3.022139000	0.338969000	-2.618676000
7	-2.294307000	1.660487000	-0.737904000
1	-4.072189000	0.512117000	-0.718343000
1	-4.034905000	0.175572000	-2.982770000
1	-2.416672000	-0.494517000	-2.975231000
6	-2.731035000	2.299681000	0.525874000
6	-2.585066000	2.569855000	-1.871189000
1	-2.228063000	3.266051000	0.582539000
6	-2.443796000	1.505447000	1.768594000
1	-3.805406000	2.496818000	0.451980000
1	-1.919124000	3.428903000	-1.859793000

1	-3.614022000	2.932056000	-1.767047000
6	-2.461977000	1.686079000	-3.092975000
6	-1.229332000	1.646826000	2.437063000
6	-3.396615000	0.633745000	2.286544000
6	-0.964896000	0.915776000	3.586127000
1	-0.488831000	2.348656000	2.069991000
6	-3.137583000	-0.094995000	3.440443000
1	-4.354365000	0.530456000	1.789980000
1	-3.889969000	-0.768154000	3.831150000
1	-0.015730000	1.034692000	4.092820000
6	-1.919603000	0.041863000	4.090861000
1	-1.715331000	-0.527276000	4.988676000
1	-3.019895000	2.092613000	-3.935245000
1	-1.421659000	1.597797000	-3.409758000
28	-0.445256000	1.046271000	-0.672134000
6	2.664820000	0.731014000	-2.837051000
6	3.243978000	1.551576000	-3.953666000
1	2.874894000	-0.329803000	-2.867525000
6	4.066026000	0.660459000	-4.879278000
6	2.175686000	2.301558000	-4.740966000
1	3.951783000	2.280192000	-3.541755000
8	2.623970000	3.451485000	-5.202650000
8	1.074240000	1.864637000	-4.943840000
8	4.083565000	1.122505000	-6.115558000
8	4.651081000	-0.322344000	-4.513615000
6	1.722202000	4.235473000	-5.976341000
1	2.262088000	5.140685000	-6.242237000
1	1.419932000	3.721075000	-6.889161000
1	0.833180000	4.505968000	-5.405782000
6	4.865486000	0.409510000	-7.076235000
1	4.742188000	0.941427000	-8.015396000
1	5.920904000	0.401931000	-6.804511000
1	4.514916000	-0.615516000	-7.195454000

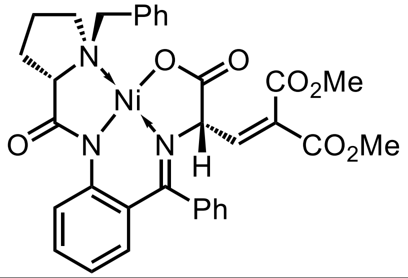

E_e = -3470.401287771888 Hartree

Element	x, Å	y, Å	z, Å
6	0.345627000	-1.859584000	1.800802000
6	1.079505000	-2.676634000	2.678943000
6	0.468353000	-3.512507000	3.583024000
6	-0.922104000	-3.557289000	3.606410000
6	-1.672852000	-2.780940000	2.750955000
6	-1.075270000	-1.893771000	1.832300000
7	-1.820367000	-1.049129000	1.039995000
6	1.107194000	-1.029399000	0.875604000
1	2.159264000	-2.639388000	2.658620000

1	1.059141000	-4.118103000	4.256488000
1	-1.432174000	-4.204011000	4.309580000
1	-2.745374000	-2.846428000	2.813278000
6	4.863276000	-0.945059000	1.187067000
6	5.227529000	-2.026500000	0.395732000
6	4.252002000	-2.766966000	-0.256584000
6	2.912188000	-2.439632000	-0.108144000
6	2.547491000	-1.358434000	0.686045000
6	3.525732000	-0.607686000	1.331475000
1	3.241244000	0.231901000	1.954019000
1	5.620634000	-0.363761000	1.696748000
1	6.271526000	-2.289968000	0.286092000
1	4.531798000	-3.605747000	-0.880146000
1	2.152292000	-3.027274000	-0.607814000
7	0.575450000	-0.049087000	0.238350000
6	1.332295000	0.739193000	-0.734081000
6	0.642667000	2.118014000	-0.840890000
8	1.196317000	3.038104000	-1.397179000
8	-0.527557000	2.147773000	-0.314077000
6	-3.134607000	-1.274089000	0.765314000
6	-3.796837000	-0.085703000	0.104902000
8	-3.770157000	-2.299780000	0.927687000
6	-3.872562000	-0.259933000	-1.431636000
7	-3.032613000	1.163744000	0.323846000
1	-4.806337000	0.025510000	0.512691000
1	-4.906602000	-0.432486000	-1.724460000
1	-3.295913000	-1.119107000	-1.774509000
6	-3.400514000	1.880572000	1.569931000
6	-3.387614000	2.007136000	-0.842620000
1	-2.881108000	2.839188000	1.548321000
6	-3.072875000	1.151381000	2.842631000
1	-4.474155000	2.090778000	1.531845000
1	-2.723362000	2.863201000	-0.917790000
1	-4.410116000	2.375434000	-0.702041000
6	-3.332690000	1.052029000	-2.014097000
6	-1.831099000	1.308883000	3.455544000
6	-4.014990000	0.320827000	3.441714000
6	-1.529231000	0.630476000	4.627353000
1	-1.097386000	1.981585000	3.025540000
6	-3.718251000	-0.355539000	4.618161000
1	-4.993286000	0.206108000	2.989652000
1	-4.462150000	-0.998453000	5.071228000
1	-0.558864000	0.760288000	5.089177000
6	-2.472579000	-0.206015000	5.210810000
1	-2.238237000	-0.735058000	6.125767000
1	-3.929223000	1.411574000	-2.851375000
1	-2.309101000	0.935618000	-2.374043000
28	-1.177551000	0.554642000	0.351600000
6	2.212722000	-0.214914000	-2.924454000
6	3.650129000	0.094885000	-2.671914000
6	1.841623000	-0.878369000	-4.208662000
8	2.708812000	-0.587049000	-5.163495000

8	0.857135000	-1.551832000	-4.366878000
8	4.406592000	-0.948793000	-2.967005000
8	4.065566000	1.140234000	-2.247360000
6	2.486895000	-1.162256000	-6.450360000
1	3.304671000	-0.818015000	-7.077492000
1	2.501791000	-2.251548000	-6.409165000
1	1.541674000	-0.831446000	-6.881175000
6	5.817375000	-0.797351000	-2.830559000
1	6.246542000	-1.775316000	-3.031428000
1	6.207495000	-0.080926000	-3.555138000
1	6.099791000	-0.481401000	-1.827514000
6	1.232230000	0.060652000	-2.062716000
1	2.365491000	0.889417000	-0.428890000
1	0.227319000	-0.246342000	-2.339623000

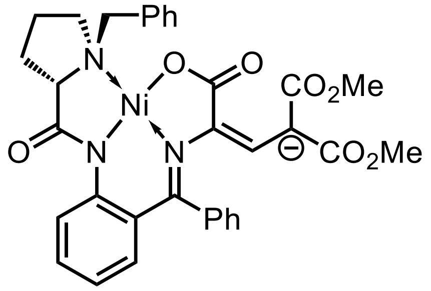

E_e = -3469.918110600138Hartree

Element	x, Å	y, Å	z, Å
6	0.360325000	-1.798084000	1.782495000
6	1.120022000	-2.530239000	2.711149000
6	0.546812000	-3.368345000	3.643153000
6	-0.835417000	-3.508567000	3.650193000
6	-1.615225000	-2.795802000	2.762179000
6	-1.056333000	-1.902400000	1.828699000
7	-1.837847000	-1.084202000	1.036276000
6	1.085970000	-0.993251000	0.794834000
1	2.195790000	-2.426903000	2.710749000
1	1.167615000	-3.905312000	4.348061000
1	-1.315023000	-4.168351000	4.362808000
1	-2.684339000	-2.918494000	2.807977000
6	4.821945000	-0.564156000	0.865634000
6	5.263856000	-1.791547000	0.386470000
6	4.340862000	-2.765105000	0.029422000
6	2.981255000	-2.513661000	0.150384000
6	2.533870000	-1.282951000	0.623168000
6	3.464169000	-0.310085000	0.981997000
1	3.125292000	0.647323000	1.358870000
1	5.537216000	0.195209000	1.154843000
1	6.324330000	-1.988531000	0.295322000
1	4.678563000	-3.723409000	-0.344013000
1	2.263939000	-3.276908000	-0.125482000
7	0.518846000	-0.032895000	0.138098000
6	1.045599000	0.744978000	-0.909034000
6	0.463349000	2.099013000	-0.881626000
8	0.985947000	3.054719000	-1.433912000
8	-0.618986000	2.194634000	-0.166952000

6	-3.147628000	-1.325417000	0.787894000
6	-3.841130000	-0.127642000	0.172478000
8	-3.770140000	-2.363385000	0.944415000
6	-3.912164000	-0.235663000	-1.369962000
7	-3.108369000	1.130517000	0.446465000
1	-4.853746000	-0.058082000	0.582398000
1	-4.941931000	-0.417941000	-1.672934000
1	-3.316461000	-1.066181000	-1.749078000
6	-3.495986000	1.780580000	1.719047000
6	-3.479446000	2.014490000	-0.682136000
1	-2.992728000	2.747803000	1.747461000
6	-3.169773000	1.003429000	2.964173000
1	-4.572974000	1.976661000	1.684712000
1	-2.829723000	2.884232000	-0.719514000
1	-4.509661000	2.357641000	-0.531512000
6	-3.401992000	1.112691000	-1.894429000
6	-1.938951000	1.157071000	3.599721000
6	-4.110264000	0.145487000	3.526393000
6	-1.648821000	0.455624000	4.760972000
1	-1.205660000	1.845703000	3.195310000
6	-3.825339000	-0.554863000	4.691752000
1	-5.079842000	0.030917000	3.055907000
1	-4.569066000	-1.217136000	5.116434000
1	-0.687257000	0.584956000	5.241214000
6	-2.592735000	-0.403101000	5.310561000
1	-2.368237000	-0.948852000	6.218157000
1	-4.007094000	1.494257000	-2.715919000
1	-2.376138000	1.036818000	-2.257197000
28	-1.231599000	0.564570000	0.418903000
6	1.931645000	0.275811000	-1.866566000
6	2.334763000	0.813358000	-3.082357000
1	2.367021000	-0.698227000	-1.677439000
6	3.287205000	0.053632000	-3.860197000
6	1.642064000	1.939206000	-3.760543000
8	2.451256000	2.964076000	-4.024548000
8	0.488737000	1.912471000	-4.116138000
8	3.395204000	0.520903000	-5.121419000
8	3.925677000	-0.914979000	-3.489417000
6	1.887048000	4.058108000	-4.724777000
1	2.683235000	4.790790000	-4.842655000
1	1.526493000	3.774108000	-5.716160000
1	1.066685000	4.517406000	-4.172106000
6	4.316361000	-0.129378000	-5.976408000
1	4.256229000	0.383440000	-6.934742000
1	5.343138000	-0.061064000	-5.610557000
1	4.072304000	-1.182195000	-6.129766000
